# Design, synthesis, and anti-cancer evaluation of new pyrido[2,3-d]pyrimidin-4(3H)-one derivatives as potential EGFRWT and EGFRT790M inhibitors and apoptosis inducers

**DOI:** 10.1080/14756366.2022.2062752

**Published:** 2022-07-12

**Authors:** Heba S. A. Elzahabi, Eman S. Nossier, Rania A. Alasfoury, May El-Manawaty, Sara M. Sayed, Eslam B. Elkaeed, Ahmed M. Metwaly, Mohamed Hagras, Ibrahim H. Eissa

**Affiliations:** a Pharmaceutical Medicinal Chemistry & Drug Design Department, Faculty of Pharmacy (Girls), Al-Azhar University, Cairo, Egypt; b Pharmacognosy Department, National Research Centre, Dokki, Cairo, Egypt; c Biochemistry and Molecular Biology Department, Faculty of Pharmacy (Girls), Al-Azhar University, Cairo, Egypt; d Department of Pharmaceutical Sciences, College of Pharmacy, AlMaarefa University, Riyadh, Saudi Arabia; e Pharmacognosy and Medicinal Plants Department, Faculty of Pharmacy (Boys), Al-Azhar University, Cairo, Egypt; f Biopharmaceutical Products Research Department, Genetic Engineering and Biotechnology Research Institute, City of Scientific Research and Technological Applications (SRTA-City), Alexandria, Egypt; g Pharmaceutical Organic Chemistry, Faculty of Pharmacy (Boys), Al-Azhar University, Cairo, Egypt; h Pharmaceutical Medicinal Chemistry & Drug Design Department, Faculty of Pharmacy (Boys), Al-Azhar University, Cairo, Egypt

**Keywords:** Anti-proliferative, apoptosis, docking studies, EGFR inhibitors, pyrido[2,3-d]pyrimidin-4(3H)-one

## Abstract

A new series of pyrido[2,3-d]pyrimidin-4(3H)-one derivatives having the essential pharmacophoric features of EGFR inhibitors has been designed and synthesised. Cell viability screening was performed for these compounds against A-549, PC-3, HCT-116, and MCF-7 cell lines at a dose of 100 μM. The highest active derivatives (**8a, 8 b, 8d, 9a,** and **12b**) were selected for IC_50_ screening. Compounds **8a, 8 b,** and **9a** showed the highest cytotoxic activities and were further investigated for wild EGFR^WT^ and mutant EGFR^T790M^ inhibitory activities. Compound **8a** showed the highest inhibitory activities against EGFR^WT^ and EGFR^T790M^ with IC_50_ values of 0.099 and 0.123 µM, respectively. In addition, it arrested the cell cycle at pre-G1 phase and induced a significant apoptotic effect in PC-3 cells. Furthermore, compound **8a** induced a 5.3-fold increase in the level of caspase-3 in PC-3 cells. Finally, docking studies were carried out to examine the binding mode of the synthesised compounds against both EGFR^WT^ and EGFR^T790M^.

## 1. Introduction

According to WHO, cancer was the direct cause of 10 million deaths in 2020 and the cost of cancer treatment globally was US$1.16 trillion in 2010^1^. Several internal and external factors can cause cancer. The most well-known factors are hormonal disorders, genetic mutations, radiations, smoking tobacco, metals, polluted food, chemicals, and infectious organisms^2–4^. Resistance against anticancer drugs is considered one of the most serious problems in cancer management^5^. Due to the high residence of many cancer types, the discovery of new anticancer agents with high effect, less resistance, and fewer side effects is an urgent need.

Protein kinases (PKs) are a group of enzymes that are responsible for the transference of phosphate from ATP molecule to tyrosine, serine and/or threonine amino acids in protein substrates^6^^,^^7^. Furthermore, PKs promote cellular signalling processes such as cell growth regulation, differentiation, migration, and metabolism^8^. PKs have been found to be overexpressed in a variety of human malignancies^9^. Accordingly, the inhibition of PKs has emerged as a selective method for killing cancer cells^10^. Receptor tyrosine kinases (RTKs) are vital category protein kinases. About 20 different RTKs have been discovered that have similar structures^11^.

The epidermal growth factor receptor (EGFR) belongs to the RTKs family that stimulates differentiation and proliferation of cells after the binding of its specific active ligand^12^. EGFR structure has an extracellular part (at the surface of the cells) and an intracellular part. The activation of the outer part leads to an activation of the intracellular region of the receptor and a phosphorylation of the intracellular substrates^13^. This step facilitates cell growth, synthesis of DNA, and the expression of oncogenes^14^. It was reported that EGFR is over-expressed and implicated in the pathogenesis and progression of various human carcinomas^15^. In many patients, resistance against cancer therapy arises from an acquired mutation in the EGFR kinase domain (T790M). Such mutant EGFR is called EGFR^T790M^^16^. Thus, EGFRs (wild and mutant types) are interesting biological targets for the discovery of new anticancer agents^17^^,^^18^.

**Figure 1.**
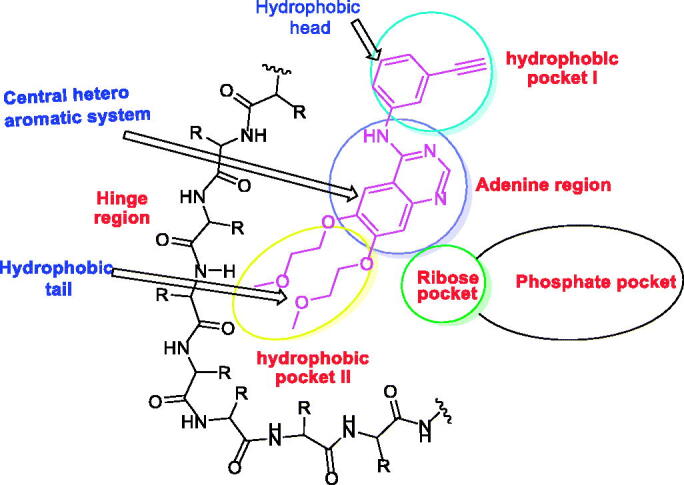
The essential pharmacophoric features of erlotinib as an EGFR inhibitor occupying three pockets in the ATP binding site based on Reference^22^.

The ATP binding site of EGFR consists of five regions; an adenine-binding pocket, a sugar region (ribose binding pocket), a hydrophobic region I, a hydrophobic region II, and a phosphate-binding region^19–21^. Most of the reported EGFR inhibitors are ATP-competitive inhibitor small molecules that have specific moieties to occupy the adenine-binding pocket, the hydrophobic region I, and the hydrophobic region II^10^ (Figure 1).

EGFR inhibitors have a specific Y-shaped structure^23^. In addition, the structure of EGFR inhibitors should comprise many essential pharmacophoric features^24^. Each feature binds at a specific region in the ATP binding site. For example, a flat hetero aromatic system is an essential feature of EGFR inhibitor to occupy the adenine binding pocket of the ATP binding site. Such hetero structure can form hydrogen bonds with some amino acids as Met769, Thr790, and Thr854^25^. Also, a terminal hydrophobic head of the EGFR inhibitor can occupy the hydrophobic region I forming many hydrophobic interactions^24^. Finally, a hydrophobic tail be buried in the hydrophobic region II producing high affinity^19^^,^^26^.

**Figure 2.**
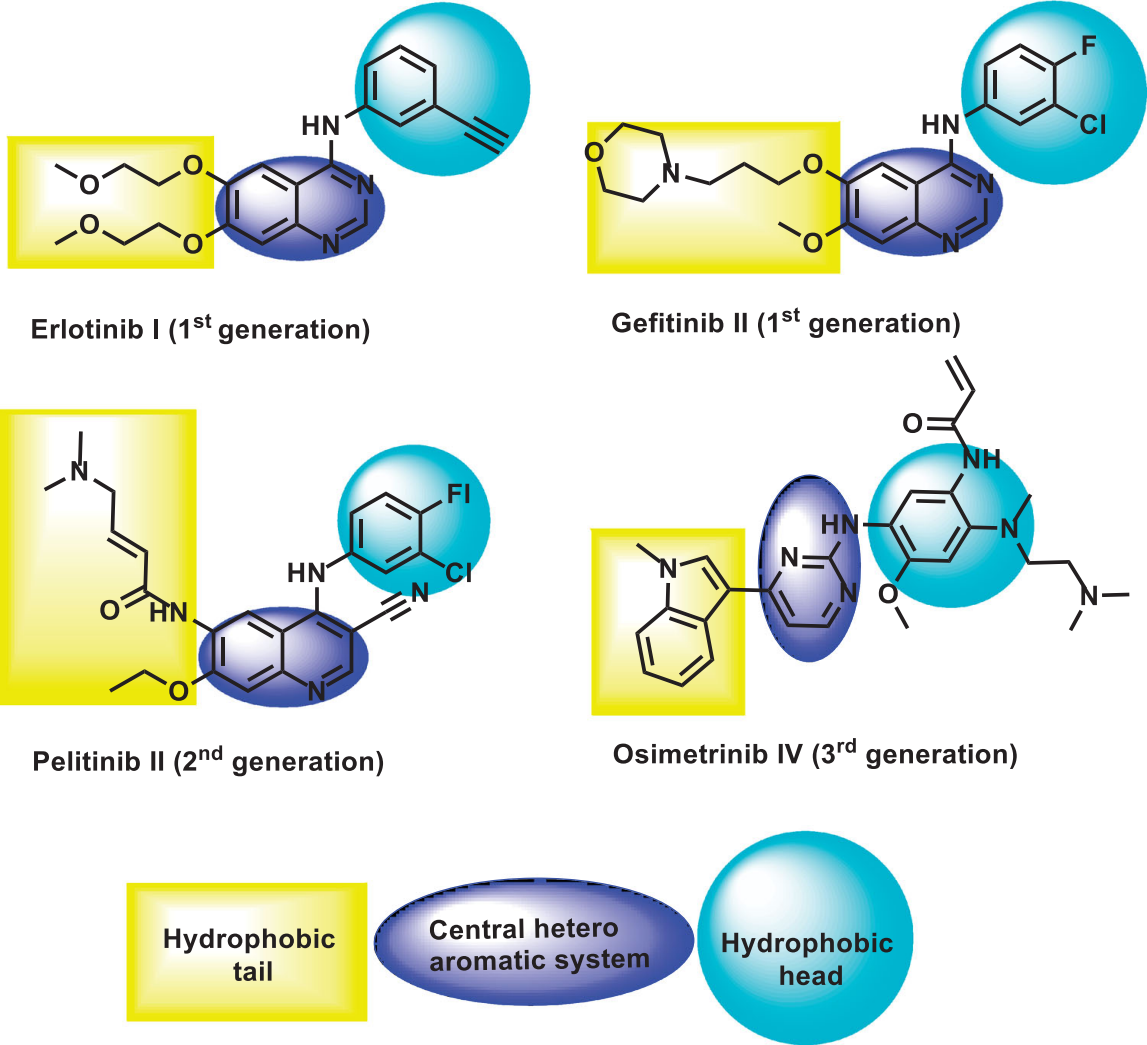
Some reported EGFR-TK inhibitors and their basic pharmacophoric features.

Till now, three generations of EGFR inhibitors were approved by the FDA (Figure 2). Erlotinib I^27^ and gefitinib II^28^ are examples of the first generation. The generated mutation in EGFR led to the acquired drug resistance and reduced efficacy in cancer treatment^29^. The mutant form of protein (EGFR^T790M^) resists the affinity of ATP-competitive inhibitors^30^. The second-generation of EGFR inhibitors was approved to overcome the drug resistance that was induced by EGFR^T790M^. These inhibitors can form covalent interactions with Cys797 at the ATP binding site^31–33^. Pelitinib III^34^ is a well-known example of this class. Unfortunately, low maximal-tolerated-dose, the major drawback of this class, led to poor clinical outcomes^35^^,^^36^. Osimertinib 5^37^, an example of the third-generation EGFR inhibitors, exhibited greater activities against mutant form (EGFR^T790M^) than the wild form (EGFR^WT^). Recently, toxic epidermal necrolysis was reported upon the administration of olmutinib^38^. Hence, many efforts are still required to reach more potent and less toxic EGFR inhibitors.

Pyrido[2,3-d]pyrimidin-4(3H)-one moiety was utilised before for the synthesis of various anticancer agents^39–42^, and EGFR inhibitors^43^. Interestingly it was included in the discovery of highly specific inhibitors against the mutant EGFR^T790M^^44^.

As an extension of our previous efforts in the design and synthesis of new anticancer agents^45–51^, especially that target RTKs^52^^,^^53^ and EGFR^22^ ^54–58^, we used the pyrido[2,3-d]pyrimidin-4(3H)-one moiety as a building block for the design and synthesis of new anticancer agents targeting the wild EGFR (EGFR^WT^) as well as the mutant EGFR (EGFRT^790M^).

### 1.1. Rationale of molecular design

For years, our team synthesised several EGFR inhibitors which showed promising anticancer activities. In 2018, a series of 1H-pyrazolo[3,4-d]pyrimidine derivatives were synthesised and evaluated for their inhibitory activities against EGFR^WT^ and EGFR^T790M^. compound V potently inhibited the two EGFR types with a good apoptotic effect and arrested the cell cycle at the G_2_/M phase. Such compounds comprise two hetero-aromatic rings (1H-pyrazolo[3,4-d]pyrimidine) to occupy the adenine binding pocket^22^.

In 2019, we designed and synthesised a series of thieno[2,3-d]pyrimidine derivatives as EGFR and HER2 tyrosine kinase inhibitors. Compound VI was the most active member producing significant apoptosis. This compound contains two hetero-aromatic rings (thieno[3,2-d]pyrimidine) to occupy the adenine binding pocket^54^.

In 2020, our team designed and synthesised a new series of pyrimidine-5-carbonitrile derivatives as EGFR inhibitors. Compound VII showed high inhibitory activities against EGFR^WT^ and EGFR^T790M^. In addition, it arrested the cell cycle at the G2/M phase and induced a significant apoptotic effect in HCT-116, HepG-2, MCF-7cells. This compound contains one hetero-aromatic ring (pyrimidine) to occupy the adenine binding pocket^55^.

**Figure 3.**
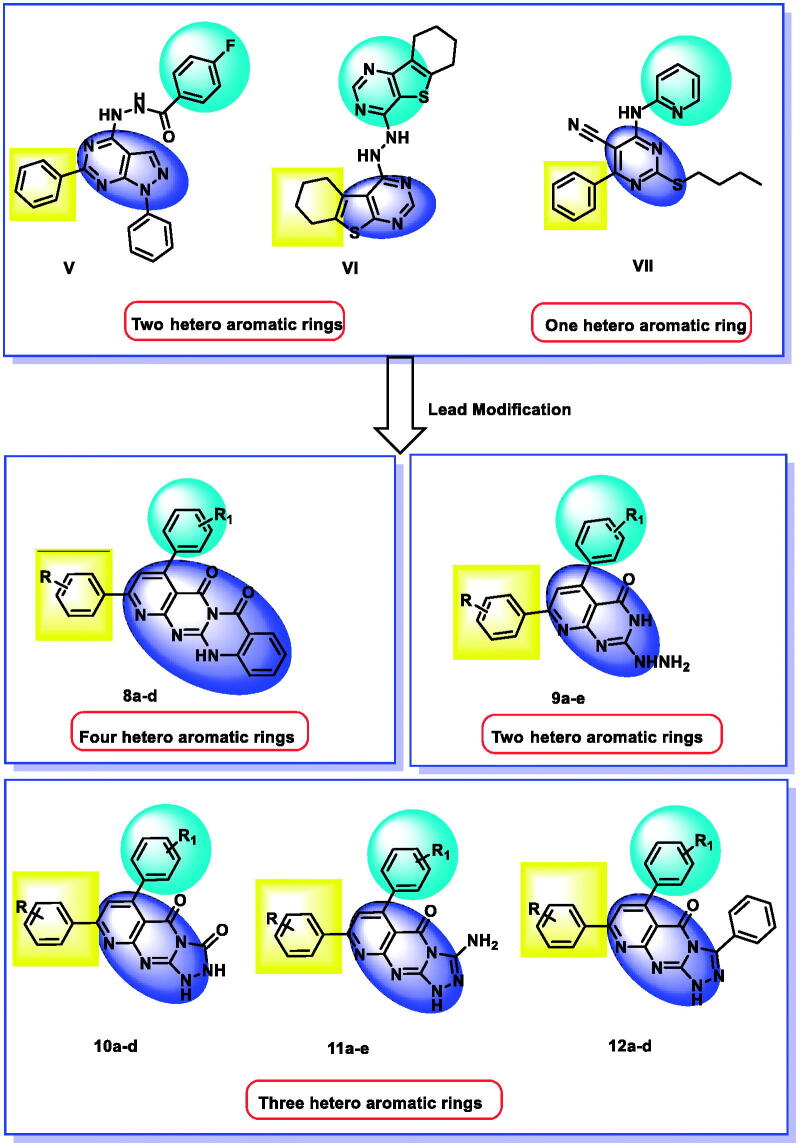
Synthesis of new EGFR inhibitors strategy.

In the current work, we used the previously reported active candidates (V, VI, and VII)^22^^,^^54^^,^^55^ as lead compounds in the design of the new derivatives. The rationale of our molecular design depended on the modification of such compounds to get new EGFR inhibitors. The modification was carried out at three features following the essential features of EGFR inhibitors. Concerning the terminal hydrophobic head and the hydrophobic tail, different substituted benzene rings were used to study the SAR of the synthesised compounds. Regarding the flat hetero-aromatic system, we used three different systems. The first one is pyrido[2,3-d]pyrimidin-4(3H)-one moiety which comprises two hetero-aromatic rings (compounds **9a–e**). The second one is pyrido[2,3-d][1,2,4]triazolo[4,3-a]pyrimidin-5(1H)-one moiety which composes three heteroaromatic rings (compounds **10a–d, 11a–e,** and **12a–d**). The third one is 5H-pyrido[2′,3′:4,5]pyrimido[2,1-b]quinazoline-5,7(12H)-dione moiety which constitutes four hetero-aromatic rings (compounds **8a–d**; Figure 3).

## 2. Results and discussion

### 2.1. Chemistry

In continuation of the previous work^59^, the starting precursor 2-thioxo-2,3-dihydropyrido[2,3-d]pyrimidin-4(1H)-one derivatives 7a–e were afforded via the reaction of the appropriate chalcones 6a–e with 6-aminothiouracil 3. The target compounds were synthesised in acceptable yield as reported^59^. Here in, the structure of the new 2-thioxo-2,3-dihydropyrido[2,3-d]pyrimidin-4(1H)-one derivative 7a was proved by elemental and spectral analyses. ^1^H NMR spectrum showed two D_2_O exchangeable singlet signals at δ 12.51, 13.23 ppm correspond to the two protons of each NH groups. Also, a singlet signal was recorded at δ 7.97 ppm, corresponding to the proton at C6 of pyridopyrimidine ring. The ^13^C NMR spectrum of 7a analogue displayed two characteristic signals at δ 162.29, 175.61 corresponding to carbons of C=O and C=S groups, respectively.

The 5H-pyrido[2′,3′:4,5]pyrimido[2,1-b]quinazoline-5,7(12H)-dione analogues **8a–d** were synthesised through the reaction of compounds **7b–e** with anthranilic acid in the presence of catalytic amount of sodium ethoxide under reflux condition^60^. Their chemical structures were confirmed by elemental and spectral data for example the ^1^H NMR of compound **8d** revealed an increase in the integration of aromatic region at *δ* 6.76–8.15 ppm, and the presence of D_2_O exchangeable singlet signal assigned for one proton of NH group at *δ* 11.64 ppm. The ^13 ^C NMR spectrum showed the characteristic two signals for the two carbons of C=O signals at *δ* 161.45 and 169.46 ppm. The mass spectrum for 8d revealed the expected molecular ion peak at *m/z* of 520. Finally, IR spectrum of 8 b displayed absorption bands at 1693, 1750 and 3410 cm^−1^ corresponding to two C=O and one NH groups, respectively.

The 2-hydrazinopyrido[2,3-d]pyrimidin-4(3H)-one derivatives **9a–e** were depicted through the nucleophilic attack of hydrazine hydrate upon the key derivatives **7a–e** following the reported method^60^. The newly hydrazinyl derivative **9a** was proved by spectral data. The ^1^H NMR spectrum showed two singlet signals at *δ* 8.23, 9.12 ppm assigned for three protons of hydrazinyl group NHNH_2_.

Cyclo-condensation of the 2-hydrazinyl derivative **9a–e** with ethyl chloroformate in dry pyridine produced pyrido[2,3-d][1,2,4]triazolo[4,3-a]pyrimidine-3,5-dione derivatives **10a–d**. The IR spectrum of compound **10d** revealed the presence of three absorption bands at 1708, 3437, and 3425 cm^−1^ assigned for two carbonyl and two NH groups, respectively. The ^1^H NMR spectrum for the same compound showed two D_2_O exchangeable signals at *δ* 9.26, 11.07 ppm assigned for two NH groups. Mass spectrum of compound **10c** showed molecular ion peak at *m/z* of 479 and its isotope at *m/z* of 481.

Reaction of hydrazinyl derivatives **9a–e** with ammonium thiocyanate in glacial acetic acid under reflux afforded 3-aminopyrido[2,3-d][1,2,4]triazolo[4,3-a]pyrimidin-5(1H)-one derivatives **11a–e.** The ^1^H NMR of compound **11a** revealed the presence of two exchangeable singlet signals at 7.09 and 7.33 ppm assigned for NH and NH_2_ groups. Mass spectra of compound **11c** illustrated the expected molecular ion peak at *m/z* of 400.5.

**Scheme 1.**
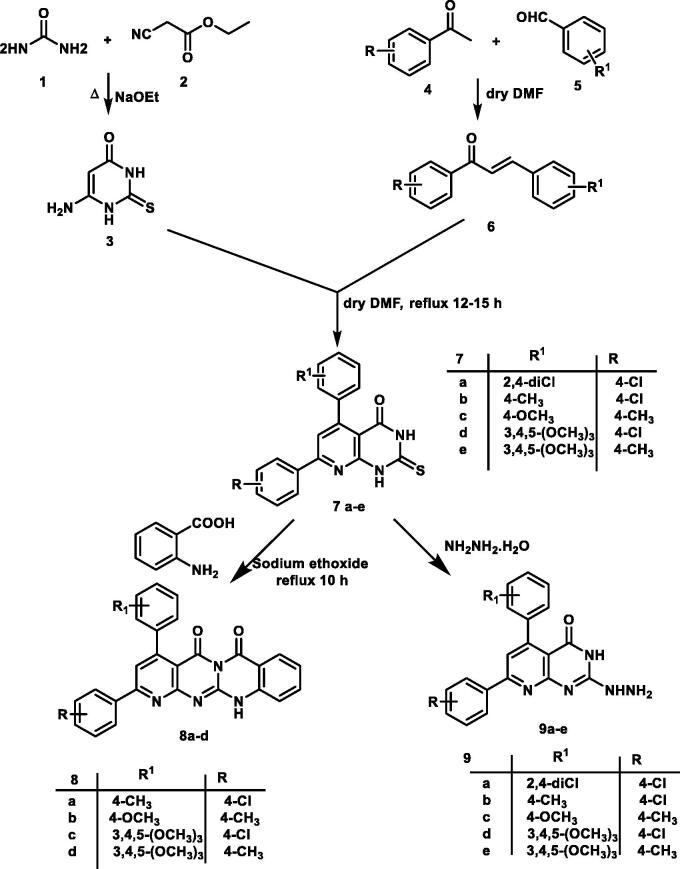
General procedure for the synthesis of the target compound **7a–e, 8a–d, a**nd **9a–e.**

**Scheme 2.**
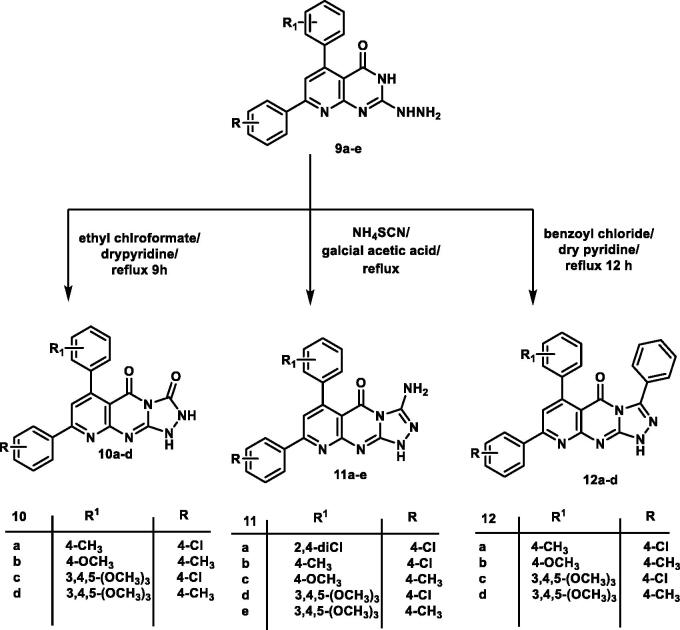
General procedure for the synthesis of the target compound **10a–d, 11a–e** and **12a–d.**

The 3-phenylpyrido[2,3-d][1,2,4]triazolo[4,3-a]pyrimidin-5(1H)-one analogues **12a–e** were obtained via the reaction of hydrazinyl derivatives **9a–e** with benzoyl chloride in pyridine under reflux conditions. Analytical and spectroscopic measurements confirmed the structures of compounds **12a–d**. The IR spectrum of **12b** displayed two absorption bands at 1720, 3414 cm^−1^ corresponds to C=O and NH groups, respectively. The ^1^H NMR spectrum of the same series gave an increase in aromatic integration due to the presence of an extra phenyl ring. The mass spectrum of **12b** revealed a molecular ion peak at *m/z* of 459 (Schemes 1 and 2).

### 2.2. Biological evaluation

#### 2.2.1. In vitro antiproliferative activities

All the final synthesised (19) compounds were tested for their anticancer activities against four tumour cell lines namely, lung cancer (A-549), prostate cancer (PC-3), colon cancer (HCT-116), and breast cancer (MCF-7) using standard MTT method^61–63^. Preliminary screening against the cancer cell lines was performed, using doxorubicin as a reference drug at doses of 100 μM. Variable results were recorded for the screened compounds as depicted in Table 1. The pyrido[2,3-d]pyrimidin-4(3H)-one derivatives (**8a, 8 b, 8d,** and **9a**) that exhibited inhibitory activity ≥70% were selected for IC_50_ screening comparing erlotinib.

**Table 1.** Percentage of growth inhibition activity of compounds **7a, 8a–d, 9a, 10a–e** against A549, PC-3, HCT-116 and MCF-7 at a concentration of 100 μM.

Comp.	Growth inhibition (%)			
	A549	PC-3	HCT-116	MCF-7
**7_a_**	14	12	18	34
**8_a_**	90	98	84	32
**8_b_**	94	96	67	9
**8_c_**	8	17	9	21
**8_d_**	97	95	78	15
**9_a_**	42	90	89	68
**10_a_**	17	29	21	41
**10_b_**	7	15	14	4
**10_c_**	9	52	23	8
**10_d_**	2	8	5	5
**11_a_**	2	30	9	7
**11_b_**	28	43	15	29
**11_c_**	26	28	3	23
**11_d_**	5	20	3	37
**11e**	30	22	6	23
**12_a_**	41	28	35	45
**12_b_**	33	36	56	54
**12_c_**	22	30	33	55
**12_d_**	4	14	5	15
Doxorubicin	100	100	100	100

All compounds were barely active against breast cancer (MCF-7) cell line at 100 μM (% of inhibition ranging from 5 to 68% (Table 1). By focussing on the prostatic cell line (PC-3), the anticancer profile of the tested compounds was significantly improved especially the tetracyclic derivatives **8a** (IC_50_ = 7.98 µM) and **8d** (IC_50_ = 7.12 µM) that exhibited about 1.5 times more active than erlotinib (11.05 µM). In addition, compound **9a** showed a strong activity against PC-3 line with an IC_50_ value of 9.26 µM. For compound **8d**, it showed a strong anti-proliferative activity against A-549 with an IC_50_ value of 7.23 µM which is comparable to erlotinib (IC_50_ = 6.53 µM). Compound **8b** revealed a moderate inhibitory activity against PC-3 cell line with an IC_50_ value of 18.01 µM. Generally, no cytotoxic activity was observed against the colon cancer cell line (HCT-116), but compounds **8a, 8b, 8d, 12b** revealed mild cytotoxic activity.

#### 2.2.2. Structural–activity relationship

**Figure 4.**
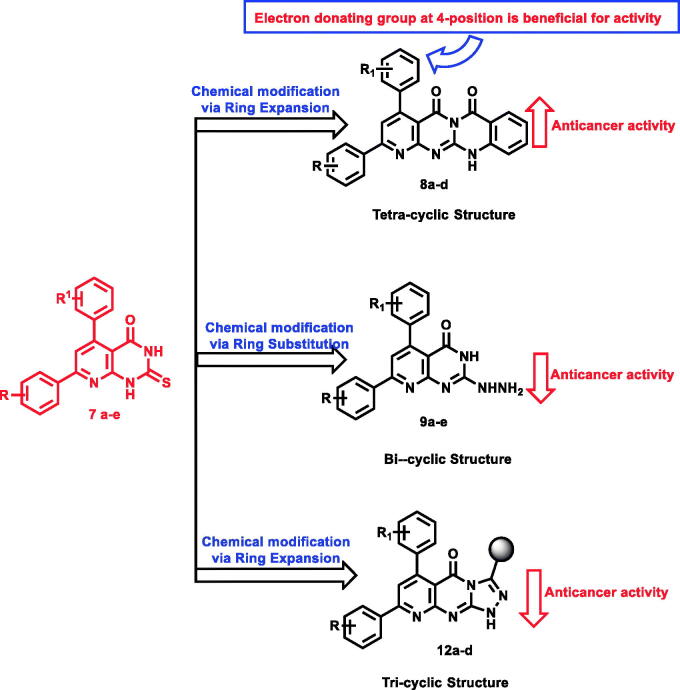
SAR according to modifiable moieties in the target compounds.

The synthetic pathway of the target compounds was depicted in two schemes starting with thioxo-precursors **7a–e** to afford tetracyclic derivatives **8a–d**, hydrazinyl derivatives 9a–e, and triazolyl derivatives **10a–d, 11a–e,** and **12a–d** (Figure 4).

Expansion of pyrido[2,3-d]pyrimidin-4(3H)-one core to give tetracyclic 5H-pyrido [2′,3′:4,5]pyrimido[2,1-b]quinazoline-5,7(12H)-dione derivatives **8a–d** showed the preferred impact on the evaluated anticancer activity. Compounds **8a, b, d** exhibited the most potent cytotoxic activity against A-549 cell line with IC_50_ values of 16.2, 16, and 7.23 μM, respectively, and the later was equipotent to erlotinib (IC_50_ = 6.53 μM).

Concerning prostate cancer cell line (PC-3), both compounds **8a** (IC_50_ = 7.98 μM), and **8d** (IC_50_ = 7.12 μM), were two-fold more potent than the reference molecule (IC_50_ = 11.05 μM). It was noticed that the electronic factor greatly influences the anticancer activity of the same series against lung and prostate cancer cells. For example, the existence of electron-donating (OCH_3_) group at 4-position of compounds **8a** and **8d** was beneficial for activity. The modification of tetracyclic derivatives **8a** (IC_50_ = 7.98 μM) into hydrazinyl derivatives **9a** (IC_50_ = 9.26 μM) decreased the anticancer activity against prostate cancer cell line (IC_50_ = 9.26 μM). In addition, the expansion of pyrido[2,3-d]pyrimidin-4(3H)-one scaffold into triazolyl analogues caused a remarkable drop in the activity with an inhibition range from 2 to 52% (Table 2).

**Table 2.** IC_50_ values of compounds 8_a_, 8_b_, 8_d_, 9_a_ and 12_b_ against A-549, PC-3, HCT-116 and MCF-7.

Compounds	IC_50_ (µM)^a^			
	A-549	PC-3	HCT-116	MCF-7
**8_a_**	16.2 ± 2.4	7.98 ± 2.4	25.61 ± 1.3	–
**8_b_**	10 ± 2.4	18.01 ± 2.3	26 ± 1.3	–
**8_d_**	**7.23 ± 2.1**	7.12 ± 2.0	70.17 ± 2	–
**9_a_**	–	9.26 ± 2.4	–	42 ± 1.2
**12_b_**	–	–	86.26 ± 2.2	–
Erlotinib	6.53 ± 0.82	11.05 ± 1.07	5.47 ± 0.3	4.21 ± 0.62

^a^All IC_50_ values are calculated as the mean of at least three different experiments.

#### 2.2.3. EGFR^WT^ kinase inhibitory assay

The promising antiproliferative compounds (**8a, 8d,** and **9a**) were further examined for their EGFR^WT^ kinase inhibitory activities using Homogeneous time resolved fluorescence (HTRF) assay^64^. Erlotinib was used as a reference molecule (Table 3).

**Table 3.** *In vitro* enzymatic inhibitory activities against EGFR^L858R^ and EGFR^790M^.

Comp.	EGFR^WT^ IC50 (µM)^a^	EGFR^T790M^ IC_50_ (µM)^a^
**8a**	0.099 ± 0.007	0.123 ± 0.010
**8d**	0.419 ± 0.029	0.290 ± 0.023
**9a**	0.594 ± 0.042	0.571 ± 0.046
Erlotinib	0.043 ± 0.003	0.071 ± 0.006

^a^The results were presented as Mean ± Standard error (SE) of three different tests.

The tested derivatives **8a, 8d,** and **9a** showed promising inhibitory activities against EGFR^WT^ with IC_50_ values of 0.099, 0.419, and 0.594 µM, respectively. compounds **8a** showed a good activity compared to erlotinib (IC_50_ = 0.043 µM). Whereas compounds **8d** and **9a** showed moderate act nlp0m kinase inhibitory assay

To evaluate the potential activity of the synthesised compounds against the mutant form of EGFR, the most active cytotoxic compounds (**8a, 8d** and **9a**) were tested for their inhibitory effect against EGFR^T790M^. Erlotinib was used as a positive control.

The tested compounds **8a, 8d** and **9a** showed inhibitory effects against EGFR^T790M^ with IC_50_ values of 0.123, 0.290, and 0.571 µM, respectively. Compound **8a** exhibited the highest inhibitory effect but less than erlotinib (IC_50_ = 0.071 µM). While compounds **8d** and **9a** showed moderate inhibitory activities (Table 3).

#### 2.2.4. Cell cycle analysis

Based on the above-mentioned biological testing, the most promising candidate **8a** was subjected to flow cytometry analysis to investigate its effect on the cell cycle distribution in the most sensitive cell line (PC-3). The reported protocol described by Wand et al.^65^ was applied in this test. PC-3 cells were incubated with compound **8a** for 24 h in a concentration equal to its IC_50_ against such cell line (7.98 µM). After that, the different phases of the cell cycle were analysed.

**Figure 5.**
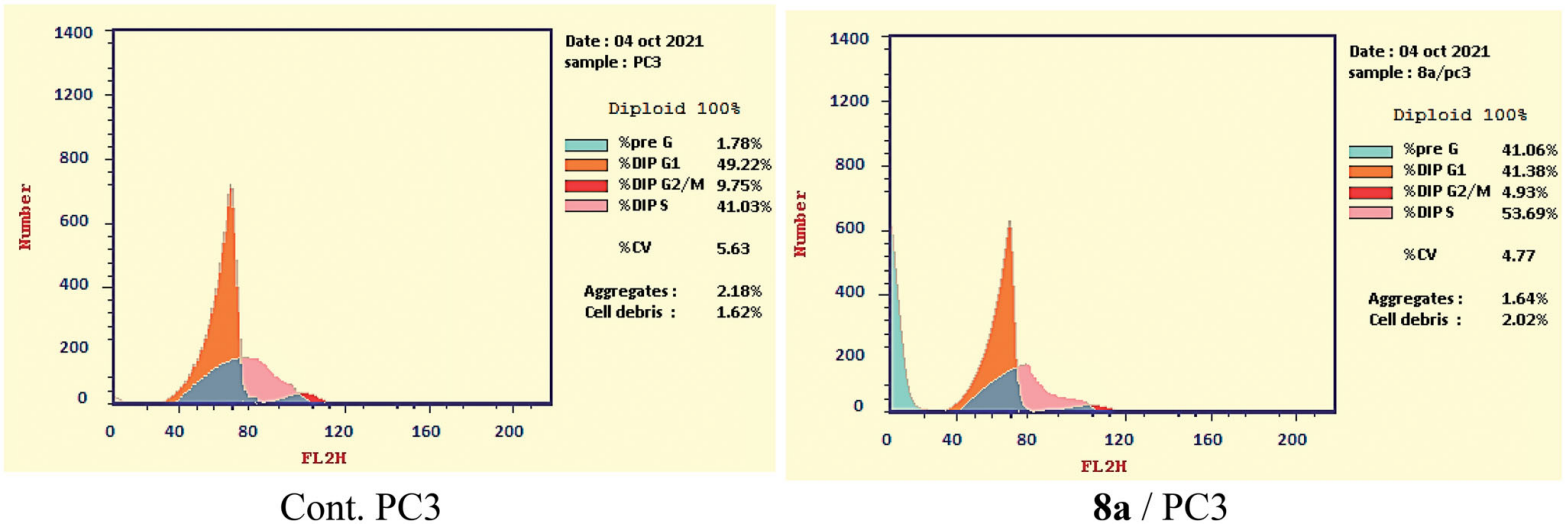
PC3distribution upon treatment with compound **8a**.

Compound **8a** showed different effects on the cell cycle distribution. Compared to the control cells (Cont. (PC-3)), the cell population increased at the phases of pre-G_1_ and %S by 22 and 1.3 folds, respectively. For the Pre-G1phase, the cell increased from 1.78% (in cont. cells) to 41.06% (at the treated cells). In the S phase, the cell increased from 41.03% (in cont. cells) to 53.69% (at the treated cells). On the other hand, the cell population decreased in both the G0–G1 and the G2-M phases. Such results obviously reveal that compound **8a** can arrest the PC-3 cell line at pre-G_1_ of the cell cycle (Figure 5 and Supplementary data).

#### 2.2.5. Annexin V-FITC apoptosis assay

**Figure 6.**
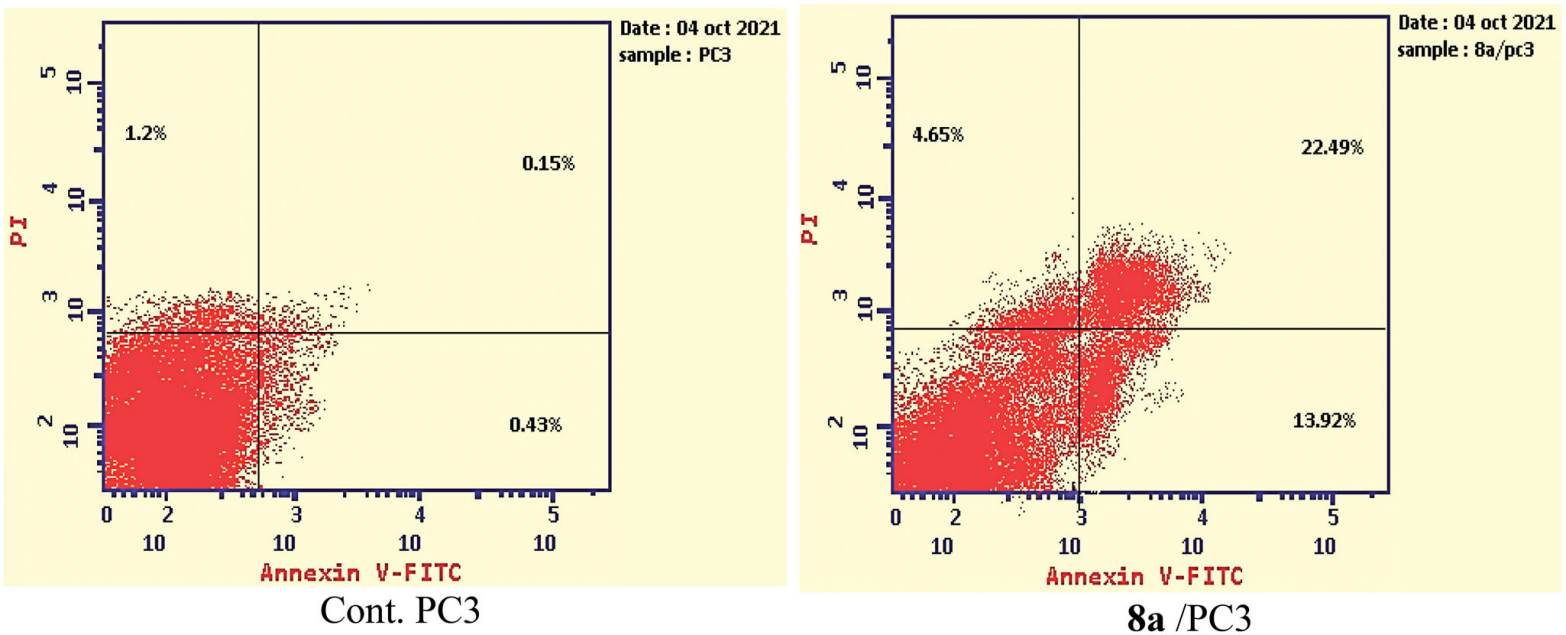
Apoptosis and necrosis percent induced by compound **8a**.

To analyse the apoptotic effect of the most active compound **8a**, Annexin V and PI double staining assay with FITC was applied^66^. In this test, PC-3 cells were incubated with compound **8a** at a concentration of 7.98 µM for 24 h. The results were depicted in (Figure 6 and Supplementary data).

Investigating the results of Annexin V and PI double staining assay, revealed that compound **8a** produced a significant increase in the early apoptosis ratio from 0.43 to 13.92% (32-fold). Also, it exerted an increase in the late apoptosis ratio from 0.15 to 22.49% (150-fold). Such findings indicate that compound **8a** has a significant apoptotic effect against PC-3 cells.

#### 2.2.6. Caspase-3 determination

The ability of a drug to induce apoptosis determines the sensitivity of the cancer cells against it. There are many signalling pathways that control apoptosis induction. Caspases family are considered as one of the most apoptotic regulators^67^. Activation of caspases especially (caspase-3) produces cell death^68^. In addition, it was reported that EGFR inhibitors exhibit significant apoptotic effects through the caspase pathway^69^^,^^70^. Here, the effect of the most active EGFR inhibitor **8a** on caspase-3 was examined in PC-3 cells. Compound **8a** was applied on PC-3 cells at a concentration of 3.04 µM for 24 h. The results revealed that such a compound generated a marked increase in the level of caspase-3 (452.3 pg/mL, 5.3-fold) compared to the control cells (84.24 pg/mL). In addition, the tested compound showed a comparable effect with the reference compound; staurosporine (413.1 pg/mL; Table 4 and Supplementary data).

**Table 4.** Effect of compound 8a on active caspase-3 in PC-3 cells after 24 h treatment.

Sample	Caspase-3 (pg/mL)^a^
**8a**/PC-3	452.3 ± 10.5
Staurosporine/PC-3	413.1 ± 11.66
Cont. (PC-3)	84.24 ± 16.5

^a^Values are given as mean ± SEM of three independent experiments.

### 2.3. Docking studies

To confirm our rationale of design, the binding modes of the synthesised compounds were investigated against the proposed targets using a docking approach. The used biological targets in docking studies were EGFR-TK Wild-type (EGFR^WT^, PDB: 4HJO)^71^ and EGFR-TK mutant type (EGFR^T790M^, PDB: 3W2O)^72^ using MOE 14.0 software. The co-crystallised ligands were used as reference molecules. The output of docking studies showed a high affinity of the synthesised compounds against the two tested targets compared to the reference molecules (Table 5).

**Table 5.** The docking binding free energies of the synthesised compounds against EGFR^WT^ and EGFR^T790M^.

Comp.	Binding free energy (kcal/mol)	
	EGFR^WT^	EGFR^T790M^
**7a**	–16.16	–11.59
**7b**	–16.61	–11.77
**7c**	–19.17	–15.03
**7d**	–21.41	–16.26
**7e**	–22.42	–19.57
**8a**	–19.29	–15.63
**8b**	–19.06	–18.13
**8c**	–21.92	–19.40
**8d**	–21.92	–19.30
**9a**	–15.80	–12.15
**9b**	–16.62	–15.14
**9c**	–19.23	–15.18
**9d**	–21.55	–17.63
**9e**	–22.46	–17.63
**10a**	–17.13	–15.27
**10b**	–19.19	–15.20
**10c**	–21.28	–16.57
**10d**	–22.48	–19.86
**11a**	–17.02	––14.22
**11b**	–16.92	–15.58
**11c**	–19.28	–15.89
**11d**	–21.60	–17.33
**11e**	–22.32	–19.22
**12a**	–20.96	–18.50
**12b**	–21.02	–20.62
**12c**	–21.67	–21.70
**12d**	–23.46	–22.39
Erlotinib	–22.12	–
TAK-285	–	–18.70

**Figure 7.**
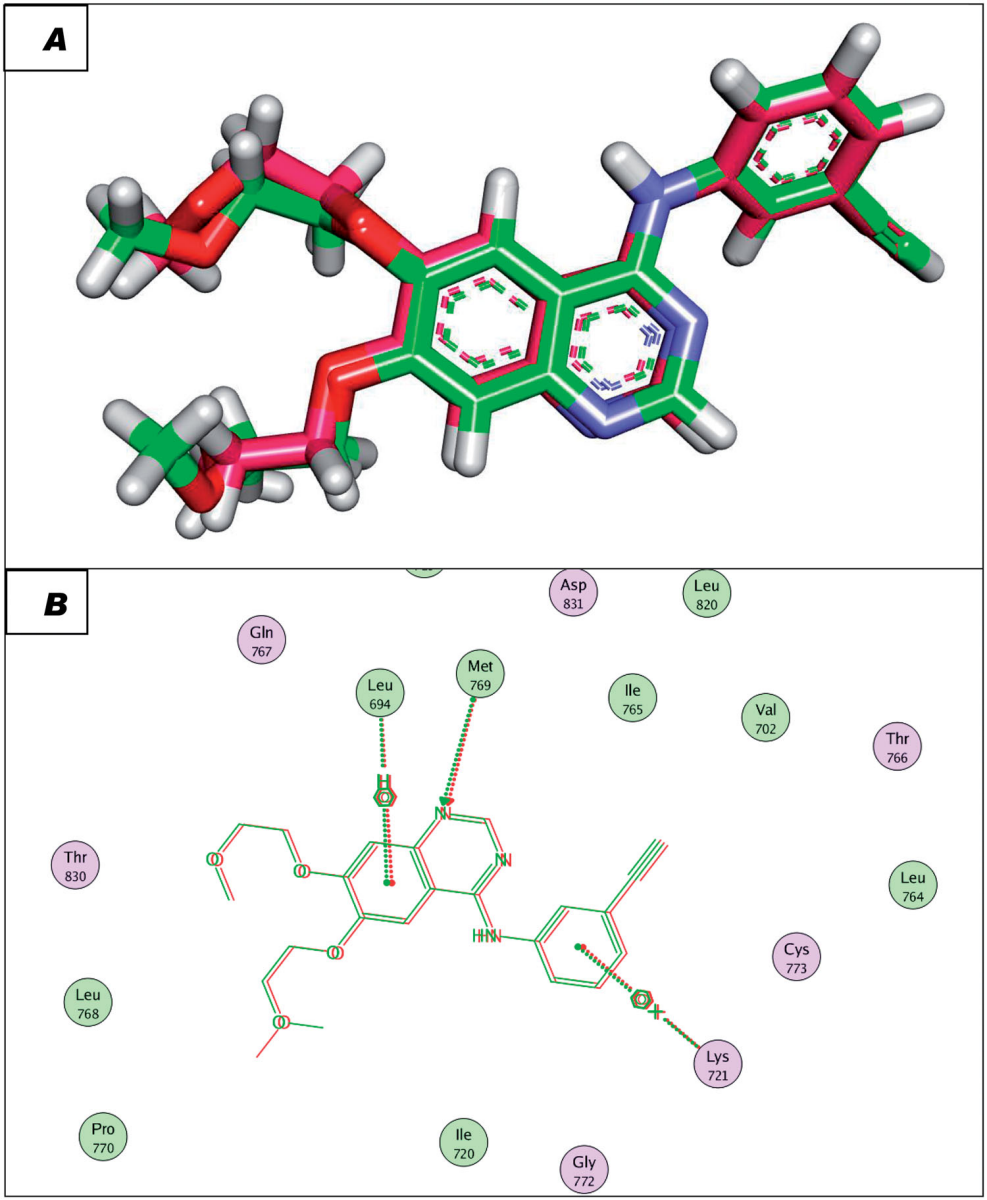
(A and B) 3D and 2D superimposition of the docked ligand (erlotinib; pink) and the original ligand (green) with RMSD value of 0.88 Å.

**Figure 8.**
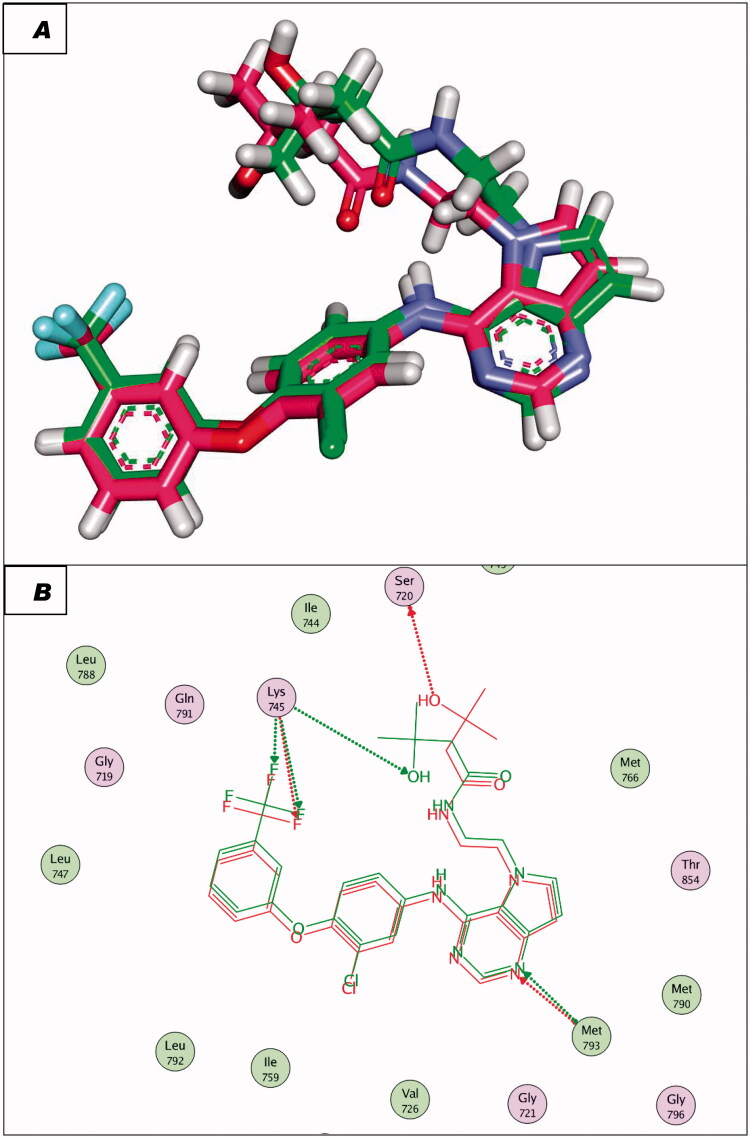
(A and B) 3D and 2D superimposition of the docked ligand of mutant EGFR (TAK-285; Pink) and the original ligand (green) with RMSD value of 1.06 Å.

To validate the docking procedures, the co-crystallised ligands (Erlotinib and TAK-285) were re-docked against EGFR^WT^ and EGFR^T790M^, respectively. The RMSD of docked and original ligands of erlotinib and TAK-285 were 0.88 and 1.05 Å, respectively. These values indicate the validity of the docking protocol (Figures 7 and 8).

**Figure 9.**
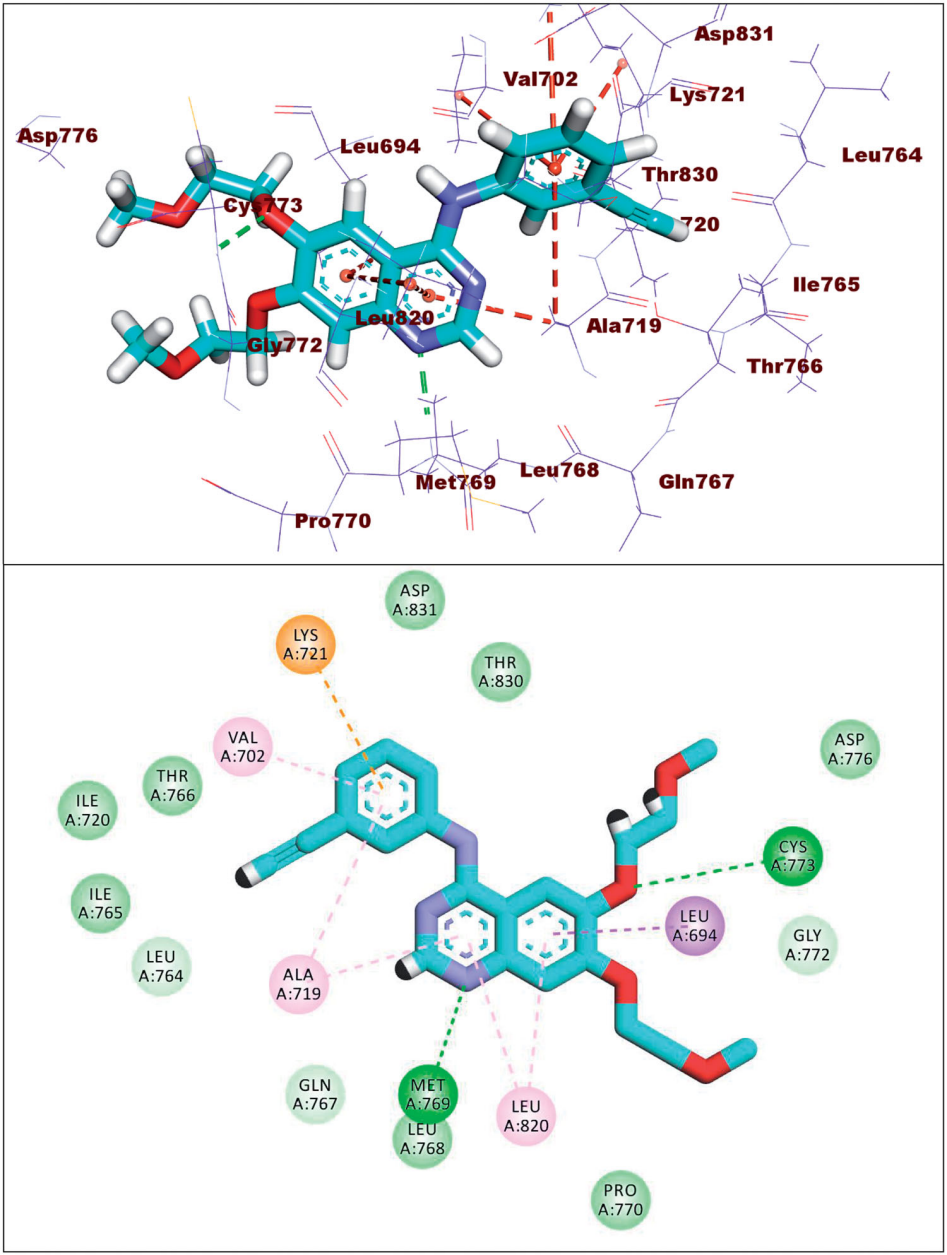
Erlotinib docked into the active site of EGFR^WT^.

The co-crystallised ligand (erlotinib) of EGFR^WT^ showed a binding energy of −22.12 kcal/mol. The heterocyclic system (quinazoline moiety) was buried in the adenine pocket forming a hydrogen bond with Met769. Also, it formed four hydrophobic interactions with Lue694, Ala719, and Leu820. The ethynylphenyl moiety was oriented into the hydrophobic pocket I forming three hydrophobic interactions with Ala719, Val702, and Lys721. The 2-methoxyethoxy groups occupied the hydrophobic region II forming a hydrogen bond with Cys773 (Figure 9).

**Figure 10.**
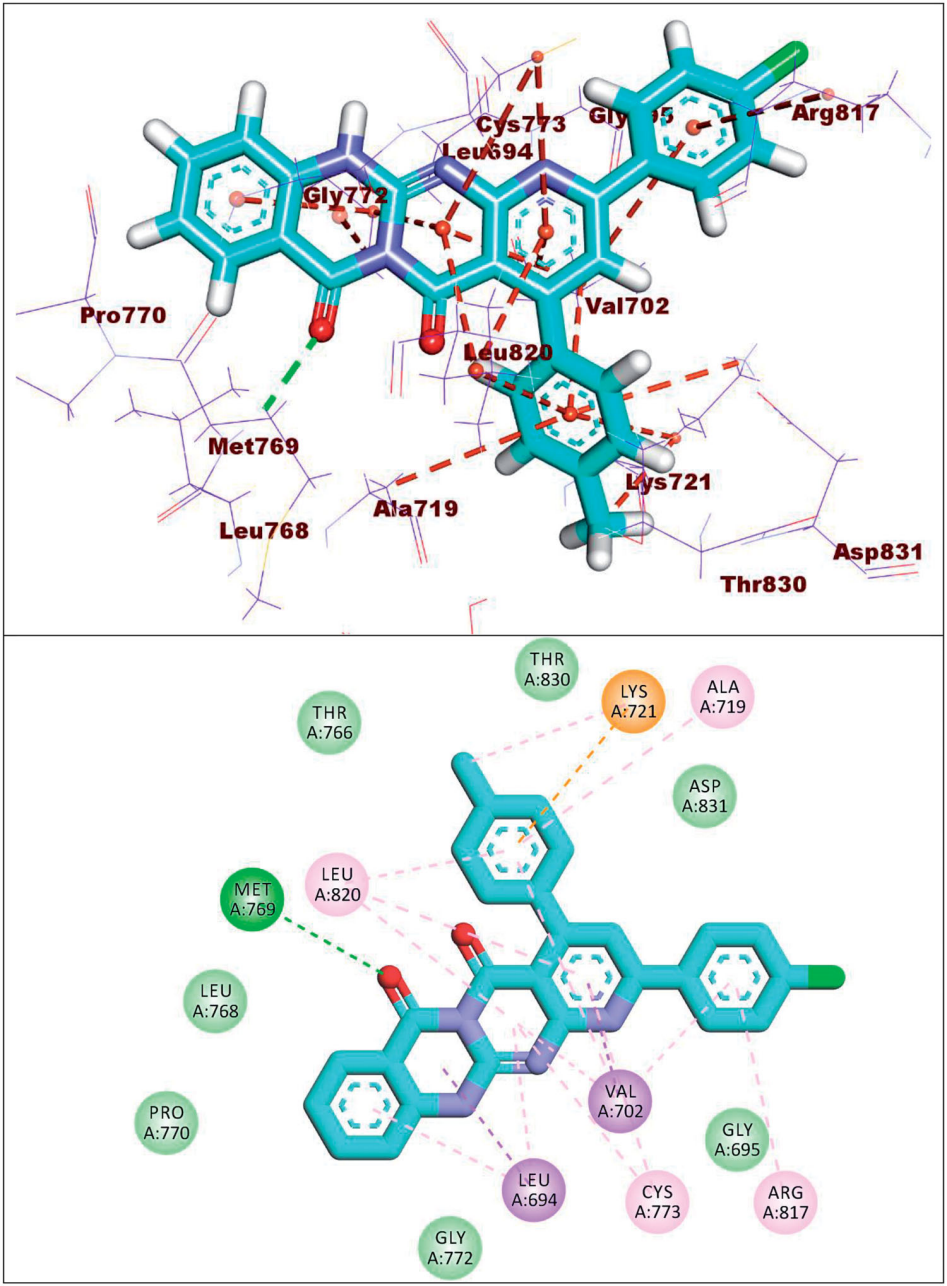
Compound **8a** docked into the active site of EGFR^WT^.

Compound **8a** showed a binding mode like that of erlotinib with a binding energy of −19.29 kcal/mol. The 5H-pyrido[2′,3′:4,5]pyrimido[2,1-b]quinazoline-5,7(12H)-dione moiety occupied the adenine pocket of the EGFR^WT^ forming one hydrogen bond with the crucial amino acid Met769. In addition, it formed nine hydrophobic interactions with Val702, Leu694, and Leu820. The tolyl moiety occupied the hydrophobic pocket I forming four hydrophobic interactions with Leu890, Ala719, and Lys721. Moreover, the 4-chlorophenyl moiety occupied the hydrophobic region II forming two hydrophobic interactions with Val702 and Arg817 (Figure 10).

**Figure 11.**
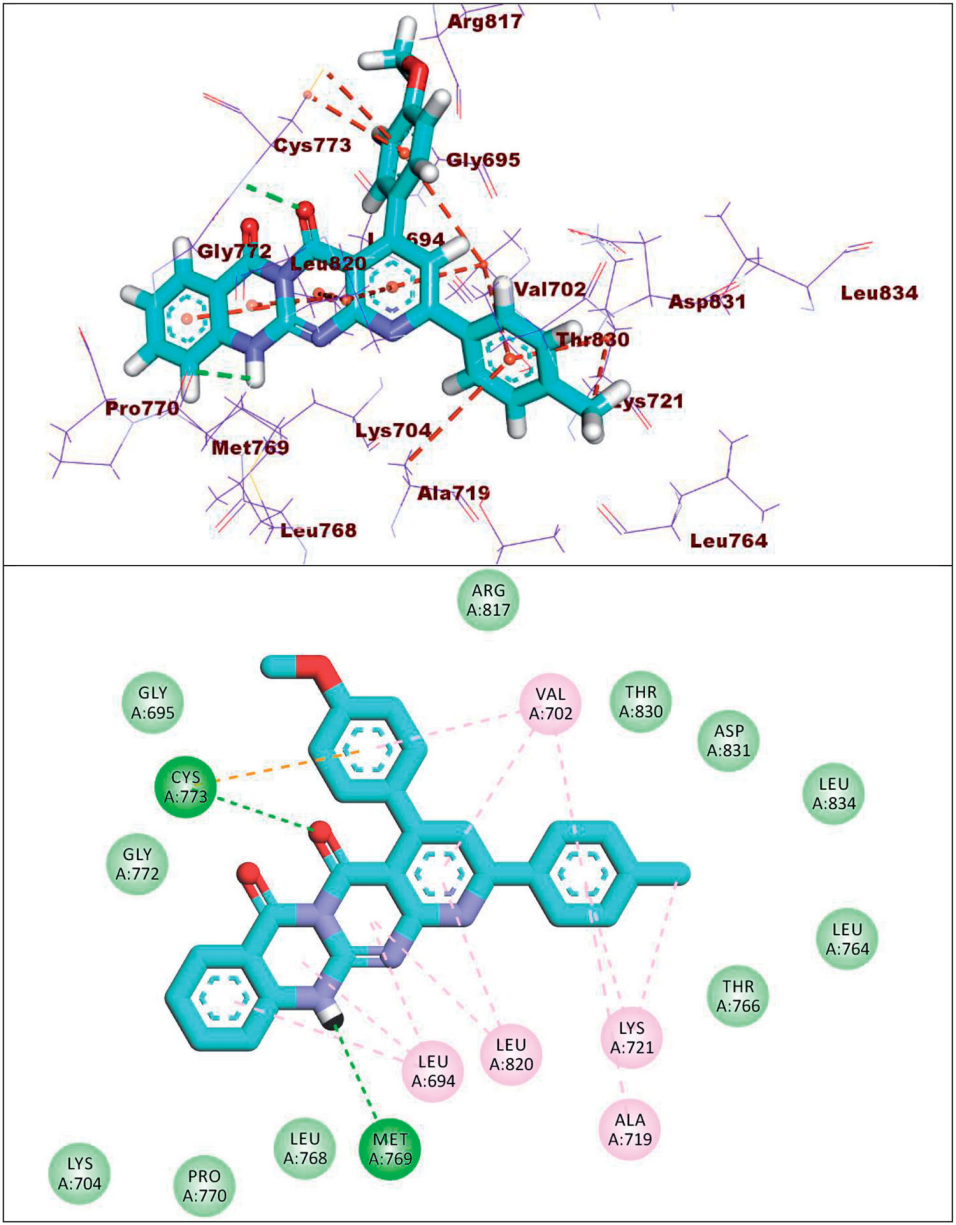
Compound **8b** docked into the active site of EGFR^WT^.

Compound **8b** showed a binding energy of −19.06 kcal/mol. The 5H-pyrido[2′,3′:4,5]pyrimido[2,1-b]quinazoline-5,7(12H)-dione moiety was buried in the adenine pocket forming two hydrogen bonds with acid Met769 and Cys773. In addition, it formed six hydrophobic interactions with Val702, Leu694, and Leu820. The 4-methoxyphenyl moiety occupied pocket I forming two hydrophobic interactions with val702, and Cys773. Moreover, the 4-chlorophenyl moiety occupied the hydrophobic II forming three hydrophobic interactions with Val721 and Ala719 (Figure 11).

**Figure 12.**
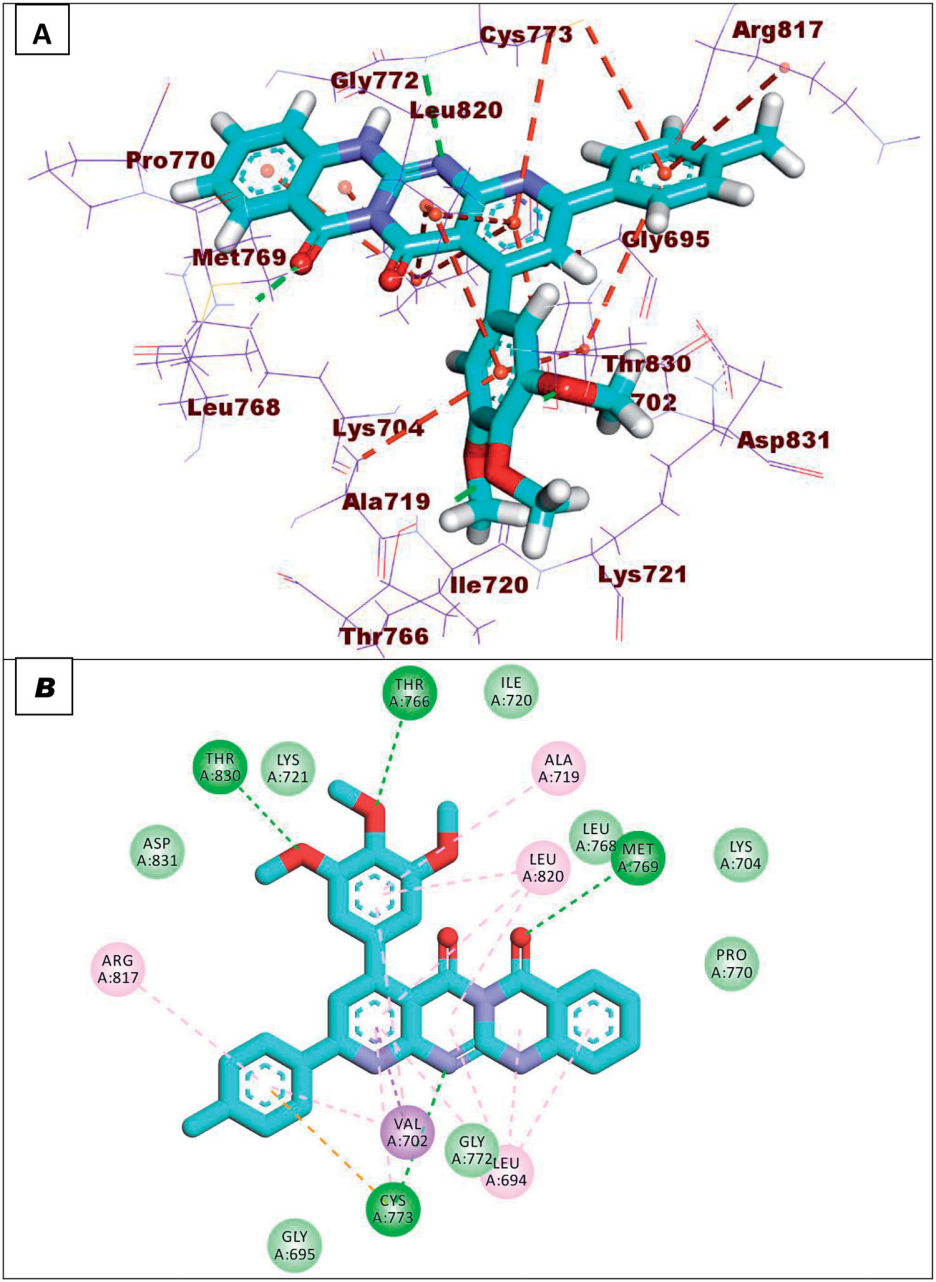
Compound **8d** docked into the active site of EGFR^WT^.

With regard to compound **8d**, it showed a binding mode similar to the refrence molecules with a binding energy of −21.92 kcal/mol. The 5H-pyrido[2′,3′:4,5]pyrimido[2,1-b]quinazoline-5,7(12H)-dione moiety was involved in two hydrogen bonds with the amino acids Met769 and Cys773 in the adenine pocket. In addition, it formed eight hydrophobic interactions with Val702, Leu694, Gly772, and Leu820. The 3,4,5-trimethoxyphenyl moiety occupied pocket I forming two hydrophobic interactions with Ala719 and Leu820. It formed two hydrogen bonds with Thr766 and Thr830. Moreover, the tolyl moiety occupied the hydrophobic II forming three hydrophobic interactions with Val702, Arg817 and Cys773 (Figure 12).

**Figure 13.**
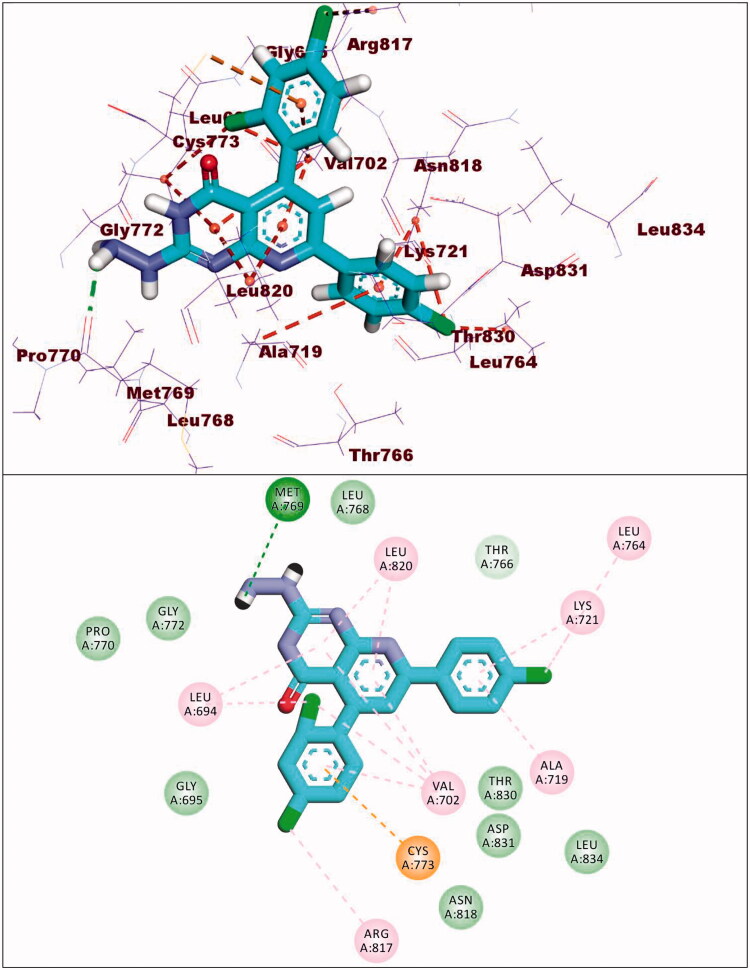
Compound **9a** docked into the active site of EGFR^WT^.

Compound **9a** showed a binding energy of −15.80 kcal/mol. The 2-hydrazinylpyrido[2,3-d]pyrimidin-4(3H)-one moiety was inserted in the adenine pocket forming a hydrogen bond with the amino acid Met769. Further, it formed five hydrophobic interactions with Val702, Leu694, and Leu820. The 4-chlorophenyl moiety occupied pocket I forming three hydrophobic interactions with Leu764, Lys721, and Ala719. Moreover, the 2,4-dichlorophenyl moiety occupied the hydrophobic II forming five hydrophobic interactions with Val702, Arg817, Leu694, and Cys773 (Figure 13).

**Figure 14.**
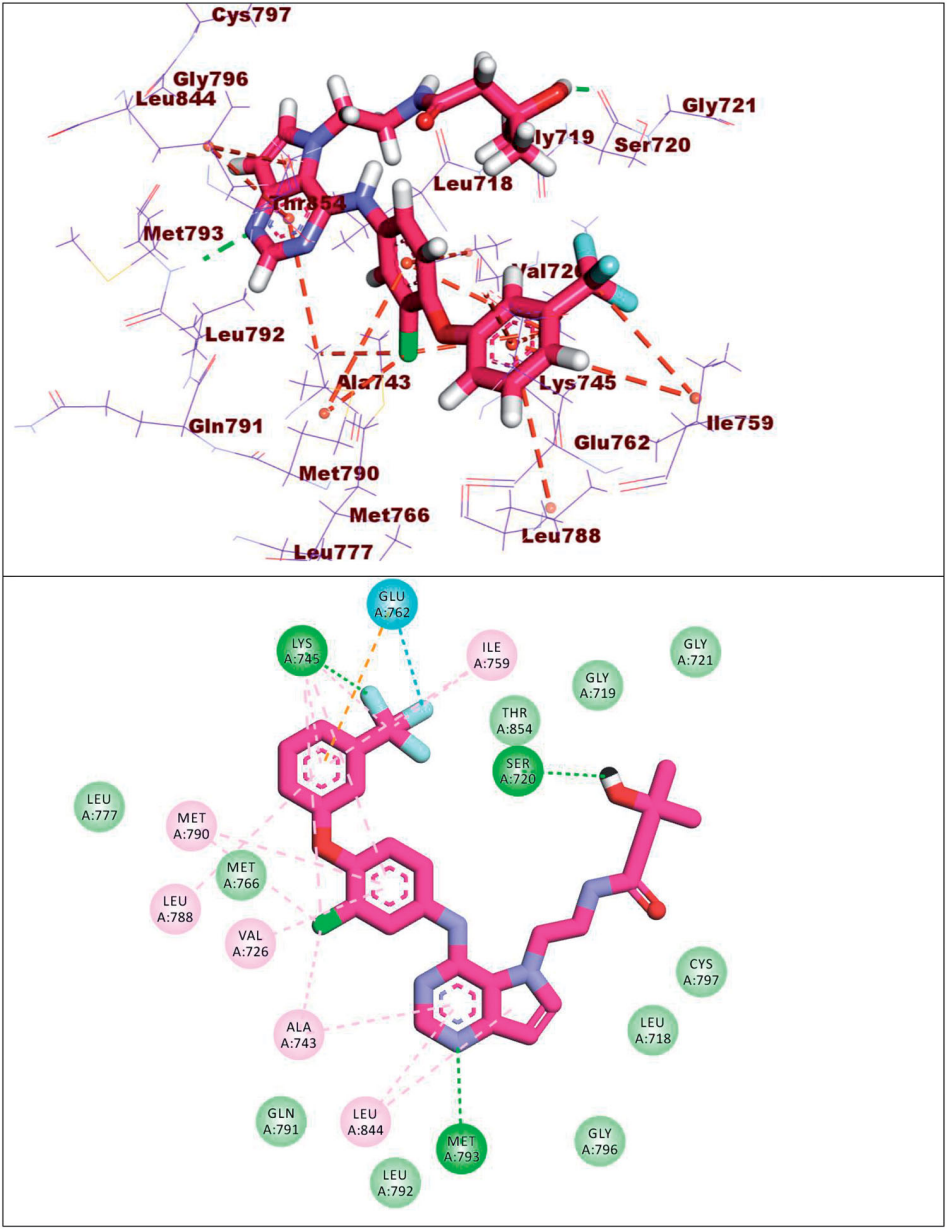
Co-crystallised ligand (TAK-285) docked into the active site of EGFR^T790M^.

The synthesised compounds showed good binding affinities against EGFR^T790M^ with binding free energies ranging from −11.59 to −22.39 kcal/mol (Table 5). The co-crystallised ligand (TAK-285) exhibited a binding energy of −18.70 kcal/mol. The pyrrolo[3,2-d]pyrimidine moiety was buried in the adenine pocket forming a hydrogen bond with Met793 and three hydrophobic bonds with Leu844 and Ala743. The terminal 3-(trifluoromethyl)phenoxy group occupied the hydrophobic pocket I forming a hydrogen bond with Lys745. Also, it formed seven hydrophobic interactions with Lys745, Glu762, Leu788, and Ile759. In addition, the N-ethyl-3-hydroxy-3-methylbutanamide moiety occupied the hydrophobic region II forming hydrogen bond with Ser720. The phenyl moiety formed hydrophobic interactions with Met790, Val726, and Ala743 (Figure 14).

**Figure 15.**
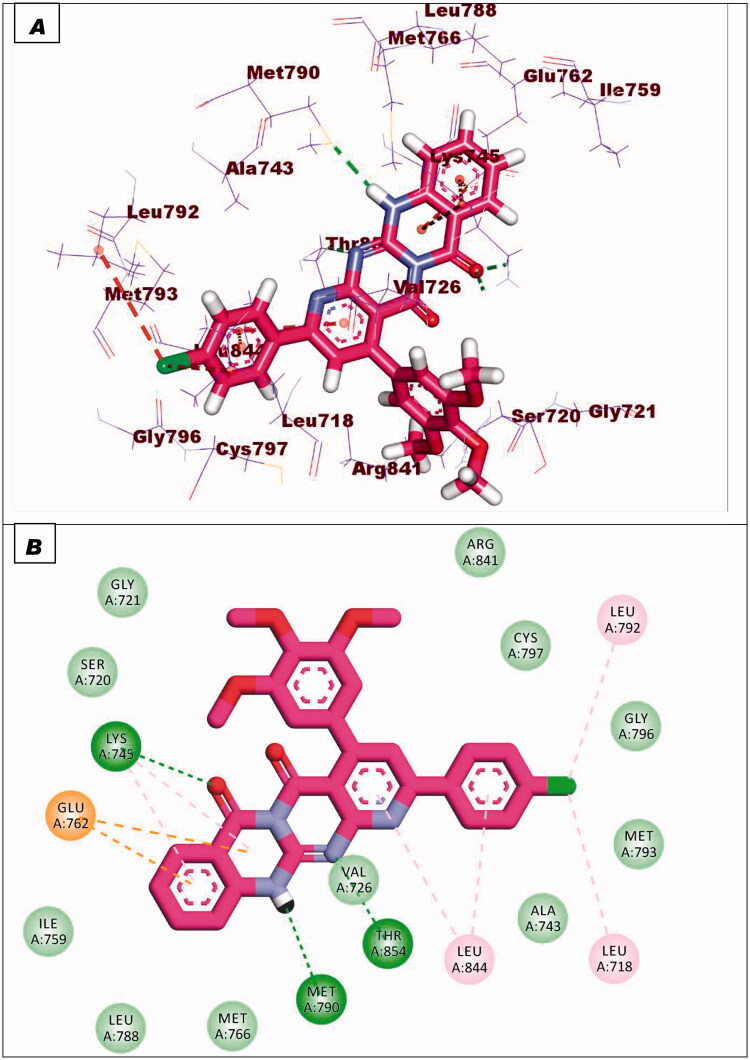
Binding of compound **8c** with EGFR^T790M^.

Compound **8c** exhibited a binding mode similar to that of TAK-285 with an affinity value of −19.40 kcal/mol. The 5H-pyrido[2′,3′:4,5]pyrimido[2,1-b]quinazoline-5,7(12H)-dione moiety occupied the adenine pocket of forming five hydrophobic interaction with Lys745, Glu762, and Leu844. Also, it formed three hydrogen bonds with Thr854, Met790, and Lys745. The 3,4,5-trimethoxyphenyl moiety occupied the hydrophobic pocket I and 4-chloro phenyl moiety occupied the hydrophobic region II forming three hydrophobic bonds with Leu718, Leu844, and Leu792 (Figure 15).

**Figure 16.**
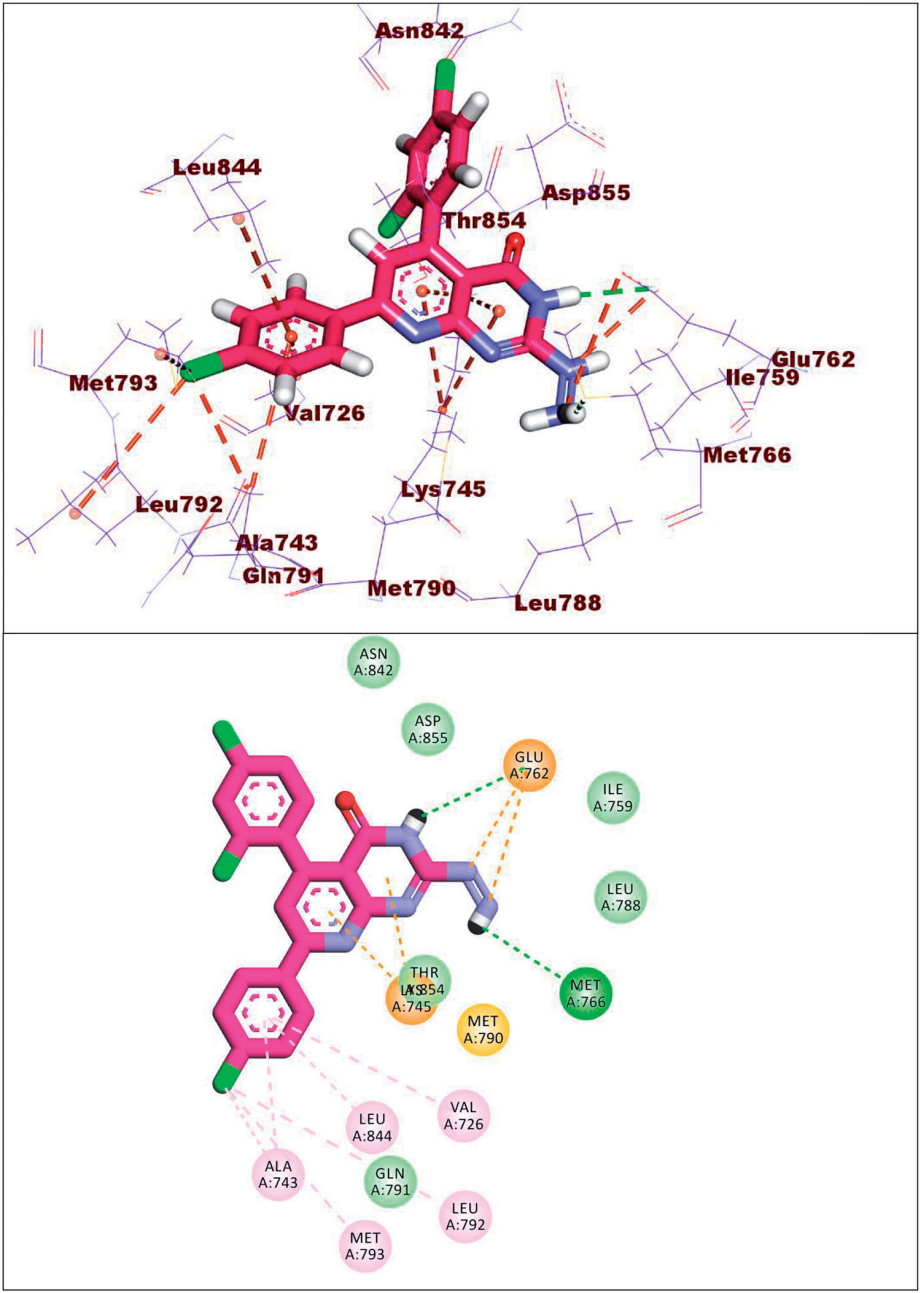
Binding of compound **9a** with EGFR^T790M^.

Compound **9a** exhibited a binding energy of −12.15 kcal/mol. The 2-hydrazinylpyrido[2,3-d]pyrimidin-4(3H)-one moiety occupied the adenine pocket of forming four hydrophobic interaction with Lys745,Glu762, and Thr854. Also, it formed two hydrogen bonds with Glu762 and Mey766. The 2,4-dichlorophenyl moiety occupied the hydrophobic pocket I and 4-chloro phenyl moiety occupied the hydrophobic region II forming six hydrophobic bonds with Val726, Met793, Ala743, Leu844 and Leu792 (Figure 16).

**Figure 17.**
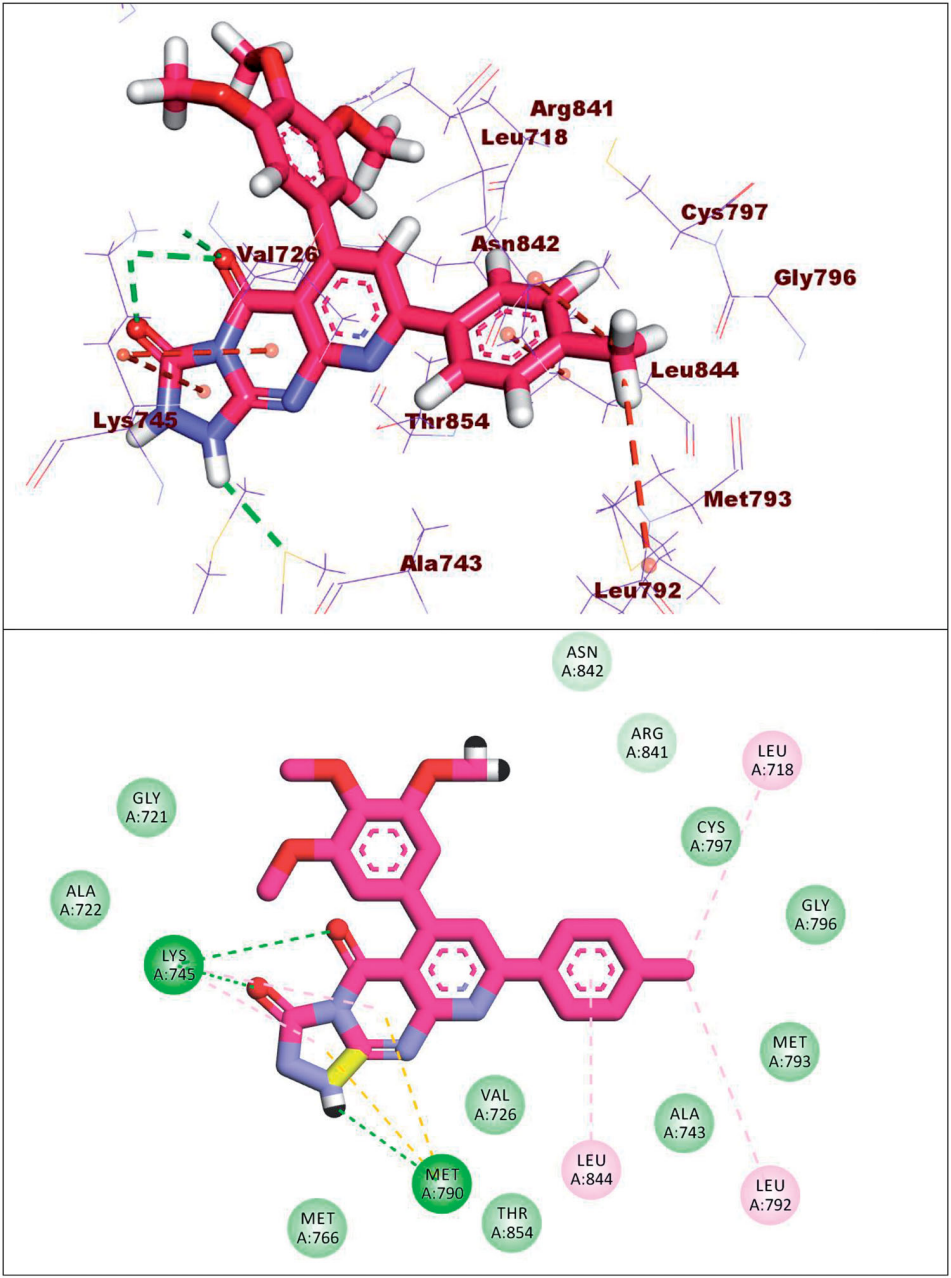
Binding of compound **10d** with EGFR^T790M^.

Compound **10d** exhibited a binding energy of −19.86 kcal/mol. The 1,2-dihydropyrido[2,3-d][1,2,4]triazolo[4,3-a]pyrimidine-3,5-dione moiety occupied the adenine pocket of forming three hydrogen bonds with Lys745, and Met790. Also, it formed four hydrophobic interactions with Lys745, and Met790. The 3,4,5-trimethoxyphenyl moiety occupied the hydrophobic pocket I and tolyl moiety occupied the hydrophobic region II forming three hydrophobic bonds with Leu718, Leu844 and Leu792 (Figure 17).

**Figure 18.**
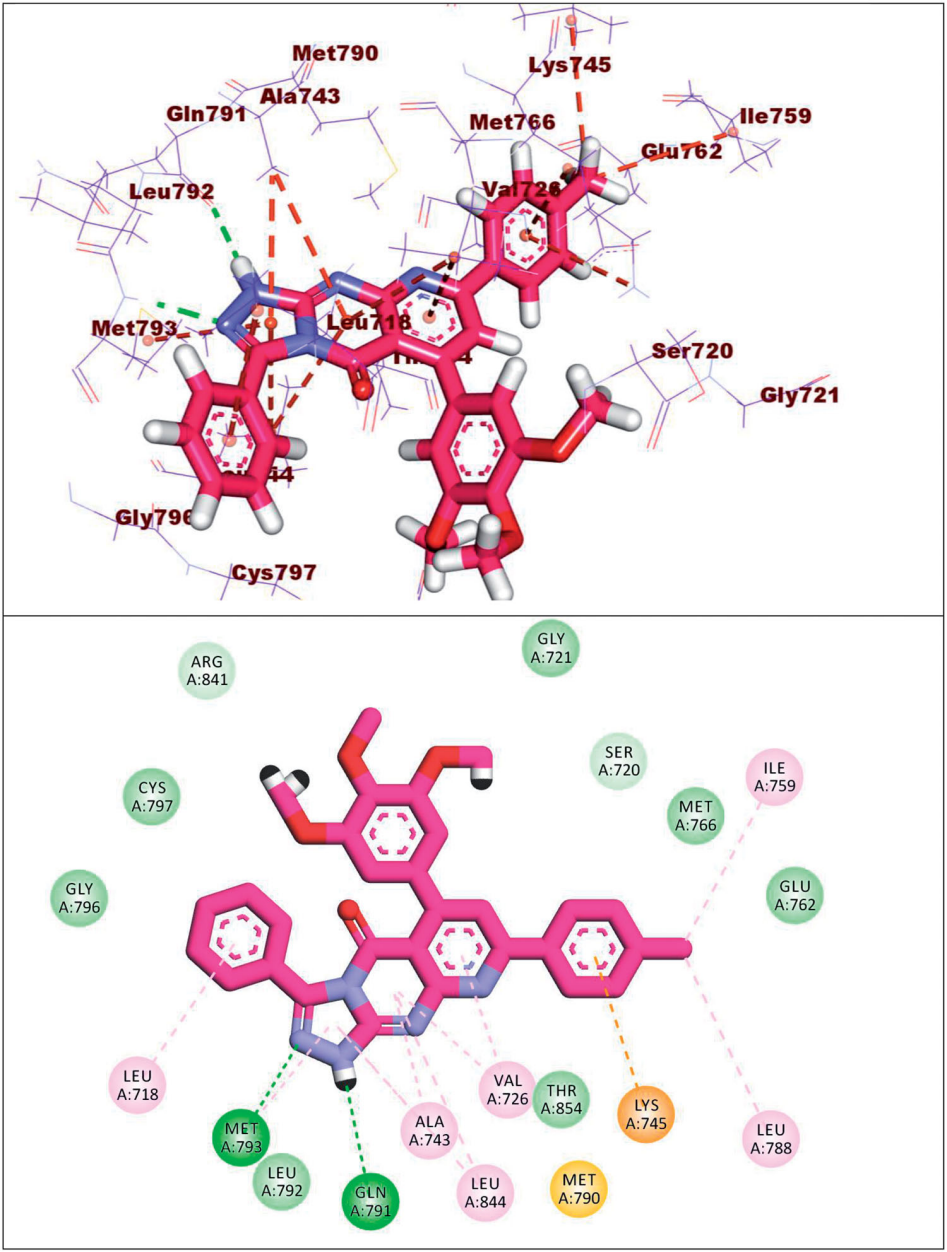
Binding of compound **12d** with EGFR^T790M^.

Compound **12d** exhibited a binding energy of −22.39 kcal/mol. The pyrido[2,3-d][1,2,4]triazolo[4,3-a]pyrimidin-5(1H)-one moiety occupied the adenine pocket of forming two hydrogen bonds with Gln791 and Met793. Also, it formed six hydrophobic interactions withVal726, Leu844, Ala743, and Met793. The 3,4,5-trimethoxyphenyl moiety occupied the hydrophobic pocket I and tolyl moiety occupied the hydrophobic region II forming three hydrophobic bonds with Leu788, Ile759 and Lys745 (Figure 18).

## 3. Conclusion

New nineteen pyrido[2,3-d]pyrimidin-4(3H)-one derivatives have been designed and synthesised as EGFR inhibitors. These compounds were evaluated for antiproliferative activities against A-549, PC-3, HCT-116, and MCF-7 cell lines. Compounds **8a, 8 b, 8d, 9a,** and **12b** exhibited the highest activities. Compound **8a** showed promising activities against A-549, PC-3, and HCT-116 cell lines with IC_50_ values of 16.2, 7.98, and 25.61 µM, respectively. Compounds **8a, 8b**, and **9a** showed promising inhibitory activities against EGFR^WT^ with IC_50_ values of 0.099, 0.419, and 0.594 µM, respectively. In addition, such derivatives showed good inhibitory effects against EGFR^T790M^ with IC_50_ values of 0.123, 0.290, and 0.571 µM, respectively. The most promising candidate **8a** induced a significant apoptotic effect in PC-3 cells and arrested the cell cycle at the pre-G1 phase. Structure-activity relationship studies revealed that tetracyclic 5H-pyrido [2′,3′:4,5]pyrimido[2,1-b]quinazoline-5,7(12H)-dione derivatives **8a–d** have the preferred impact on the anticancer activity. In addition, the existence of an electron-donating (OCH_3_) group at 4-position of compounds **8a** and **8d** is beneficial for activity. To give an additional comprehensive investigation about the mechanism of action of the synthesised compounds, docking studies were performed against EGFR^WT^ and EGFR^T790M^. Docking studies revealed that the synthesised compounds have similar binding modes against the prospective biological targets. This work introduces compounds **8a** as a potential promising EGFR inhibitor.

## 4. Experimental

### 4.1. Chemistry

#### 4.1.1. General

All details of chemicals and different apparatus for analyses were provided in Supplementary data.

#### 4.1.2. General procedure for synthesis of thioxopyridopyrimidinone 7a–e

A mixture of the appropriate α, β-unsaturated ketones **6a–e** (0.01 mol) and 6-amino-2,3-dihydro-2-thioxopyrimidin-4(1H)-one (3) (1.43 g, 0.01 mol) was heated in dry DMF (20 ml) under reflux for 10–15 h. After cooling, the precipitates were filtered and crystallised from DMF to afford compounds **7a–e**. All spectral data of thioxo derivatives **7b–e** was reported in our previous work^73^. Herein, we described our newly synthesised thioxo precursor **7a.**

##### 4.1.2.1. 7–(4-Chlorophenyl)-5–(2,4-dichlorophenyl)-2-thioxo-2,3-dihydropyrido [2,3-d]pyrimidin-4(1H)-one (7a)

Yield (50%); m.p. 318–320 °C. IR (KBr) (cm^−1^): 3387 (NH), 1701 (C=O); ^1^HNMR (400 MHz, DMSO-d_6_) *δ* (ppm): 7.40–7.76(m, 5H, Ar-H), 7.97 (s, 1H, pyridine-H6), 8.26 (d, *J* = 8 Hz, 2H, chlorophenyl-H2,H6), 12.50 (brs, 1H, NH, D_2_O exchangeable); 13.23 (brs, 1H, NH, D_2_O exchangeable); ^13^CNMR (DMSO-d_6_) *δ* (ppm): 108.8, 118.5, 126.5, 127.7, 128.7, 129.1, 130.1, 132.4, 133.1, 134.7, 136.7, 149.3, 152.4, 158.3, 158.6, 162.2, 175.6; MS (*m/z*) 434; Anal. Calc. for: (C_19_H_10_Cl_3_N_3_OS): C, 52.50; H, 2.32; N, 9.67; Found: C, 52.57; H, 2.36; N, 9.73%.

#### 4.1.3. General procedure for synthesis of 2,4-diaryl-5Hpyrido [2',3':4,5] pyrimido[2,1-b]quinazoline-5,7(12H)-dione(8a–d)

A mixture of 2-thioxopyrido[2,3-d]pyrimidine derivatives **7a–e** (0.01 mol) and anthranilic acid (1.37 g, 0.01 mol) was heated under reflux for 20 h in the presence of 2% sodium ethoxide (20 ml) The reaction mixture was cooled, poured into ice cold water and acidified by diluted hydrochloric acid. The formed precipitate was filtered, washed several times with water, dried and washed with hot ethanol to give the compounds **8a–d**.

##### 4.1.3.1. 2–(4-Chlorophenyl)-4-(p-tolyl)-5H-pyrido[2',3':4,5]pyrimido [2,1-b] quinazoline-5,7(12H)-dione (8a)

Yield (68%); m.p. >300 °C. IR(KBr) (cm^−1^): 3479 (NH), 1750 (C=O), 1685 (C=O); ^1^HNMR (400 MHz, DMSO-d_6_) *δ* (ppm): 2.36 (s, 3H, CH_3_), 6.45 (m, 1H, Ar-H), 6.66 (d, *J* = 8.4 Hz, 2H, Ar-H), 7.10 (t, *J* = 8 Hz, 1H, Ar-H), 7.20 (d, *J =* 8 Hz, 2H, Ar-H), 7.31(d, J = 8 Hz, 2H, Ar-H), 7.50 (s, 1H, C6-pyridine), 7.56 (d, *J* = 8.4 Hz, 2H, Ar-H), 8.42 (d, *J* = 8.4 Hz, 2H, Ar-H), 11.19 (brs, 1H, NH, D_2_O exchangeable); ^13^CNMR (DMSO-d_6_) *δ* (ppm): 21.3, 106.3, 114.5, 116.1, 118.4, 127.7, 128.6, 129.0, 129.3, 129.36, 129.6, 131.8, 132.6, 135.5, 136.0, 136.2, 137.4, 150.1, 153.6, 154.2, 157.9, 169.7, 169.8; MS (*m/z*): 466 (M + 2), 464 (M^+^); Anal. Calc. for: (C_27_H_17_ClN_4_O_2_): C, 69.75; H, 3.69; N, 12.05; % Found: C, 69.82; H, 3.74; N, 12.11%.

##### 4.1.3.2. 4–(4-Methoxyphenyl)-2-(p-tolyl)-5H-pyrido[2',3':4,5] pyrimido[2,1-b] quinazoline-5,7(12H)-dione (8b)

Yield (52%); m.p. >300 °C. IR (KBr) (cm^−1^): 3410 (NH), 1693 (C=O), 1750 (C=O); ^1^HNMR (400 MHz, DMSO-d_6_) *δ* (ppm): 2.36 (s, 3H, CH_3_), 3.79 (s, 3H, OCH_3_), 6.92–6.95 (m, 2H, Ar-H), 7.29–7.44 (m, 6H, Ar-H), 7.49 (s, 1H, C-6 pyridine), 8.07–8.10 (m, 4H, Ar-H), 11.11 (brs, 1H, NH, D_2_O exchangeable); ^13^CNMR (DMSO-d_6_) *δ* (ppm): 21.3, 55.6, 106.3, 114.5, 116.2, 118.7, 127.3, 128.1, 129.3, 129.3, 129.6, 131.4, 132.2, 135.4, 136.8, 136.2, 137.8, 150.2, 153.7, 154.8, 154.5, 157.6, 169.7, 169.8; MS (*m/z*): 460; Anal. Calc, for: (C_28_H_20_N_4_O_3_); C, 73.03; H, 4.38; N, 12.17%; Found: C, 73.07; H, 4.44; N, 12.23%.

##### 4.1.3.3. 2–(4-Chlorophenyl)-4–(3,4,5-trimethoxyphenyl)-5H-pyrido [2',3':4,5] pyrimido[2,1-b]quinazoline-5,7(12H)-dione (8c)

Yield (87%); m. p. <300 °C. IR (KBr) (cm^−1^): 3468 (NH), 1708 (C=O); ^1^HNMR (400 MHz, DMSO-d_6_) *δ* (ppm): 3.74 (s, 3H, OCH_3_), 3.80 (s, 6H, 2OCH_3_), 6.51 (t, 1H, Ar-H,), 6.74 (d, *J* = 8 Hz, 2H, Ar-H), 7.23 (m, 1H, Ar-H,), 7.62–7.68 (m, 2H, Ar-H,), 7.76 (s, 1H, C-pyridine), 8.32 (d, *J* = 8 Hz, 4H, Ar-H), 11.24 (brs, 1H, NH, D_2_O exchangeable); ^13^CNMR (DMSO-d_6_) *δ* (ppm): 56.6, 60.6, 106.3, 106.7, 109.0, 115.0, 119.6, 126.9, 129.3, 129.6, 129.9, 130.4, 131.5, 133.9, 134.2, 135.6, 137.9, 150.5, 152.5, 153.4, 157.8, 159.1, 161.4, 170.6; MS (*m/z*): 542 (M + 2), 540 (M^+^); Anal. Calc. for: (C_29_H_21_ClN_4_O_5_): C, 64.39; H, 3.91; N, 10.36%; Found: C, 64.44; H, 3.95; N, 10.41%.

##### 4.1.3.4. 2-(p-Tolyl)-4–(3,4,5-trimethoxyphenyl)-5H-pyrido[2',3':4,5] pyrimido[2,1-b]quinazoline-5,7(12H)-dione (8d)

Yield (57%); m.p. >300 °C. IR (KBr) (cm^−1^): 3421 (NH), 1697(C=O); ^1^HNMR (400 MHz, DMSO-d_6_) *δ* (ppm): 2.39 (s, 3H, CH_3_), 3.74 (s, 3H, OCH_3_), 3.80 (s, 6H, 2OCH_3_), 6.75 (s, 2H, Ar-H), 7.21–6.76 (m, 2H, Ar-H,), 7.35 (d, *J* = 8 Hz, 2H, Ar-H), 7.46 (s, 1H, C6-pyridine), 7.57 (m, 2H, Ar-H), 7.99 (d, *J* = 8 Hz, 1H, Ar-H), 8.14 (d, *J* = 8 Hz, 1H, Ar-H), 11.65 (brs, 1H, NH, D_2_O exchangeable); ^13 ^C NMR (DMSO-d_6_) *δ* (ppm): 21.6, 56.2, 60.2, 106.0, 106.3, 106.7, 118.0, 127.9, 128.6, 129.3, 129.3, 129.6, 129.9, 134.2, 134.6, 136.5, 137.9, 140.9, 144.2, 150.1, 152.2, 153.4, 154.1, 159.1, 161.4, 169.4; MS (*m/z*): 520; Anal. Calc. for: (C_30_H_24_N_4_O_5_); C, 69.22; H, 4.65; N, 10.76%; Found: C, 69.25; H, 4.71; N, 10.82%.

#### 4.1.4. General procedure for synthesis of 2-Hydrazinyl -5,7-diarylpyrido[2,3-d]pyrimidin-4(3H)-one (9a–e)

A mixture of 2-thioxopyrido[2,3-d]pyrimidine derivatives **7a–e** (0.004 mol) and hydrazine hydrate (99%, 3 ml, 0.006 mol,) was heated under reflux in absolute ethanol (20 ml) for 10–15 h. After cooling, the precipitate was filtered and washed with hot ethanol to give compounds **9a–e**. All spectral data of hydrazino derivatives **9b–e** was reported in our previous work^73^. Herein we described our newly synthesised hydrazine precursor **9a.**

##### 4.1.4.1. 7-(4-Chlorophenyl)-5–(2,4-dichlorophenyl)-2-hydrazineylpyrido[2,3-d]pyrimidin-4(3H)-one 9a

Yield (45%); m.p. 265–267 °C. IR (KBr) (cm^−1^): 3398, 3367 (NH) and (NH_2_), 1685 (C=O); ^1^H NMR (400 MHz, DMSO-d_6_) *δ* (ppm): 7.59 (d, *J* = 8 Hz, 2H, Ar-H), 7.61–7.62 (m, 3H, Ar-H), 8.09 (s, 1H, H6-pyridine), 8.23 (brs, 2H, NH_2_, D_2_O exchangeable), 8.47 (d, *J* = 8 Hz, 2H, Ar-H), 9.18 (brs, 1H, NH, D_2_O exchangeable), 12.83, (brs, 1H, NH, D_2_O exchangeable); ^13 ^C NMR (DMSO-d_6_) *δ* (ppm): 106.5, 112.8, 120.3, 124.7, 129.0, 129.2, 130.1, 131.6, 132.9, 136.14, 136.19, 144.7, 147.8, 148.6, 157.9, 160.1, 174.6; MS (*m/z*): 438 (M + 6), 436 (M + 4), 434 (M + 2), 432 (M^+^). Anal. Calc. for: (C_19_H_12_Cl_3_N_5_O): C, 52.74; H, 2.80; N, 16.19%; Found: C, 52.78; H, 2.86; N, 16.25.

#### 4.1.5. General procedure for synthesis of 6.8-diaryl-pyrido[2,3-d][1, 2, 4]triazolo[4,3-a]pyrimidine-3,5-dione (10a–d)

A mixture of 2-hydrazinylpyrido[2,3-d]pyrimidine 9 b–e (1 mmol) and ethyl chloroformate (0.22 g, 2 mmol) in dry pyridine (10 ml) was heated under reflux for 9 h^60^. The reaction mixture was cooled and the obtained solid was filtered, washed with ethanol, dried, and crystallised from DMF: EtOH (1:2).

##### 4.1.5.1. 8-(4-Chlorophenyl)-6-(p-tolyl)-1,2-dihydropyrido[2,3-d][1, 2, 4]triazolo[4,3-a]pyrimidine-3,5-dione (10a)

Yield (80%); m.p. 347–349 °C. IR (KBr) (cm^−1^): 3383 (NH), 1685 (C=O), 1647 (C=O); ^1^HNMR (400 MHz, DMSO-d_6_) *δ* (ppm): 2.37 (s, 3H, CH_3_), 7.24–7.26 (m, 3H, Ar-H), 7.37 (d, *J* = 8 Hz, 1H, Ar-H), 7.48–7.62 (m, 4H, 2Ar-H + 2NH, D_2_O exchangeable), 7.92 (s, 1H, C-6pyridine,), 8.48 (d, *J* = 8 Hz, 1H, Ar-H), 8.67 (d, *J* = 8 Hz, 1H, Ar-H); ^13 ^C NMR (DMSO-d_6_) *δ* (ppm): 21.2, 108.6, 121.5, 128.1, 128.8, 129.4, 129.8, 134.5, 134.8, 135.7, 137.8, 145.7, 147.4, 148.0, 155.7, 159.4, 169.9; MS (*m/z*): 405 (M + 2), 403 (M^+^); Anal. Calc. for: (C_21_H_14_ClN_5_O_2_): C, 62.46; H, 3.49; N, 17.34%; Found: C, 62.54; H, 3.55; N, 17.40%.

##### 4.1.5.2. 6-(4-Methoxyphenyl)-8-(p-tolyl)-1,2-dihydropyrido[2,3-d][1, 2, 4]triazolo[4,3-a]pyrimidine-3,5-dione (10b)

Yield (54%); m.p. 366–368 °C. IR(KBr) (cm^−1^): 3398 (NH), 3375 (NH), 1697 (C=O), 1654 (C=O); ^1^HNMR (400 MHz, DMSO-d_6_) *δ* (ppm): 2.38 (s, 3H, CH_3_), 3.83 (s, 3H, OCH_3_), 6.96 (d, *J* = 8 Hz, 2H, Ar-H), 7.32–7.40 (m, 5H, Ar-H), 7.79 (s, 1H, C6-pyridine), 8.06 (brs, 2H, 2NH, D_2_O exchangeable), 8.57 (d, *J* = 8 Hz, 1H, Ar-H); MS (*m/z*): 399; Anal. Calc. for: (C_22_H_17_N_5_O_3_): C, 66.16; H, 4.29; N, 17.53%; Found: C; 66.24, H, 4.35; N, 17.60%.

##### 4.1.5.3. 8-(4-Chlorophenyl)-6–(3,4,5-trimethoxyphenyl)-1,2-dihydropyrido[2,3-d][1, 2, 4]triazolo[4,3-a]pyrimidine-3,5-dione (10c)

Yield (67%); m.p. 367–369 °C. IR(KBr) (cm^−1^): 3160 (2NH), 1759, 1697(2 C=O) ^1^HNMR (400 MHz, DMSO-d_6_) *δ* (ppm): 3.73 (s, 3H, OCH3), 3.78 (s, 6H, 2OCH_3_), 6.79 (s, 2H, Ar-H), 7.53–7.61 (m, 2H, Ar-H), 7.89 (s, 1H, C6-pyridine), 8.22–8.77 (m, 2H, Ar-H), 9.46 (s, 2H, 2NH, D_2_O exchangeable); ^13^C NMR (DMSO-d_6_) *δ* (ppm); 56.1, 59.7, 105.7, 106.8, 116.4, 117.5, 119.1, 128.0, 129.6, 137.7, 140.5, 148.8, 149.4, 152.3, 155.8, 158.4, 161.9, 169.6; MS (*m/z*): 481 (M + 2), 479 (M^+^). Anal. Calc. for: (C_23_H_18_ClN_5_O_5_): C, 57.57; H, 3.78; N, 14.59%; Found: C; 57.64, H, 3.85; N, 14.64%.

##### 4.1.5.4. 8-(p-Tolyl)-6–(3,4,5-trimethoxyphenyl)-1,2-dihydropyrido[2,3-d][1, 2, 4]triazolo [4,3-a]pyrimidine-3,5-dione (10d)

Yield (54%); m.p. 391–393 °C. IR (KBr) (cm^−1^): 3494 (NH), 3383 (NH), 1685 (C=O), 1660 (C=O); ^1^HNMR (400 MHz, DMSO-d_6_) *δ* (ppm): 2.37 (s, 3H, CH_3_), 3.73 (s, 6H, 2 OCH_3_), 6.73 (s, 2H, Ar-H), 7.34–7.45 (m, 2H, Ar-H), 7.95 (s, 1H, C6-pyridine), 8.07–8.11 (m, 2H, Ar-H), 9.26 (s, 1H, NH, D_2_O exchangeable), 11.07 (s, 1H, NH, D_2_O exchangeable); MS (*m/z*): 459. Anal. Calc. for: (C_24_H_21_N_5_O_5_): C, 62.74; H, 4.61; N, 15.24%; Found: C; 62.81, H, 4.67; N, 15.31%.

#### 4.1.6. General procedure for 3-amino-6,8-diaryl-2,3-dihydropyrido[2,3-d][1, 2, 4]triazolo[4,3-a]pyrimidin-5(1H)-one 11(a–e)

A mixture of 2-hydrazinylpyrido[2,3-d]pyrimidines **9a–e** (0.002 mol) and ammonium thiocyanate (2.38 g, 0.3 mol) in glacial acetic acid (15 ml) was heated under reflux for 10 h. The reaction mixture was cooled, poured onto iced water and the precipitate was filtered, dried and washed with hot ethanol^60^.

##### 4.1.6.1. 3-Amino-8–(4-chlorophenyl)-6–(2,4-dichlorophenyl)-10,10a-dihydropyrido [2,3-d][1, 2, 4]triazolo[4,3-a]pyrimidin-5(1H)-one (11a)

Yield (33%); m.p. 381–383 °C. IR (KBr) (cm^−1^): 3421, 3356 (NH, NH_2_), 1681 (C=O); ^1^HNMR (400 MHz, DMSO-d_6_) *δ* (ppm): 7.09 (brs, 1H, NH, D_2_O exchangeable), 7.33 (brs, 2H, NH_2_, D_2_O exchangeable), 7.52–7.63 (m, 3H, Ar-H), 7.70 (d, *J* = 8 Hz, 1H, Ar-H), 7.85 (s, 1H, C6-pyridine), 8.04 (s, 1H, Ar-H), 8.28 (d, *J* = 12 Hz, 2H, Ar-H); ^13^C NMR (DMSO-d_6_) *δ* (ppm): 119.5, 127.4, 128.4, 129.1, 129.8, 131.0, 132.1, 132.4, 134.4, 134.7, 136.4, 136.7, 138.0, 141.1, 147.6, 148.5, 154.5, 167.2; MS (*m/z*): 463 (M + 6), 461 (M + 4), 459 (M + 2), 457 (M^+^). Anal. Calc. for: (C_20_H_11_Cl_3_N_6_O): C, 52.48; H, 2.42; N, 18.36%; Found: C, 52.55; H, 2.47; N, 18.42%.

##### 4.1.6.2. 3-Amino-8–(4-chlorophenyl)-6-(p-tolyl)-2,3-dihydropyrido[2,3-d][1, 2, 4] triazolo[4,3-a]pyrimidin-5(1H)-one (11b)

Yield (33%); m.p. 383–385 °C. IR (KBr) (cm^−1^): 3422, 3394 (NH, NH_2_), 1697 (C=O); ^1^HNMR (400 MHz, DMSO-d_6_) *δ* (ppm): 2.34 (s, 3H, CH_3_), 7.16–7.22 (m, 2H, Ar-H), 7.33 (d, *J* = 8 Hz, 2H, Ar-H), 7.53–7.62 (d, *J* = 8 Hz, 2H), 7.88 (s, 1H, C-6pyridine), 8.28 (d, *J* = 8 Hz, 2H), 11.66 (brs, 1H, NH, D_2_O exchangeable), 11.65, (brs, 2H, NH_2_, D_2_O exchangeable); ^13^C NMR (DMSO-d_6_) *δ* (ppm): 20.6, 106.5, 109.3, 119.7, 122.7, 127.1, 128.3, 129.4, 134.7, 135.5, 137.4, 145.8, 147.3, 150.2, 153.7, 155.6, 167.4; MS (*m/z*): 404 (M + 2), 402 (M^+^); Anal. Calc. for: (C_21_H_15_ClN_6_O); C, 62.61; H, 3.75; N, 20.86%; Found: C, 62.66; H, 3.84; N, 20.91%.

##### 4.1.6.3. 3-Amino-6–(4-methoxyphenyl)-8-(p-tolyl)-2,3-dihydropyrido[2,3-d][1, 2, 4] triazolo[4,3-a]pyrimidin-5(1H)-one (11c)

Yield (47%); m.p. 381–383 °C. IR (KBr) (cm^−1^): 3425, 4332 (NH, NH_2_), 1697 (C=O);^1^H NMR (400 MHz, DMSO-d_6_) *δ* (ppm): 2.35 (s, 3H, CH_3_), 3.81 (s, 3H, OCH_3_), 6.91–6.96 (m, 2H, Ar-H), 7.31–7.40 (m, 4H, Ar-H), 7.81 (s, 1H, C6-pyridine), 8.14 (d, *J* = 8 Hz, 2H, Ar-H), 11.11 (brs, 2H, NH_2_, D_2_O exchangeable), 11.57 (brs, 1H, NH, D_2_O exchangeable); ^13^C NMR (DMSO-d_6_) *δ* (ppm): 21.2, 54.7, 112.8, 127.7, 129.4, 130.1, 133.4, 139.7, 140.7, 145.4, 145.7, 149.6, 150.0, 153.3, 153.6, 159.3, 162.6, 167.9; MS (*m/z*): 398. Anal. Calc. for: (C_22_H_18_N_6_O_2_): C, 66.32; H, 4.55; N, 21.09%; Found: C; 66.36, H, 4.60; N, 21.14%.

##### 4.1.6.4. 3-Amino-8–(4-chlorophenyl)-6–(3,4,5-trimethoxyphenyl) -2,3dihydropyrido[2,3-d][1, 2, 4]triazolo[4,3-a] pyrimidin-5(1H)-one(11d)

Yield (85%); m.p. 365–367 °C. IR (KBr) (cm^−1^): 3437, 3425 (NH, NH_2_), 1708 (C=O); ^1^H NMR (400 MHz, DMSO-d_6_) *δ* (ppm): 3.71 (s, 3H, OCH_3_), 3.78 (s, 6H, 2OCH_3_)_,_ 7.17 (s, 2H, Ar-H), 7.61–8 (m, 3H, Ar-H + C6-pyridine), 8.23–8.31 (m, 2H, Ar-H), 11.18 (brs, 2H, NH_2_, D_2_O exchangeable), 11.71 (brs, 1H, NH, D_2_O exchangeable); ^13^C NMR (DMSO-d_6_) *δ* (ppm): 56.0, 60.0, 106.4, 106.2, 128.8, 129.1, 129.4, 129.8, 134.1, 135.7, 140.7, 150.0, 151.7, 152.9, 153.6, 157.3, 160.9, 167.3; MS (*m/z*): 480 (M + 2), 478 (M^+^) Anal. Calc. for: (C_23_H_19_ClN_6_O_4_); C, 57.69; H, 4.00; N, 17.55%; Found: C, 57.74; H, 4.07; N, 17.59%.

##### 4.1.6.5. 3-Amino-8-(p-Tolyl)-6–(3,4,5-trimethoxyphenyl)-2,3-dihydropyrido[2,3-d][1, 2, 4]triazolo[4,3-a]pyrimidin-5(1H)-one (11e)

Yield (85%); m.p. 358–360 °C. IR (KBr) (cm^−1^): 3433, 3367 (NH, NH_2_), 1701 (C=O) ^1^H NMR (400 MHz, DMSO-d_6_) *δ* (ppm): 2.37 (s, 3H, CH_3_), 3.77 (s, 3H, OCH_3_), 3.78 (s, 6H, 2OCH_3_), 6.73–6.78 (m, 2H, Ar-H), 7.32–7.39 (m, 2H, Ar-H), 7.92 (s, 1H, C6-pyridine,), 8.19 (d, *J* = 8 Hz, 2H, Ar-H), 11.15 (brs, 2H, NH_2_, D_2_O exchangeable), 11.61 (s, 1H, NH, D_2_O exchangeable); ^13^C NMR (DMSO-d_6_) *δ* (ppm): 20.5, 55.7, 59.7, 106.5, 107.9, 114.8, 117.5, 127.5, 129.4, 134.1, 137.1, 140.1, 150.0, 152.0, 153.4, 153.7, 158.6, 161.3, 167.7; MS (*m/z*): 458; Anal. Calc. for: (C_24_H_22_N_6_O_4_); C, 62.87; H, 4.84; N, 18.33%; Found: C, 62.94; H, 4.91; N, 18.39%.

##### 4.1.6.6. 3-Phenyl-6,8-disubistitutedphenylpyrido[2,3-d][1, 2, 4]triazolo[4,3-a]pyrimidin-5(1H)-one (12a–e)

To a solution of hydrazine derivative 9b–e (0.001 mol) in dry pyridine (20 ml) benzoyl chloride was added (0.001 mol), and the resulting mixture was heated under reflux for 10–15 h. After cooling, the formed precipitate was filtered washed with hot ethanol to afford 12a–d, respectively.

##### 4.1.6.7. 8–(4-Chlorophenyl)-3-phenyl-6-(p-tolyl)pyrido[2,3-d][1, 2, 4]triazolo[4,3-a]pyrimidin-5(1H)-one (12a)

Yield (67%); m.p. 315–317 °C. IR(KBr) (cm^−1^): 3414(NH) 1750 (C=O); ^1^H NMR (400 MHz, DMSO-d_6_) *δ* (ppm): 2.40 (s, 3H, CH_3_), 7.27–8.30 (m, 14H, Ar-H), 11.21(brs, 1H, NH, D_2_O exchangeable); ^13^C NMR (DMSO-d_6_) *δ* (ppm): 21.3, 106.3, 118.0, 128.2, 128.2, 128.6, 129.3, 135.5, 136.6, 136.9, 138.5, 142.2, 150.8, 151.5, 153.8, 154.1, 155.2, 158.1, 162.8, 166,1, 188.4; MS *m/z* (%): 465(M + 2), 463 (M^+^); Anal. Calc. for: (C_27_H_18_ClN_5_O); C, 69.90; H, 3.91; N, 15.10%; Found: C, 69.97; H, 3.98; N, 15.14%.

##### 4.1.6.8. 6-(4-Methoxyphenyl)-3-phenyl-8-(p-tolyl)pyrido[2,3-d][1, 2, 4]triazolo[4,3-a]pyrimidin-5(1H)-one (12b)

Yield (33%); m.p. 325–327 °C. IR (KBr) (cm^−1^): 3414, NH, 1720 (C=O);^1^H NMR (400 MHz, DMSO-d_6_) *δ* (ppm); 2.39 (s, 3H, CH_3_), 3.85 (s, 3H, OCH_3_), 7.03–8.32 (m, 15H 14Ar-H + NH- D_2_O exchangeable); ^13^C NMR (DMSO-d_6_) *δ* (ppm): 21.6, 55.5, 113.2, 127.1, 127.6, 128.5, 129.0, 129.5, 129.8, 129.9, 130.3, 130.9, 131.5, 132.7, 135.4, 143.6, 145.2, 150.4, 154.6, 158.4, 160.3, 170.6; MS (*m/z*): 459; Anal. Calc. for: (C_28_H_21_N_5_O_2_); C, 73.19; H, 4.61; N, 15.24%; Found: C, 73.25; H, 4.66; N, 15.27%.

##### 4.1.6.9. 8-(4-Chlorophenyl)-3-phenyl-6–(3,4,5-trimethoxyphenyl)pyrido[2,3-d][1, 2, 4]triazolo[4,3-a]pyrimidin-5(1H)-one (12c)

Yield (33%); m.p. 313–315 °C. IR (KBr) (cm^−1^): 3417 (NH), 1693 (C=O); ^1^H NMR (400 MHz, DMSO-d_6_) *δ* (ppm): 3.7 (s, 3H, OCH_3_), 3.8 (s, 6H, 2OCH_3_), 7.47–59 (m, 3H, Ar-H), 7.59–7.63 (m, 4H, Ar-H), 7.93–8.06 (m, 3H, 2Ar-H + 1NH-D_2_O exchangeable), 8.55–8.59 (m, 1H, C6-pyridine), 8.91–8.92 (m, 2H, Ar-H); ^13^CNMR (DMSO-d_6_) *δ* (ppm): 55.6, 59.6, 113.3, 123.9, 127.2, 128.9, 129.3, 129.5, 129.7, 129.8, 129.9, 130.3, 130.9, 132.6, 143.3, 146.5, 152.7, 160.2, 169.2; MS (*m/z*): 541(M + 2), 539 (M^+^); Anal. Calc. for: (C_29_H_22_ClN_5_O_4_): C, 64.51; H, 4.11; N, 12.97%; Found: C, 64.57; H, 4.16; N, 13.03%.

##### 4.1.6.10. 3-Phenyl-8-(p-tolyl)-6–(3,4,5-trimethoxyphenyl)pyrido[2,3-d][1, 2, 4]triazolo [4,3-a]pyrimidin-5(1H)-one(12d)

Yield (33%); m.p. 322–324 °C. IR (KBr) (cm^−1^): 3464 (NH), 1701 (C=O); ^1^H NMR (400 MHz, DMSO-d_6_) *δ* (ppm): 2.37 (s, 3H, CH_3_), 3.75 (s, 3H, OCH_3_), 3.9 (s, 6H, 2OCH_3_), 6.76–7.07 (m, 2H, Ar-H), 7.09–7.87 (m, 8H, Ar-H), 8.10–8.22 (m, 2H, Ar-H), 11.16 (brs, 1H, NH, D_2_O exchangeable); ^13^C NMR (DMSO-d_6_) *δ* (ppm): 20.8, 56.1, 60.1, 105.5, 106.5, 107.9, 108.9, 117.5, 118.8, 120.4, 127.4, 128.4, 129.1, 130.5, 132.3, 133.8, 134.4, 137.1, 140.1, 150.0, 151.7, 153.3, 158.6,162.6; MS (*m/z*): 519; Anal. Calc. for: (C_30_H_25_N_5_O_4_); C, 69.35; H, 4.85; N, 13.48%; Found: C, 69.41; H, 4.91; N, 13.52%.

### 4.2. Biological evaluation

#### 4.2.1. In vitro cytotoxic activity

In vitro cytotoxicity was carried out using MTT assay protocol^63^ as described in Supplementary data.

#### 4.2.2. In vitro EGFR kinase assay

In vitro EGFR inhibitory activity was assessed using Homogeneous time-resolved fluorescence (HTRF) assay^64^ as described in Supplementary data.

#### 4.2.3. Cell cycle analysis

The effect of compound **8a** on cell cycle distribution was performed using propidium iodide (PI) staining technique as described in Supplementary data^65^^,^^74^^,^^75^.

#### 4.2.4. Apoptosis analysis

The effect of compound **8a** on cell apoptosis was investigated as described in Supplementary data^76–78^.

### 4.3. Docking studies

Molecular docking studies of the synthesised compounds were carried out against EGFR^WT^ (PDB ID: 4HJO, resolution 2.75 Å and EGFR^T790M^ (PDB ID: 3W2O, resolution 2.35 Å) as described in Supplementary data^22^.

## Supplementary Material

Supplemental Material
