# Supplementary material for: Design, synthesis, and anti-cancer evaluation of new pyrido[2,3-d]pyrimidin-4(3H)-one derivatives as potential EGFRWT and EGFRT790M inhibitors and apoptosis inducers
Source: J Enzyme Inhib Med Chem. 2022 Jul 12;37(1):1053–76. doi: 10.1080/14756366.2022.2062752 (PMC9291687; doi:10.1080/14756366.2022.2062752)

Design, synthesis, and anti-cancer evaluation of new pyrido[2,3-*d*]pyrimidin-4(3*H*)-one derivatives as potential EGFR^{WT} and EGFR^{T790M} inhibitors and apoptosis inducers

Heba S. A. Elzahabi[a]*, Eman S. Nossier[a], Rania A. Alasfoury[a], Sara M. Saad[b], Eslam B. Elkaeed[c], Ahmed M. Metwaly[d, e], Mohamed Hagras[f], Ibrahim H. Eissa [g]*

[a] Pharmaceutical Medicinal Chemistry & Drug Design Department, Faculty of Pharmacy (Girls), Al-Azhar University, Cairo, Egypt

[b] Biochemistry and Molecular Biology Department, Faculty of Pharmacy (Boys), Al-Azhar University, Cairo 11884, Egypt.

[c] Department of Pharmaceutical Sciences, College of Pharmacy, AlMaarefa University, Ad Diriyah 13713, Riyadh, Saudi Arabia

[d] Pharmacognosy and Medicinal Plants Department, Faculty of Pharmacy (Boys), Al-Azhar University, Cairo, Egypt

[e] Biopharmaceutical Products Research Department, Genetic Engineering and Biotechnology Research Institute, City of Scientific Research and Technological Applications (SRTA-City), Alexandria, Egypt

[f] Pharmaceutical Organic Chemistry, Faculty of Pharmacy (Boys), Al-Azhar University, Cairo, Egypt

[g] Pharmaceutical Medicinal Chemistry & Drug Design Department, Faculty of Pharmacy (Boys), Al-Azhar University, Cairo, Egypt

*** Corresponding authors:**

Ibrahim H. Eissa

Pharmaceutical Medicinal Chemistry & Drug Design Department, Faculty of Pharmacy (Boys), Al-Azhar University, Cairo, 11884, Egypt

Email: Ibrahimeissa@azhar.edu.eg

Heba S. A. Elzahabi

Pharmaceutical Medicinal Chemistry & Drug Design Department, Faculty of Pharmacy (Girls), Al-Azhar University, Cairo, Egypt

Email: hebaelzahabi@gmail.com

Content

**1**	Chemicals and apparatus
**2**	Biological tests
**3**	Docking procedures
**4**	Spectral data

1. Chemicals and apparatus

Melting points were measured in open capillary tubes using Electro thermal apparatus and are uncorrected. Elemental microanalyses were carried out at the regional center for Mycology and Biotechnology, AL-Azhar University. The infra-red (IR) spectra were recorded using potassium bromide disc technique on Schimadzu 435 IR Spectrophotometer at Micro analytical unit, Cairo University. ^{1}HNMR and ^{13}CNMR spectra were performed on Agilent Technologies 400 MHz NMR spectrophotometer at Mansoura University, faculty of pharmacy. DMSO-d_6 was used as a solvent, and the chemical shifts were measured in ppm, relative to TMS as an internal standard. For the proton magnetic resonance, D_2O was carried out for NH and OH exchangeable protons. Mass spectra were recorded on a DI-50 unit of Shimadzu GC/ MS-QP 2010 plus Spectrometer (Japan) or on single quadrpole mass Spectrometer ISQ LT (Thermo scientific) and carried out at the regional center for Mycology and Biotechnology, AL-Azhar University

All reactions were monitored by TLC using pre-coated Aluminum sheet silica gel Merck 6 F 254 and were visualized by UV lamp. Chemical naming and calculated microanalysis of new compounds were performed by ChemDraw Program 16.0 software [Chemical Structure Drawing Standard; Cambridge Soft Corporation, USA (2016).

2. <u>Biological testing</u>

2.1. In vitro antiproliferative activities

The *in vitro* antiproliferative activities of all the synthesized compounds against a panel of four human tumor cell lines namely; A-549, PC-3, HCT-116, and MCF-7 were evaluated

quantitatively as described in the literature, using MTT assay protocol [61-63, 85, 86]. two commercially available drugs (erlotinib) were used in this test as positive controls. The anti-proliferative activity was assessed quantitatively as follows;

Human cancer cell lines were dropped in 96-well plates at a density of 3-8 × 10^3 cells/well. Next, the wells were incubated for 12 h in a 5% CO_2 incubator at 37 °C. Then, for each well, the growth medium was exchanged with 0.1 ml of fresh medium containing graded concentrations of the test compounds to be or equal DMSO and incubated for two days. Then 10 µl MTT solution (0.5 mg/ml) was added to each well, and the cells were incubated for additional 4 h. The crystals of MTT-formazan were dissolved in 100 µl of DMSO; the absorbance of each well was measured at 490 nm using an automatic ELISA reader system (TECAN, CHE). The IC_{50} values were calculated using the nonlinear regression fitting models (Graph Pad, Prism Version 5). The data represented the mean of three independent experiments in triplicate and were expressed as means ± SD. The IC_{50} value was defined as the concentration at which 50% of the cells could survive.

The results were expressed as growth inhibitory concentration (IC_{50}) values and summarized in **Table 2**.

4.1.1. EGFR^{WT} and EGFR^{T790M} kinase inhibitory assay

The most active cytotoxic compounds **8a**, **8d** and **9a** that showed promising IC_{50} values against four cancer cell lines were further examined for their inhibitory activities against both EGFR^{WT} and EGFR^{T790M}. Homogeneous time resolved fluorescence (HTRF) assay was applied in this test. EGFR^{WT}, EGFR^{T790M} (Sigma). firstly, the EGFR^{WT} and/or EGFR^{T790M} and their substrates were incubated with the tested compounds in enzymatic buffer for 5 min. ATP (1.65 µM) was added into the reaction mixture to allow starting the enzymatic reaction. The assay was conducted for 30 min at room temperature. The reaction was stopped by addition of detection reagents which contain EDTA. The detection step continued for 1 h, and then the IC_{50} values were determined by GraphPad Prism 5.0. Three independent experiments were performed for each concentration.

4.1.2. *In-vitro* DNA-Flow cytometric (cell cycle) analysis.

PC-3 cells were exposed to the most active member **8a** at concentrations of 7.98 µM for 24 h. Then, the tested cells were collected by trypsinization and washed in PBS. Ice-cold absolute ethanol was used for fixation of the collected cells. The cells were stained with Cycle TESTTM

PLUS DNA Reagent Kit (BD Biosciences, San Jose, CA) according to the manufacturer's instructions. Cell-cycle distribution was evaluated using a flow cytometer.

4.1.3. Annexin V-FITC apoptosis assay.

To detect the apoptosis induced by compound **8a**, PC-3 cells were seeded and incubated overnight and then treated with compound **8a** at concentrations of 7.98 µM for 24 h. DMSO was chosen as the negative control. After that, the cells were collected and washed with PBS two successive times. The cells were exposed to centrifugation. Apoptosis detection kit (BD Biosciences, San Jose, CA) was used in this test. According to the manufacturer's protocol the cells were stained by Annexin V-FITC and propidium iodide (PI) in the binding buffer for 20min at room temperature in the dark. Using a flow cytometer, Annexin V-FITC and PI binding were analyzed. flowjo software was used to analyze the frequencies in all quadrants [88].

4.2.5. Caspase-3 determination

The percentage of caspase-3 activation was determined using the Caspase- Invitrogen Caspase-3 ELISA Kit (KHO1091) following the manufacturer's instructions.

2.2.4. Cell cycle analysis

2.2.4.1.Effect of compound 8a on cell cycle progression of PC-3.

**Sample**	Cell cycle distribution (%)			
	%G0-G1	%S	%G2-M	%Pre-G1
**8a/PC-3**	41.38	53.69	4.93	41.06
**Cont. (PC-3)**	49.22	41.03	9.75	1.78

2.2.4.2.Percentage of induced cell death by compound 8a on PC3 cells.

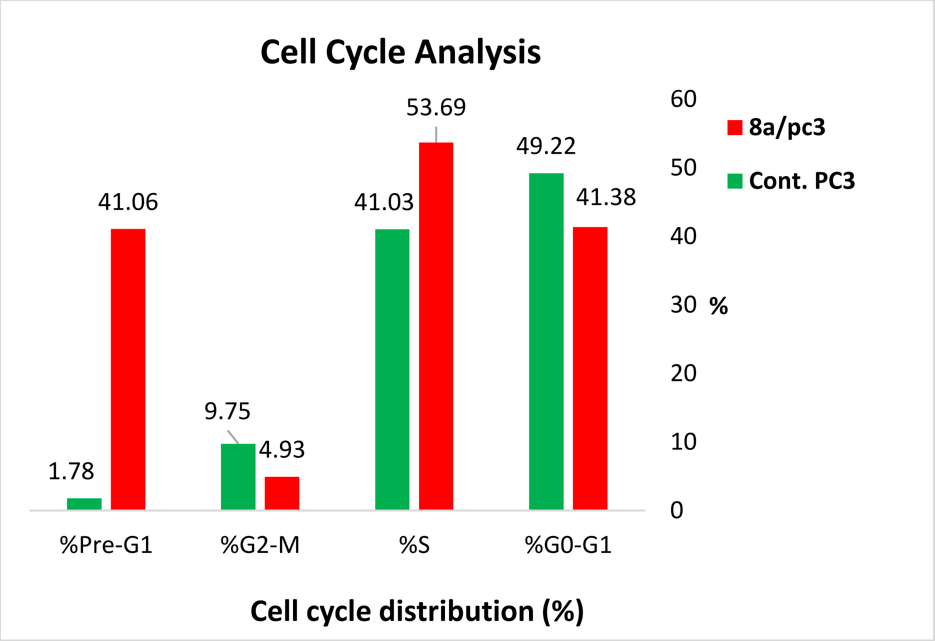

2.2.5. Annexin V-FITC apoptosis assay

2.2.5.1. Apoptosis and necrosis percent induced by compound 8a

Sample	Apoptosis			Necrosis
	Total	Early	Late	
**8a/PC3**	41.06	13.92	22.49	4.65
**Cont. PC3**	1.78	0.43	0.15	1.20

2.2.5.2.Apoptosis effect of compound 8a on PC3 cells.

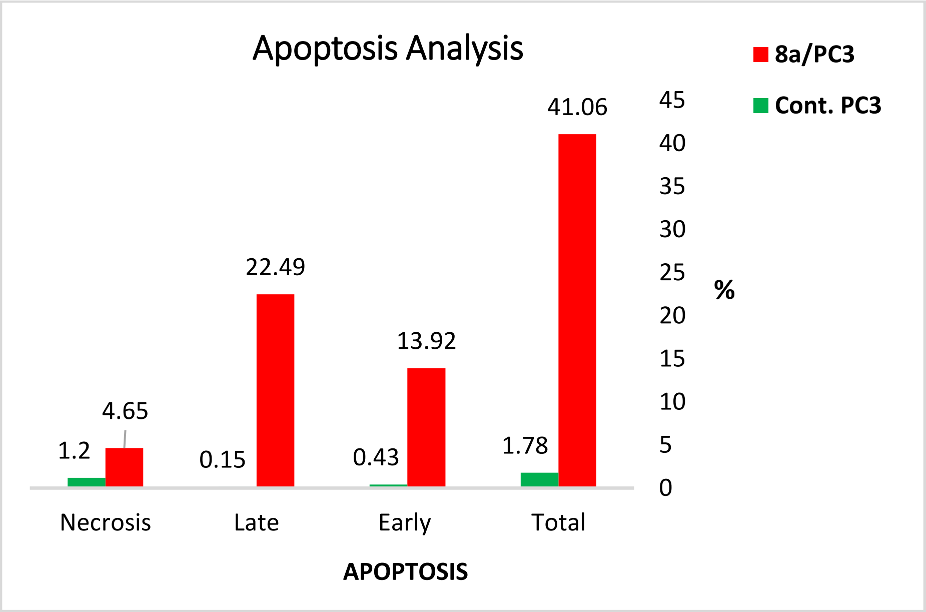

2.2.6. Caspase-3 determination

2.2.6.1.The effect of compound 8a on the level of caspase-3 comparing to negative and positive control

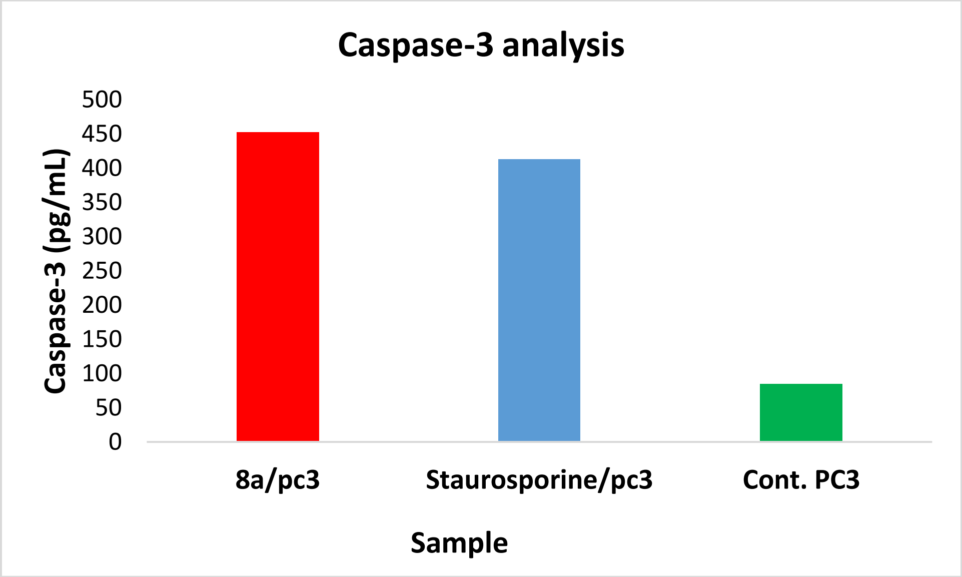

.

3. Docking studies

The crystal structures of the target enzymes EGFR^{WT} (PDB ID: 4HJO, resolution 2.75 Å and EGFR^{T790M} (PDB ID: 3W2O, resolution 2.35 Å) were downloaded from Protein Data Bank (http://www.pdb.org). Molecular Operating Environment (MOE) was used for the docking analysis [91]. In these studies, the free energies and binding modes of the designed molecules against EGFR^{WT} and EGFR^{T790M} were determined. At first, the water molecules were removed from the crystal structures of EGFR^{WT} and EGFR^{T790M}, retaining only one chain in each enzyme. Erlotinib and TAK-285 (The co-crystallized ligands) were utilized as references in the docking processes against both EGFR^{WT} and EGFR^{T790M}, respectively. After that, in order to prepare the target molecules for binding with the designed compounds, the target proteins were subjected to protonation step. Then, the hydrogen atoms were hidden to make the areas of interaction clearer. Next, the energy of all systems was minimized followed by identification the binding pockets of the target proteins.

The structures of the designed compounds and the co-crystallized ligands, erlotinib and TAK-285 were drawn using ChemBioDraw Ultra 14.0 and saved as SDF format. Then, the saved files were opened using MOE and 3D structures were protonated. Next, the energy of the molecules was minimized. Validation process was performed for each target by running the docking process for only the co-crystallized ligand. Low RMSD values between docked and crystal conformations indicate valid performance. The docking procedures were carried out utilizing a default protocol. In each case, 30 docked structures were generated using genetic algorithm searches. The output from of MOE was further analyzed and visualized using Discovery Studio 4.0 software.

Score of validation against wild EGFR

Database Viewer : $DESKTOP/.../wild/validation222222.mdb

File Edit Display Compute Window Help

	mol	rseq	mseq	S	rmsd
1	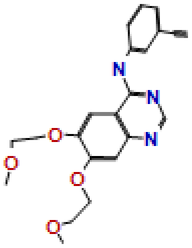	1	1	-8.1453	0.8835
2	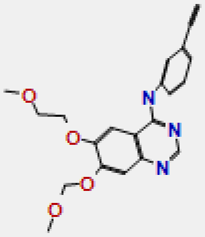	1	1	-7.8168	3.2808
3	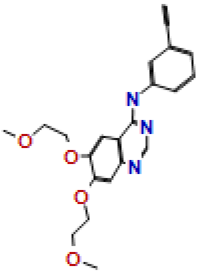	1	1	-7.7506	6.0401

Score of validation process against mutant EGFR

Database Viewer : $DESKTOP/.../mutant/validation 22222.mdb

File Edit Display Compute Window Help

	mol	rseq	mseq	S	rmsd
7	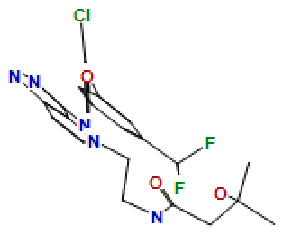	1	1	-7.2770	1.0580
8	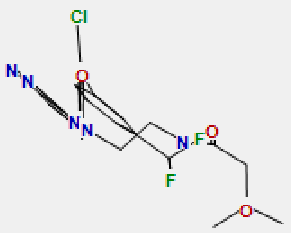	1	1	-7.1492	1.5465
9	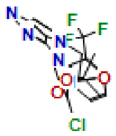	1	1	-7.1470	4.6845

1H NMR of compound 7a

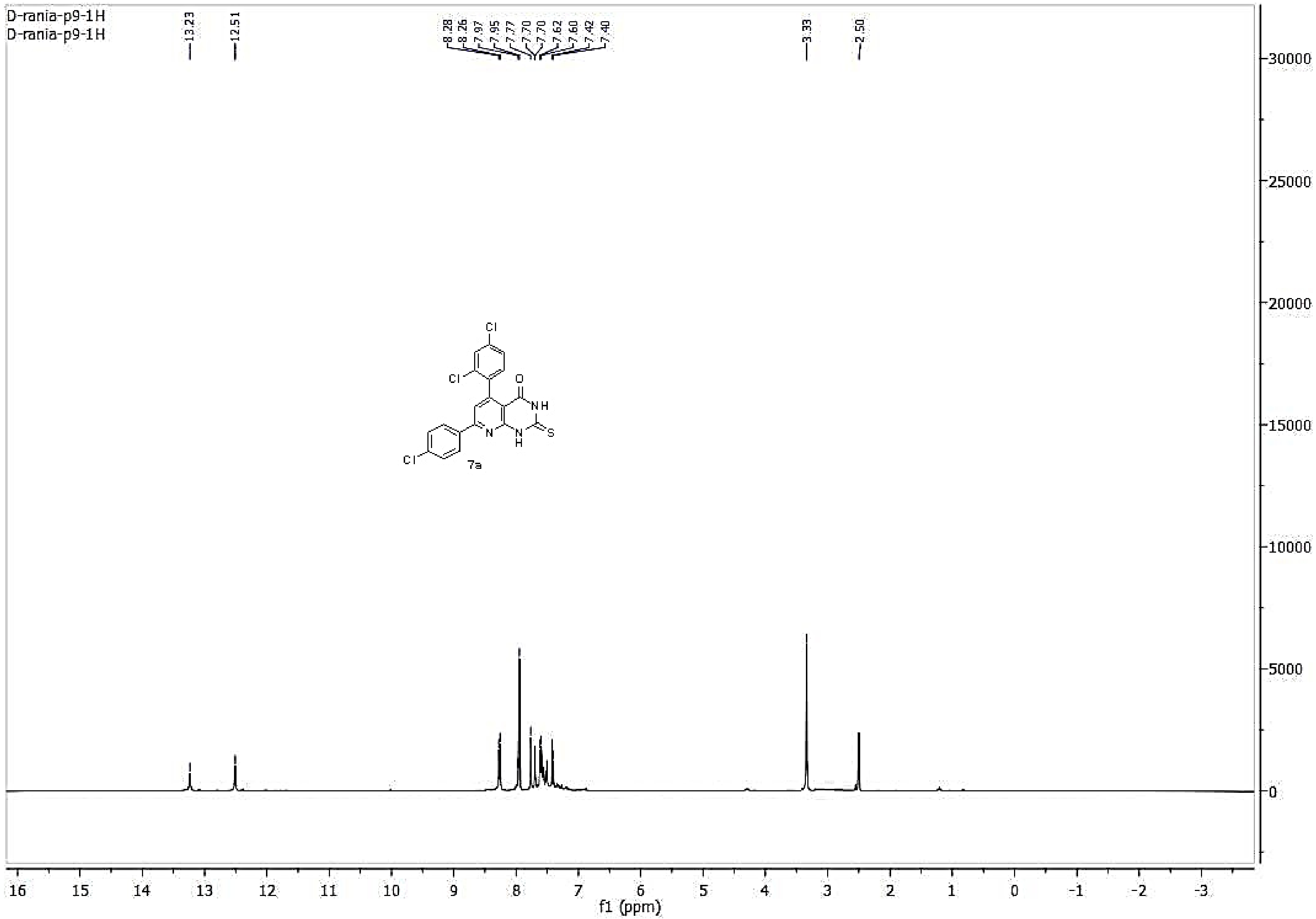

13C NMR of compound 7a

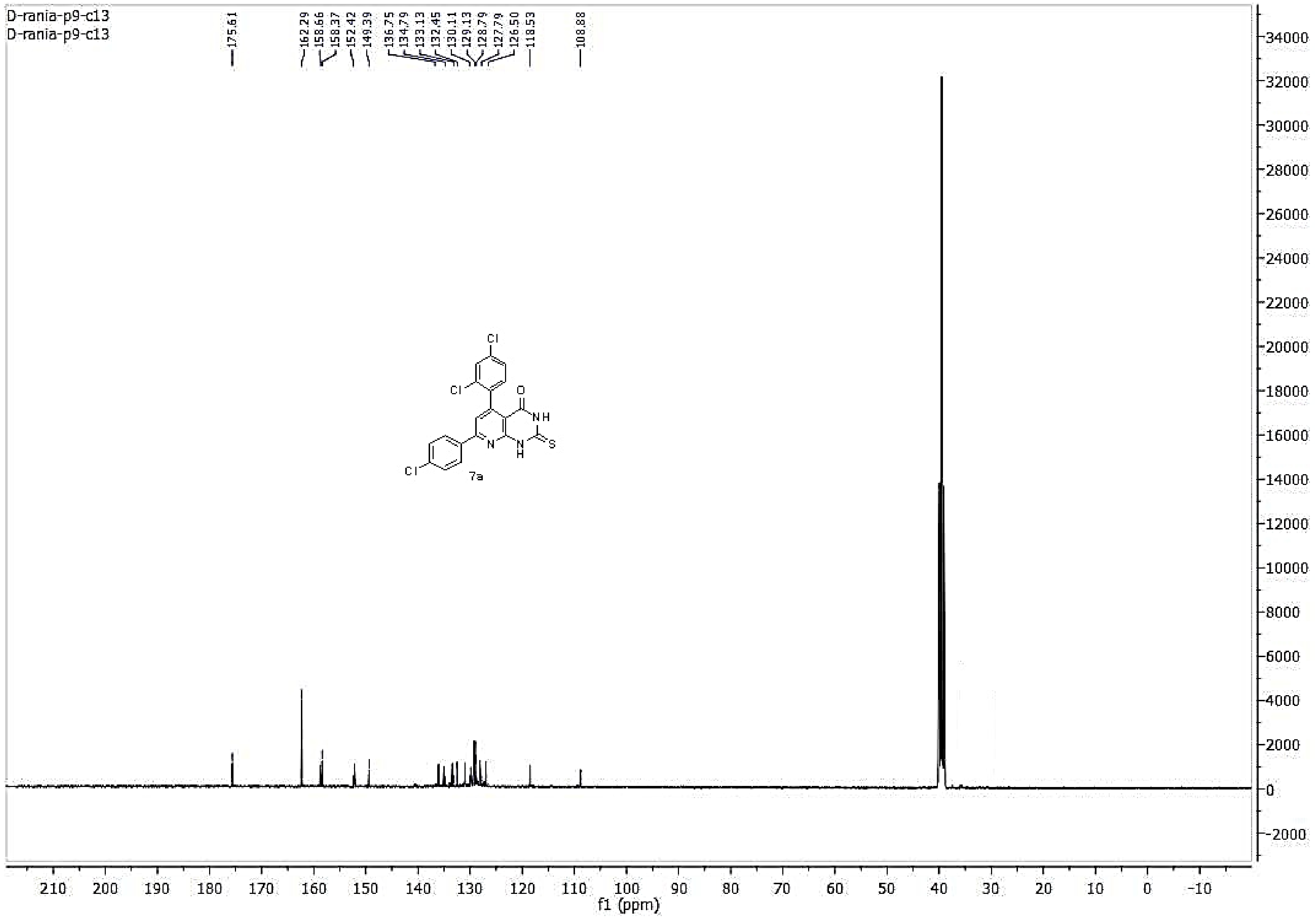

1H NMR of compound 8d

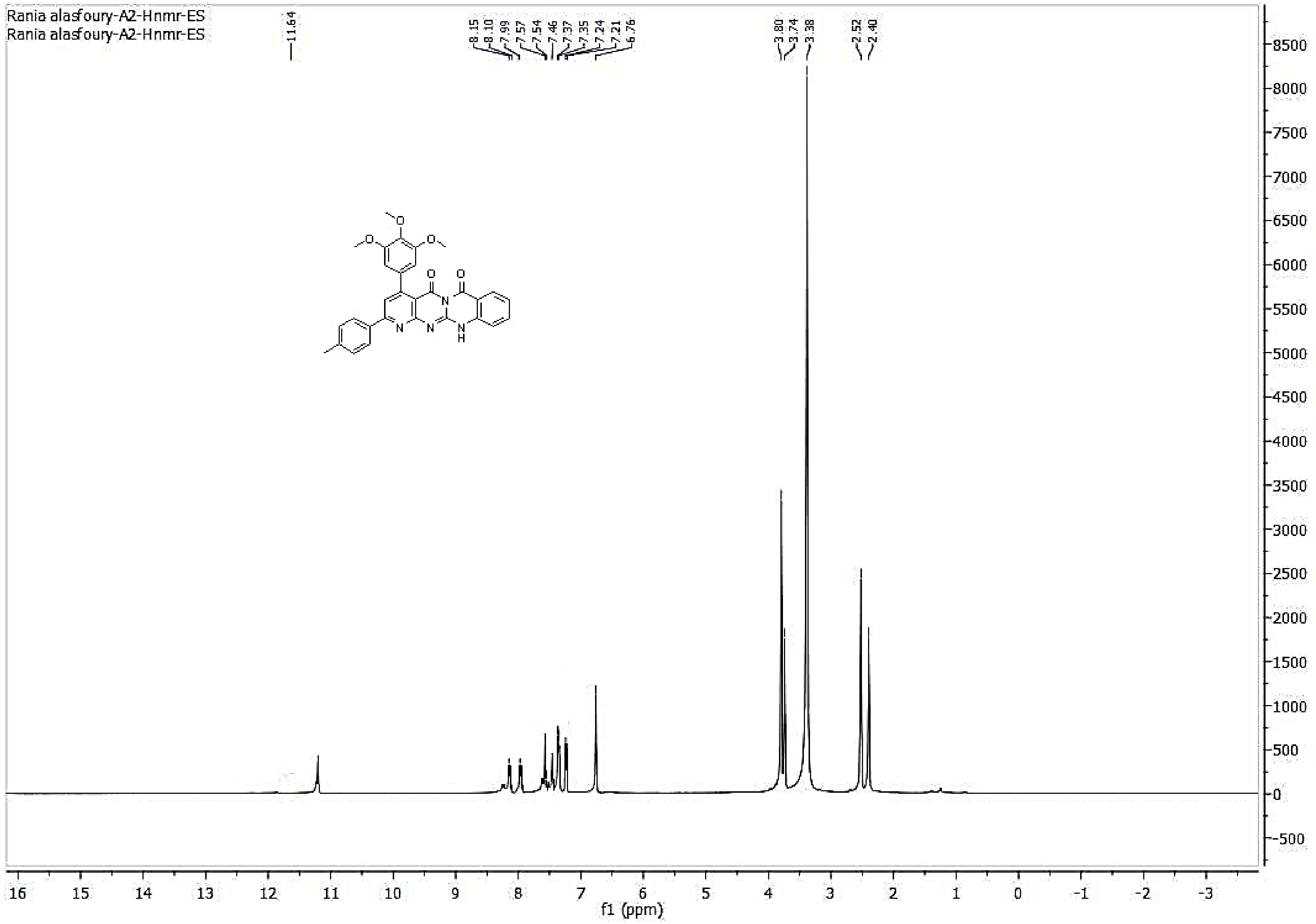

13C NMR of compound 8d

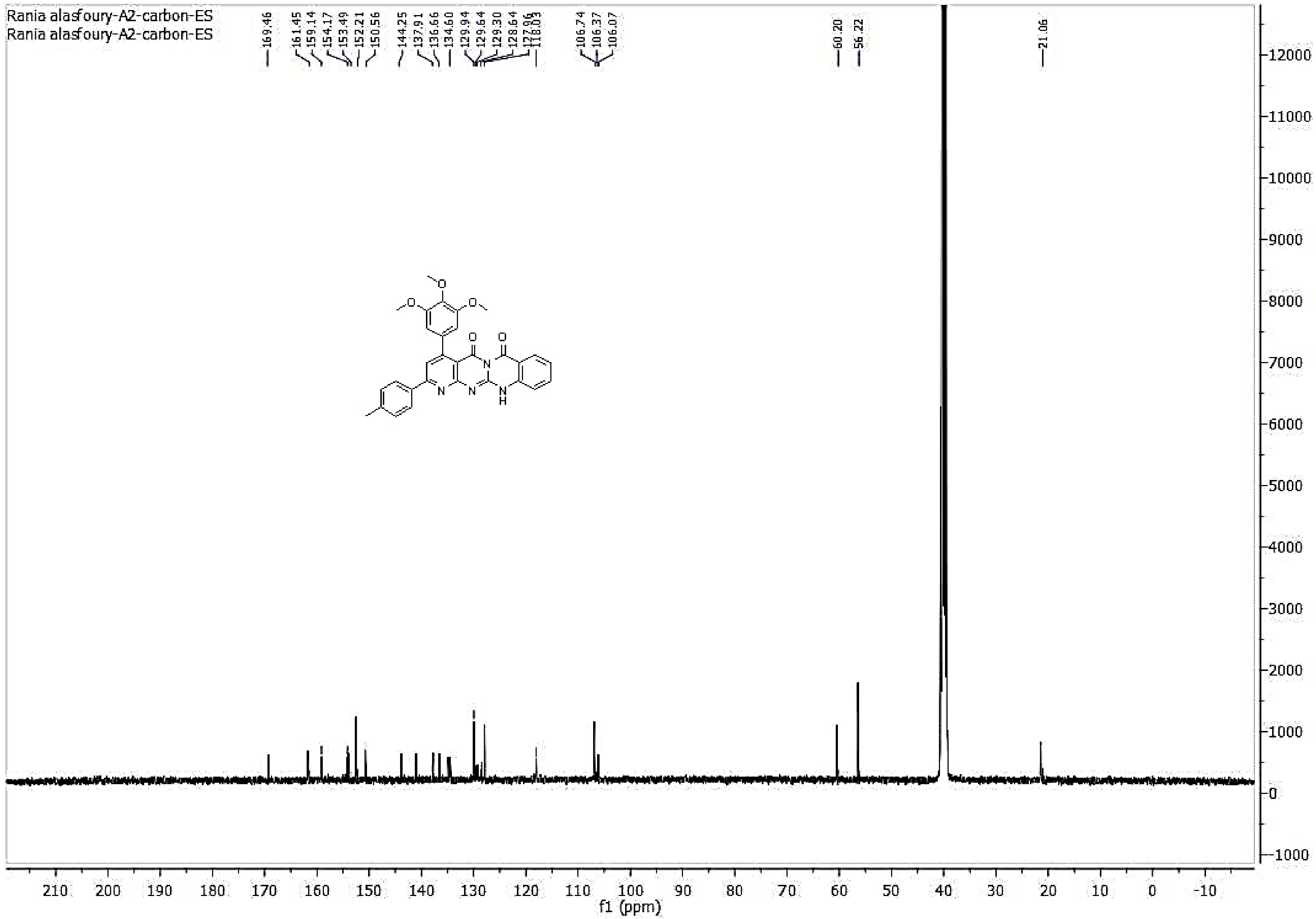

1H NMR of compound 8c

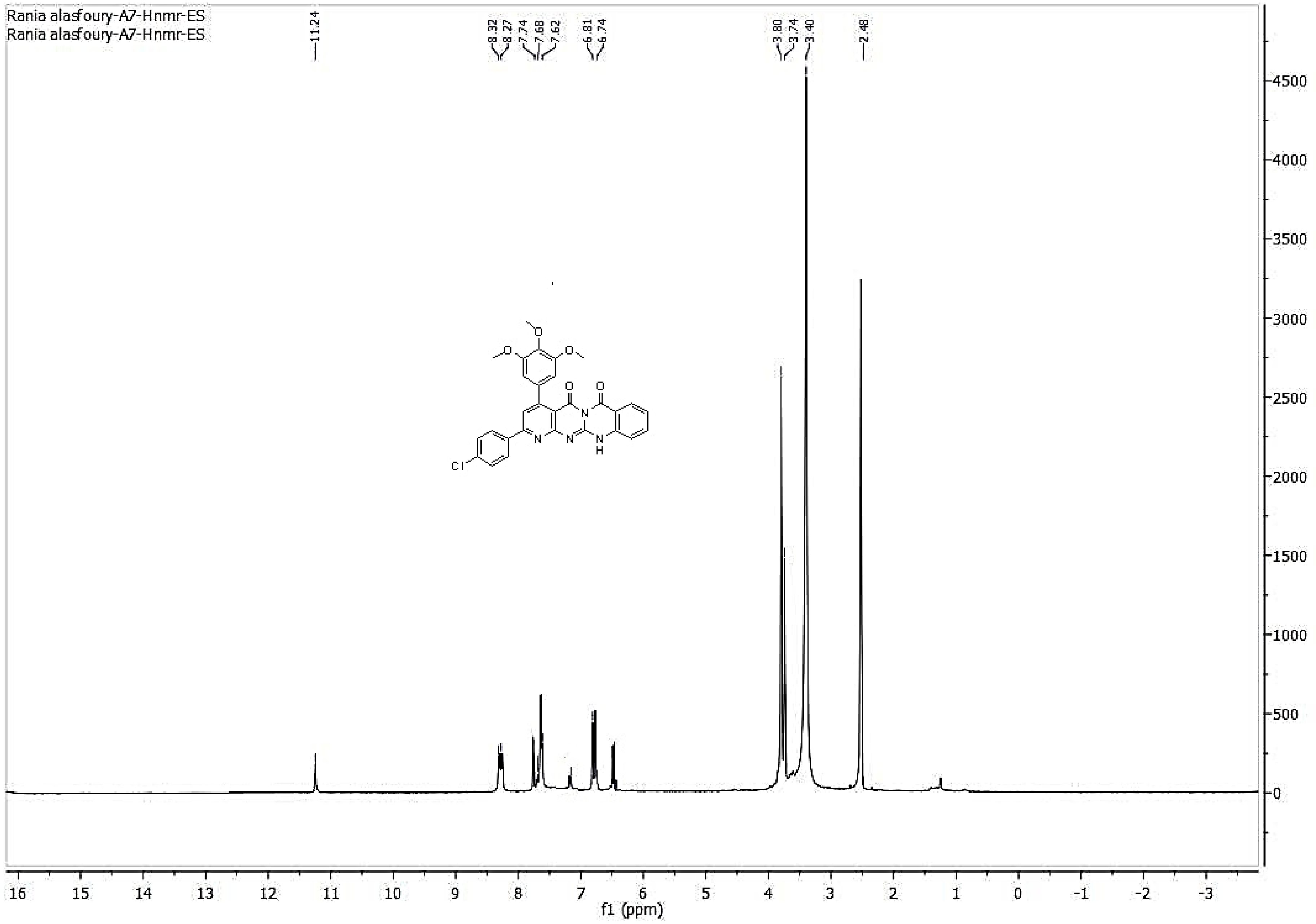

13C NMR of compound 8c

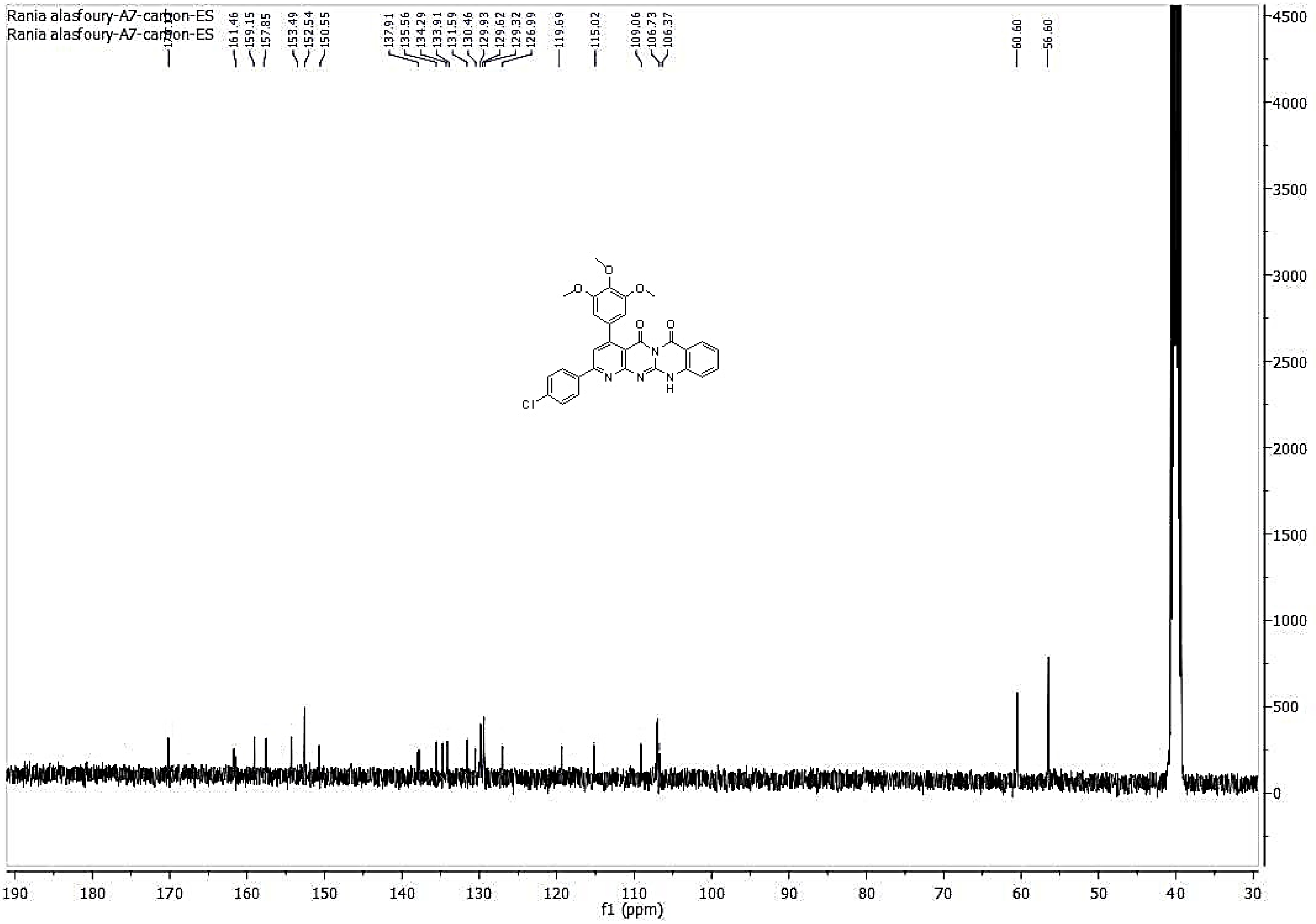

1H NMR of compound 8a

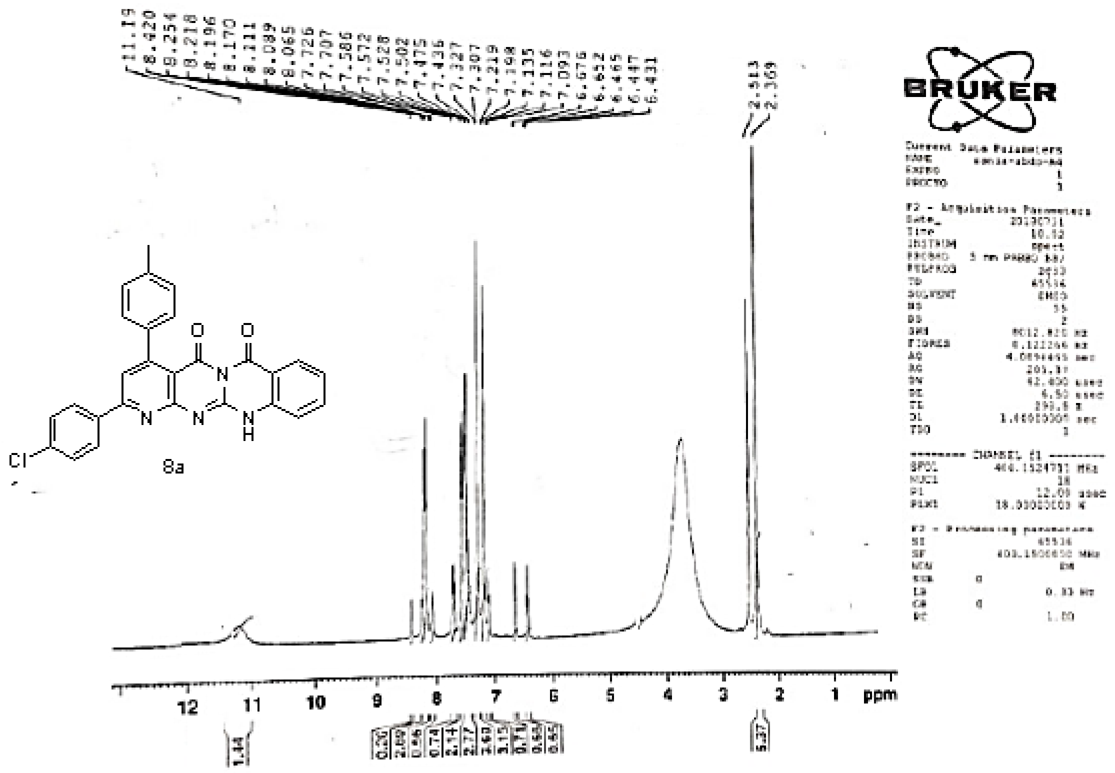

13C NMR of compound 8a

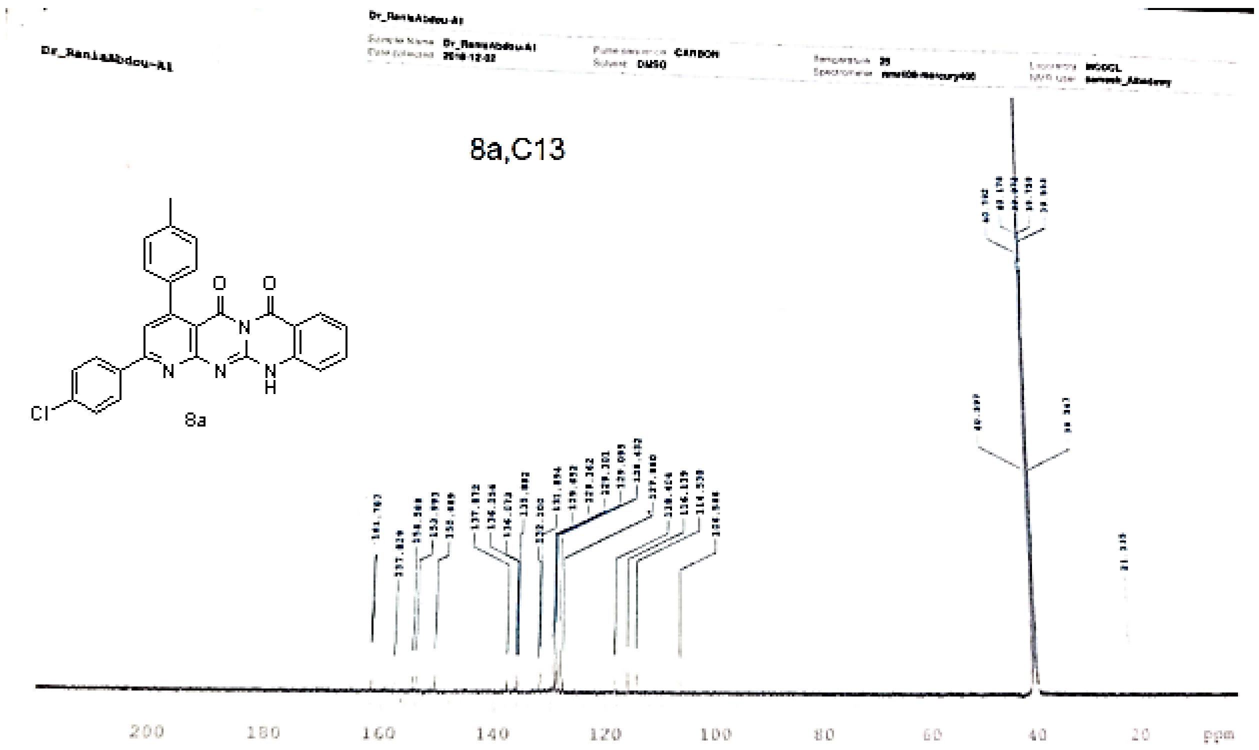

1H NMR of compound 8b

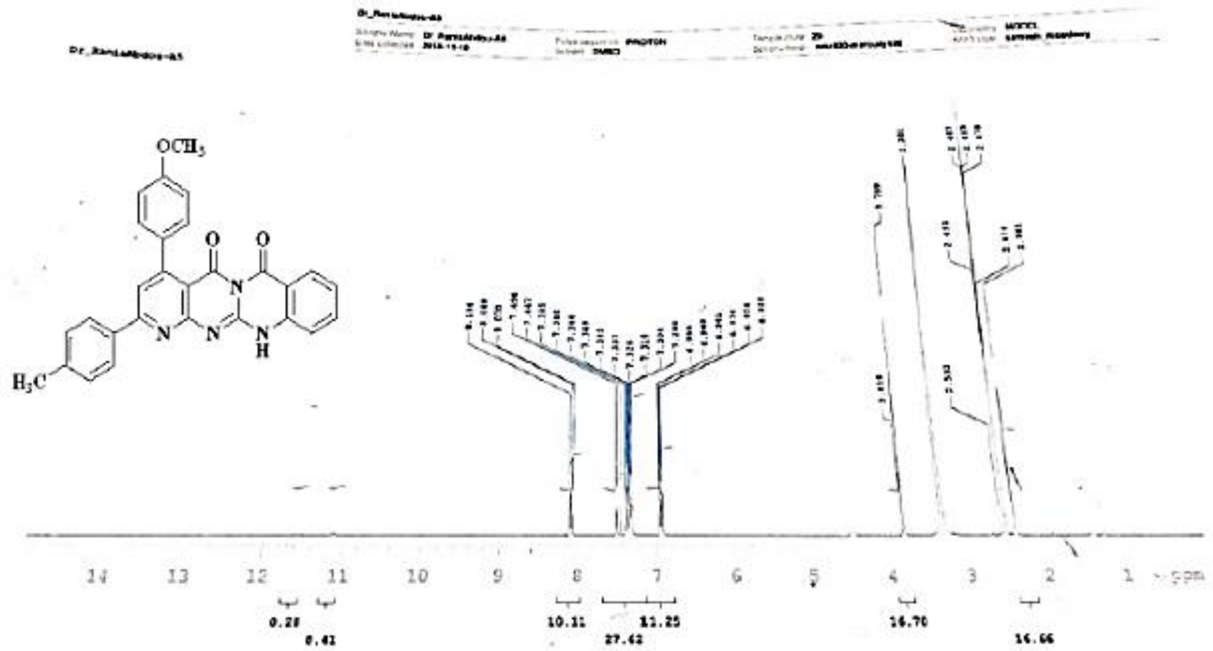

1H NMR of compound 9a

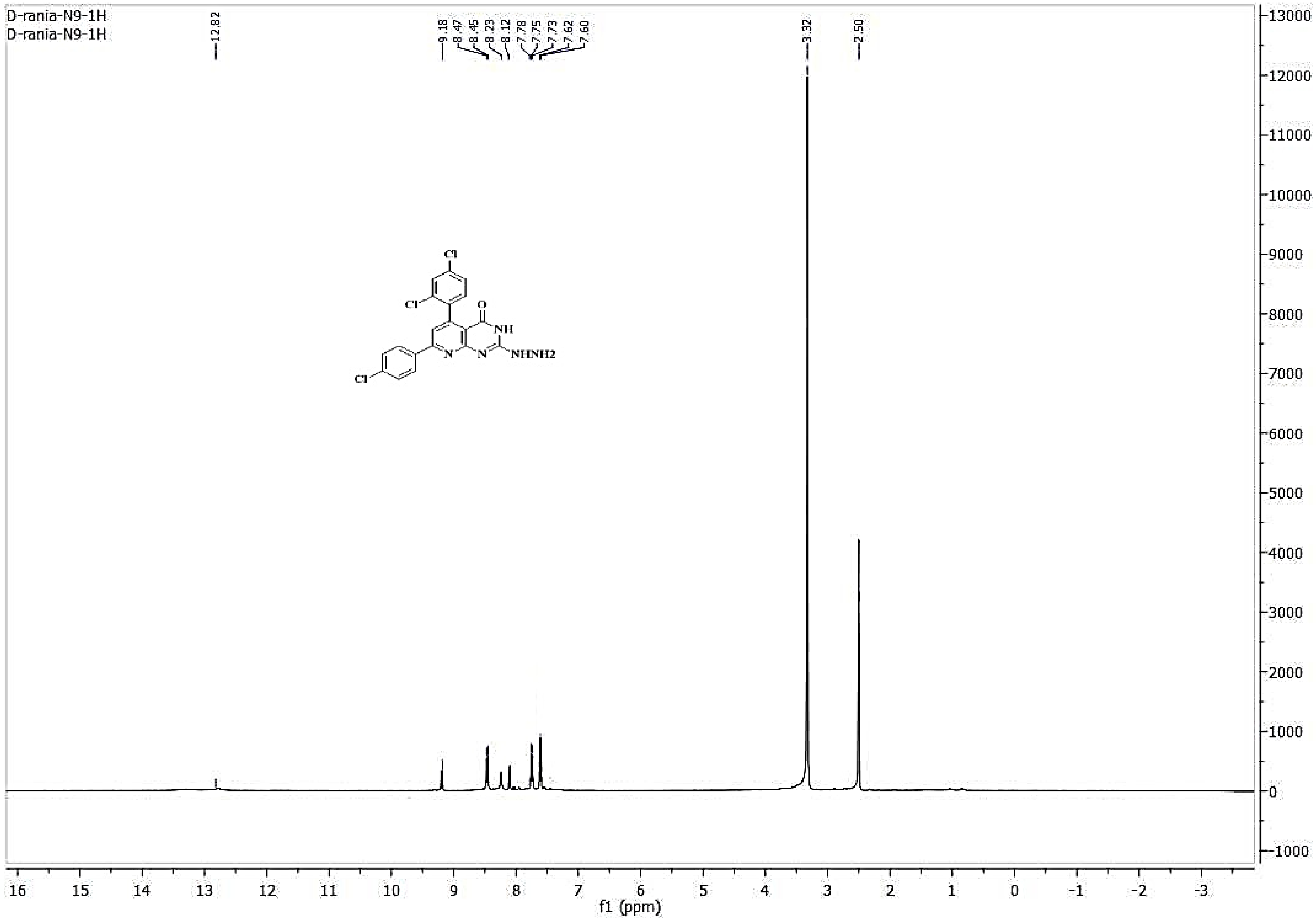

13C NMR of compound9a

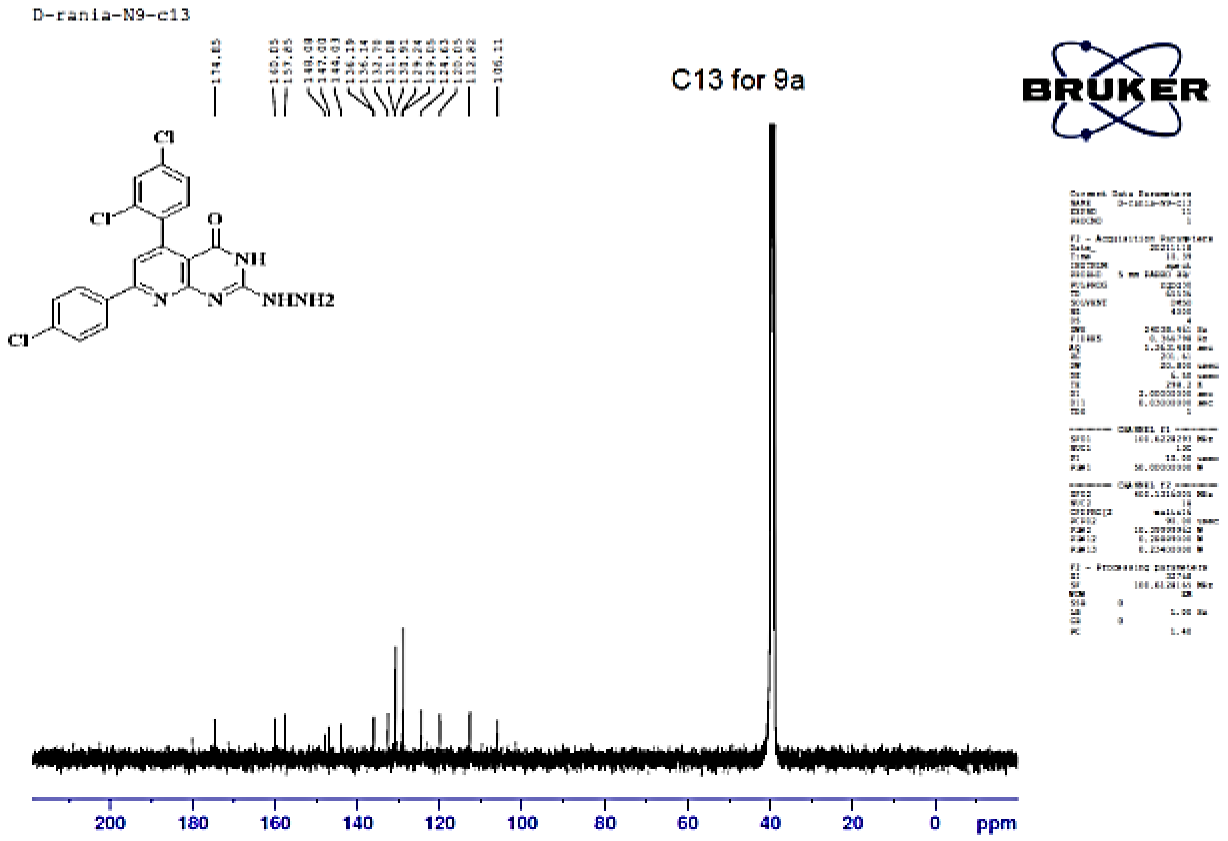

1H NMR of compound 10a

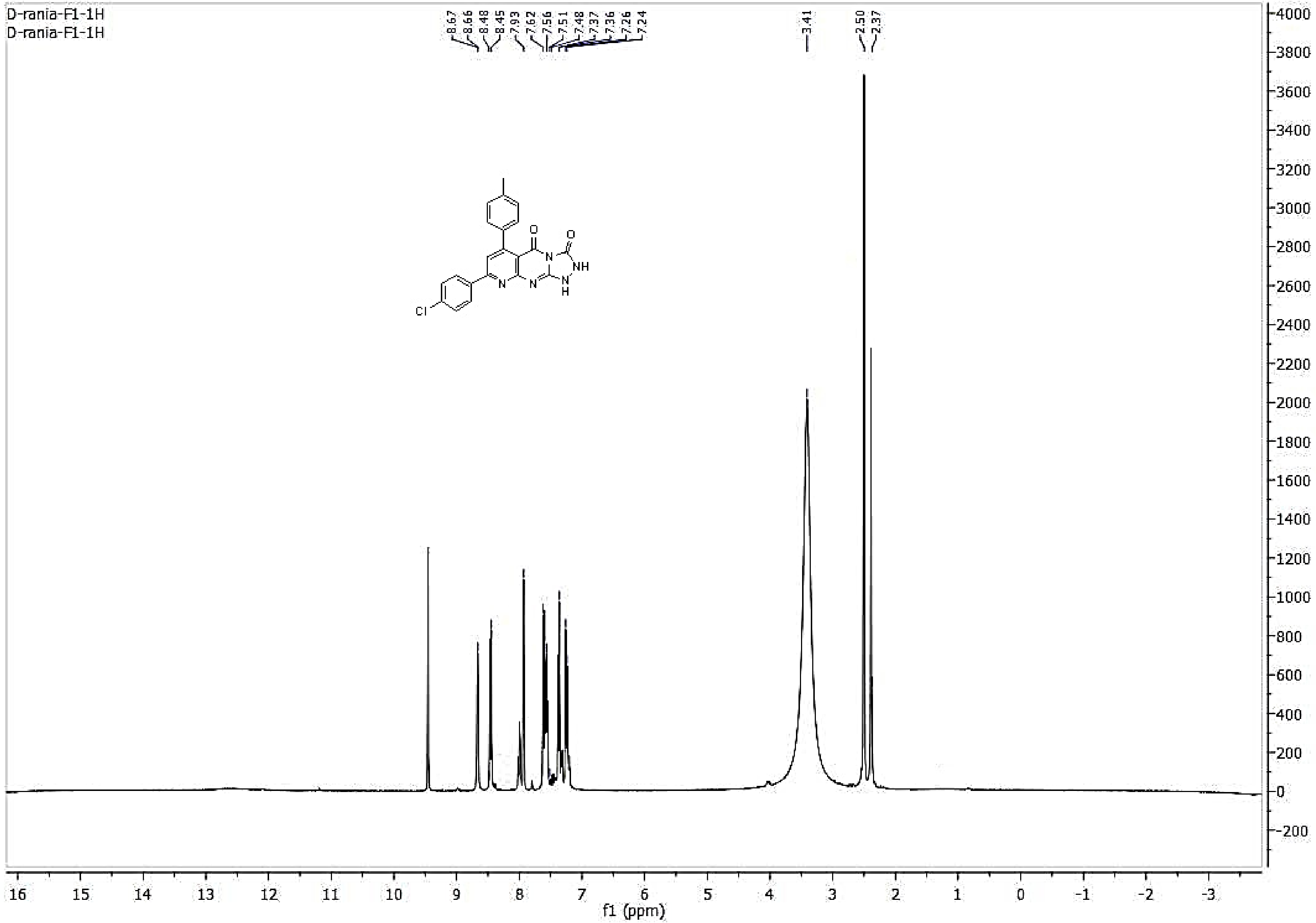

13C NMR of compound 10a

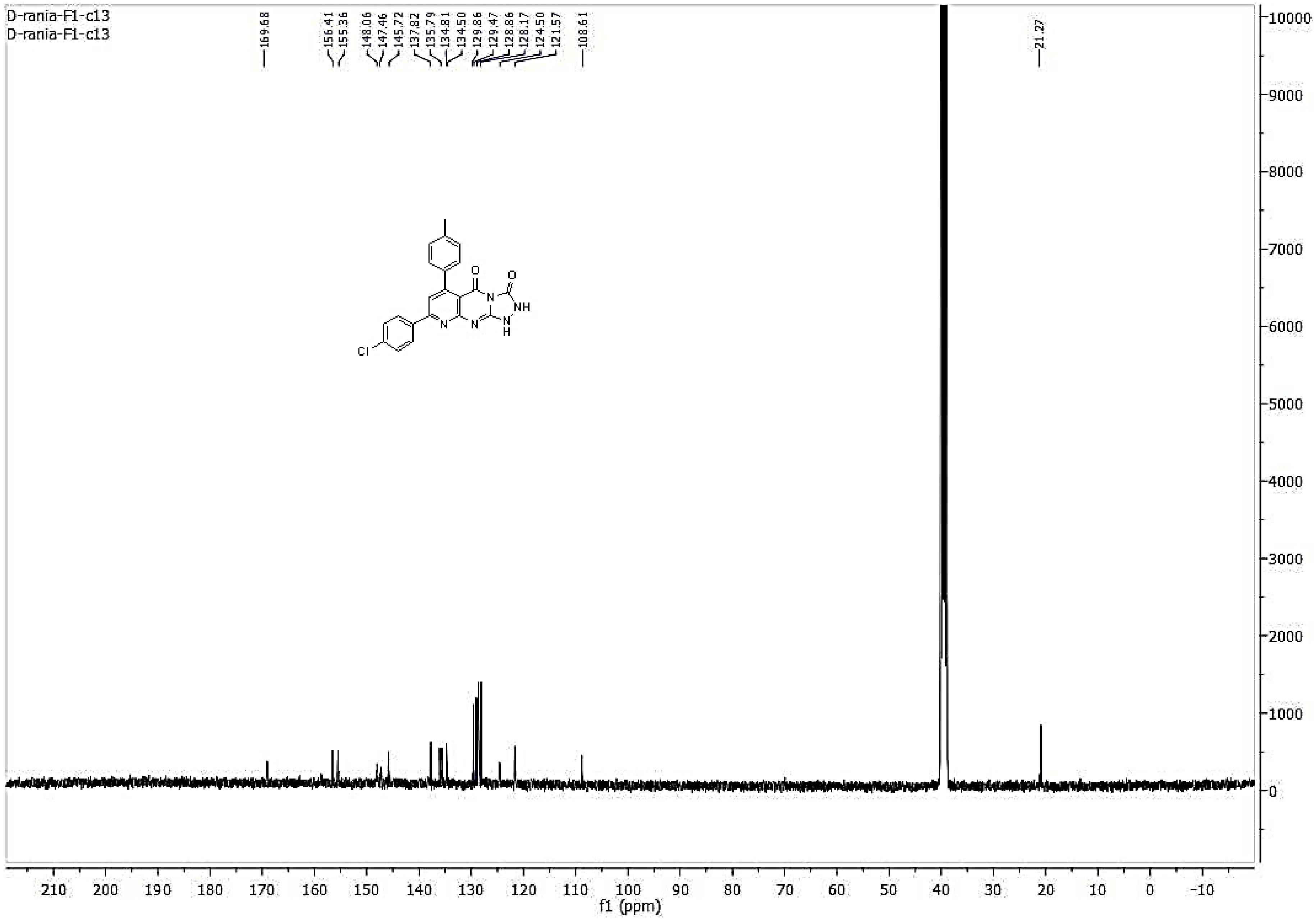

1H NMR of compound 10b

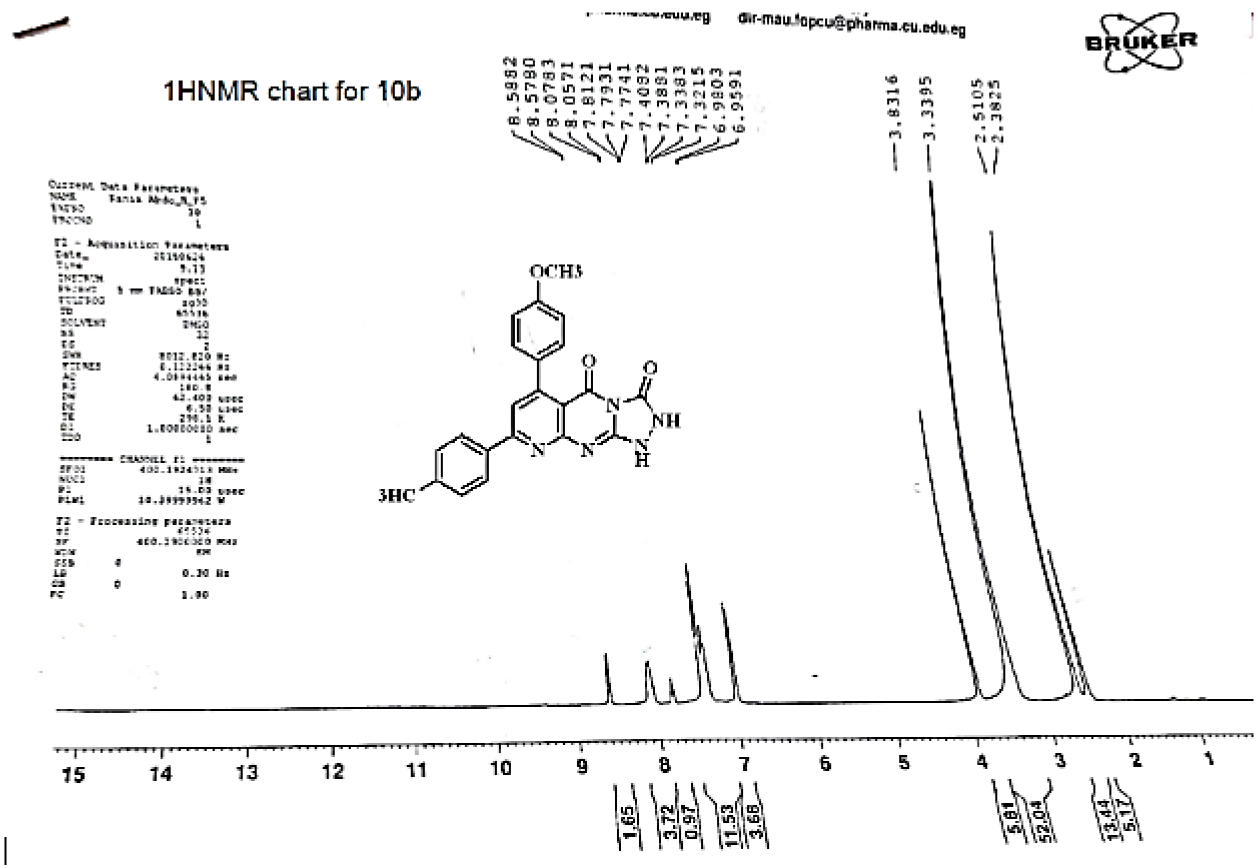

1H NMR of compound 10c

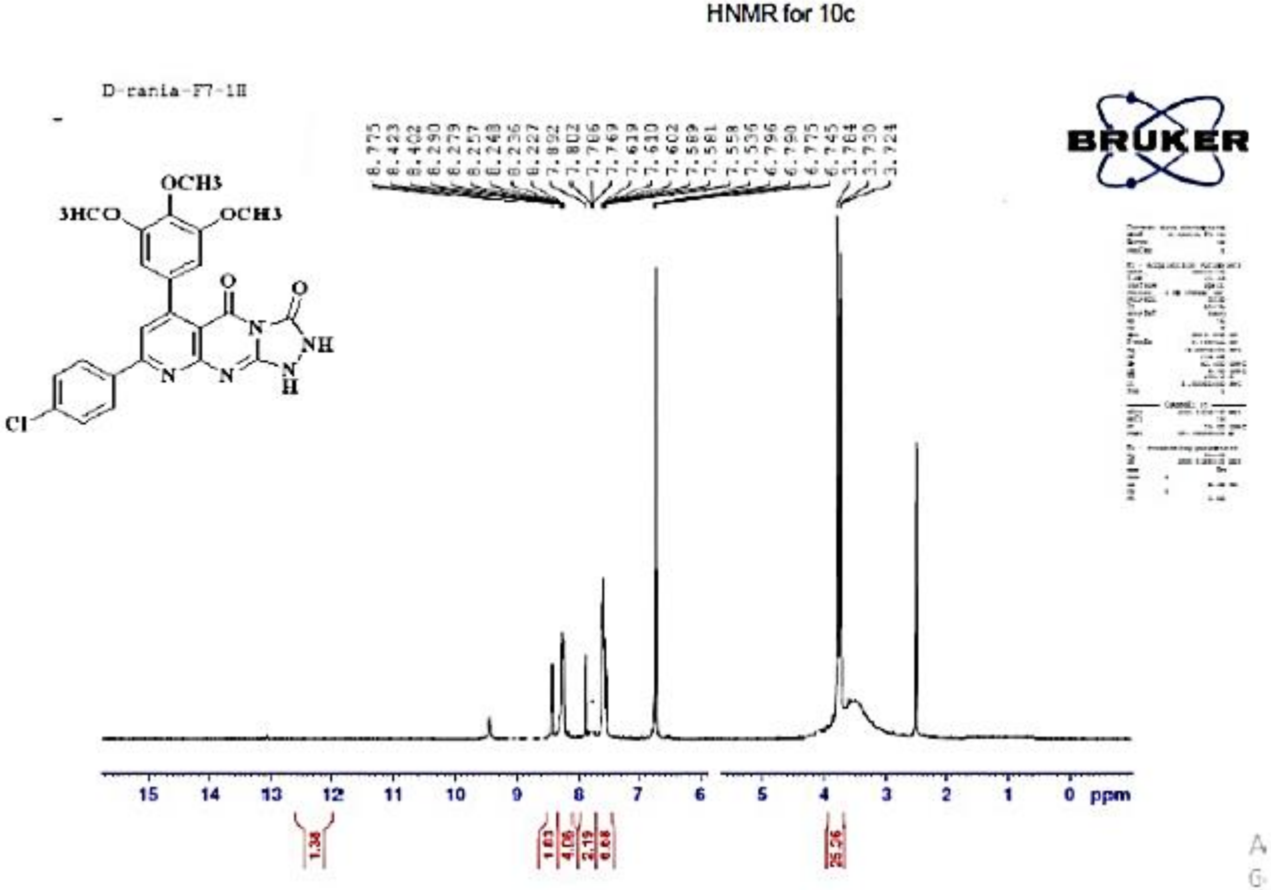

13C NMR of compound 10c

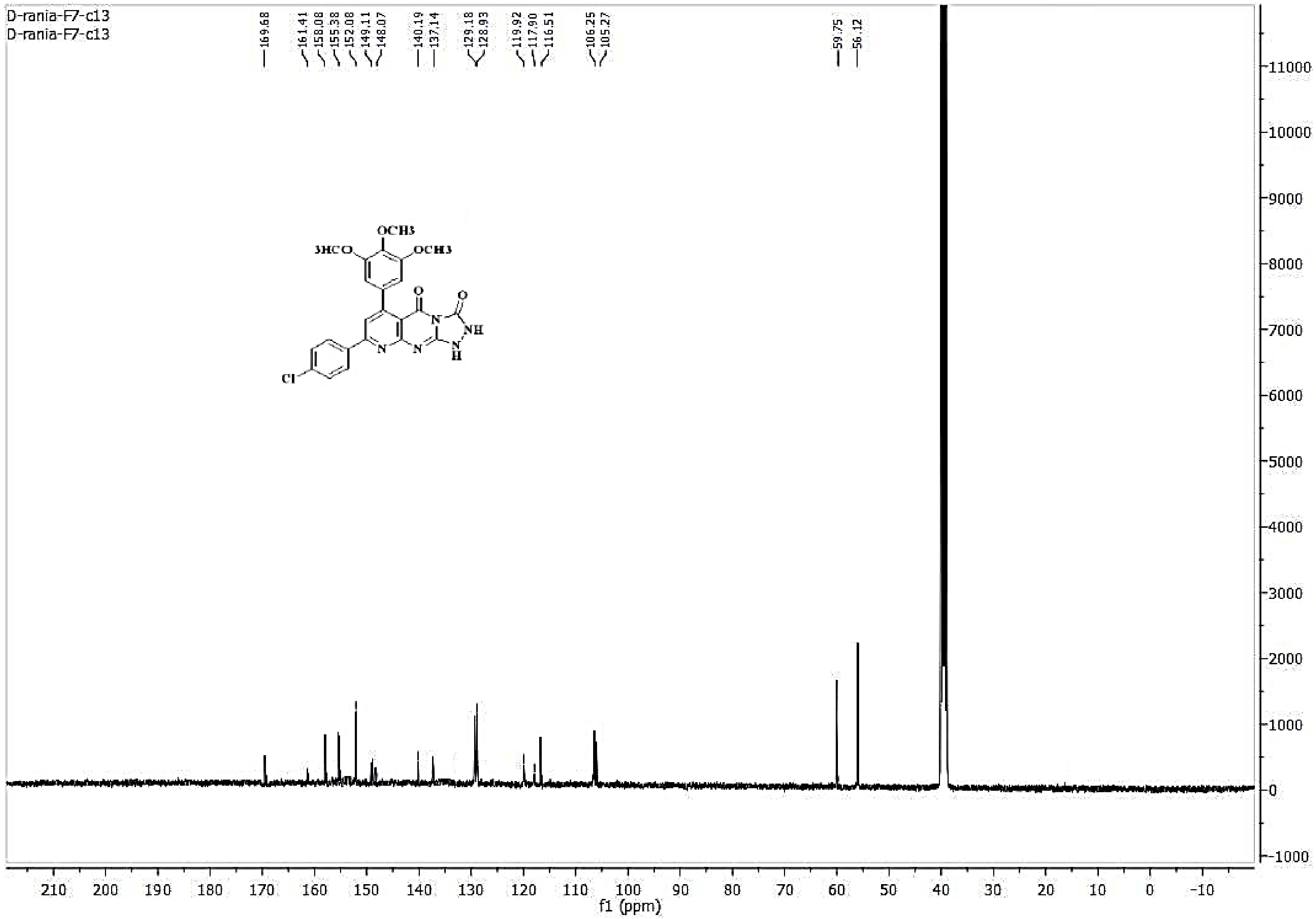

1H NMR of compound 10d

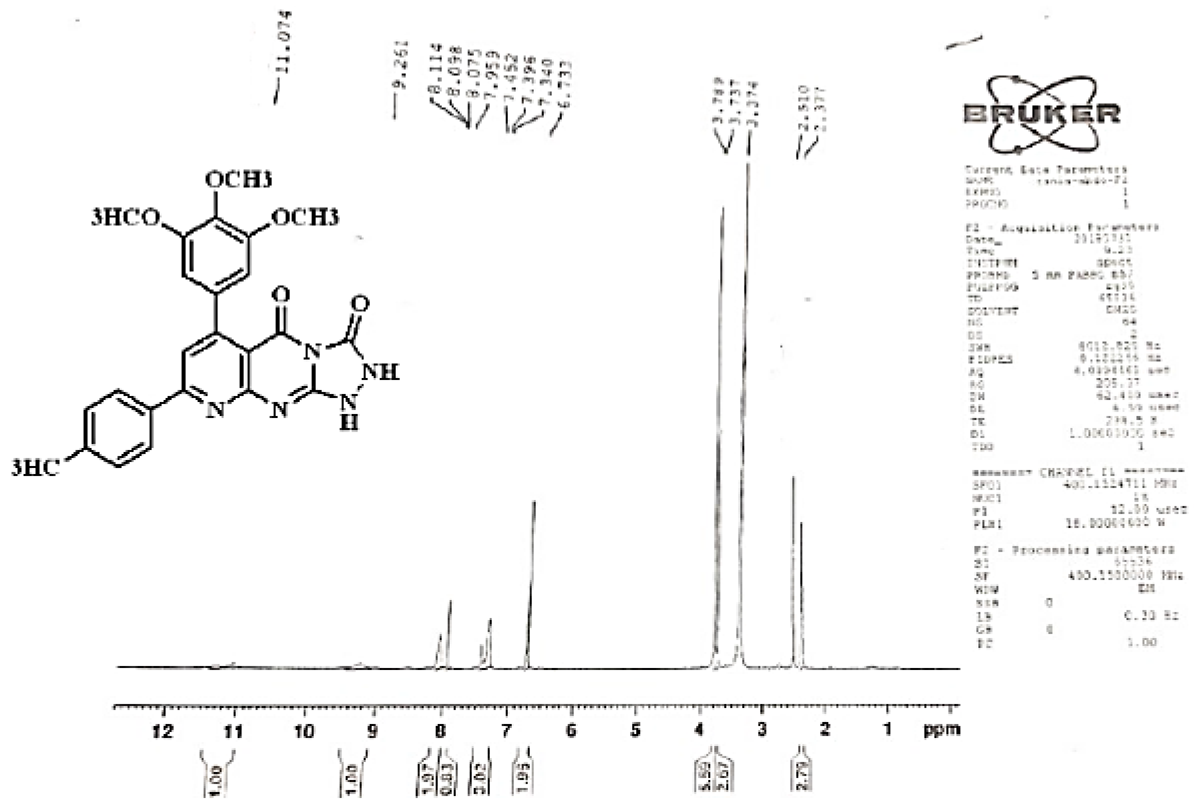

1H NMR of compound 11a

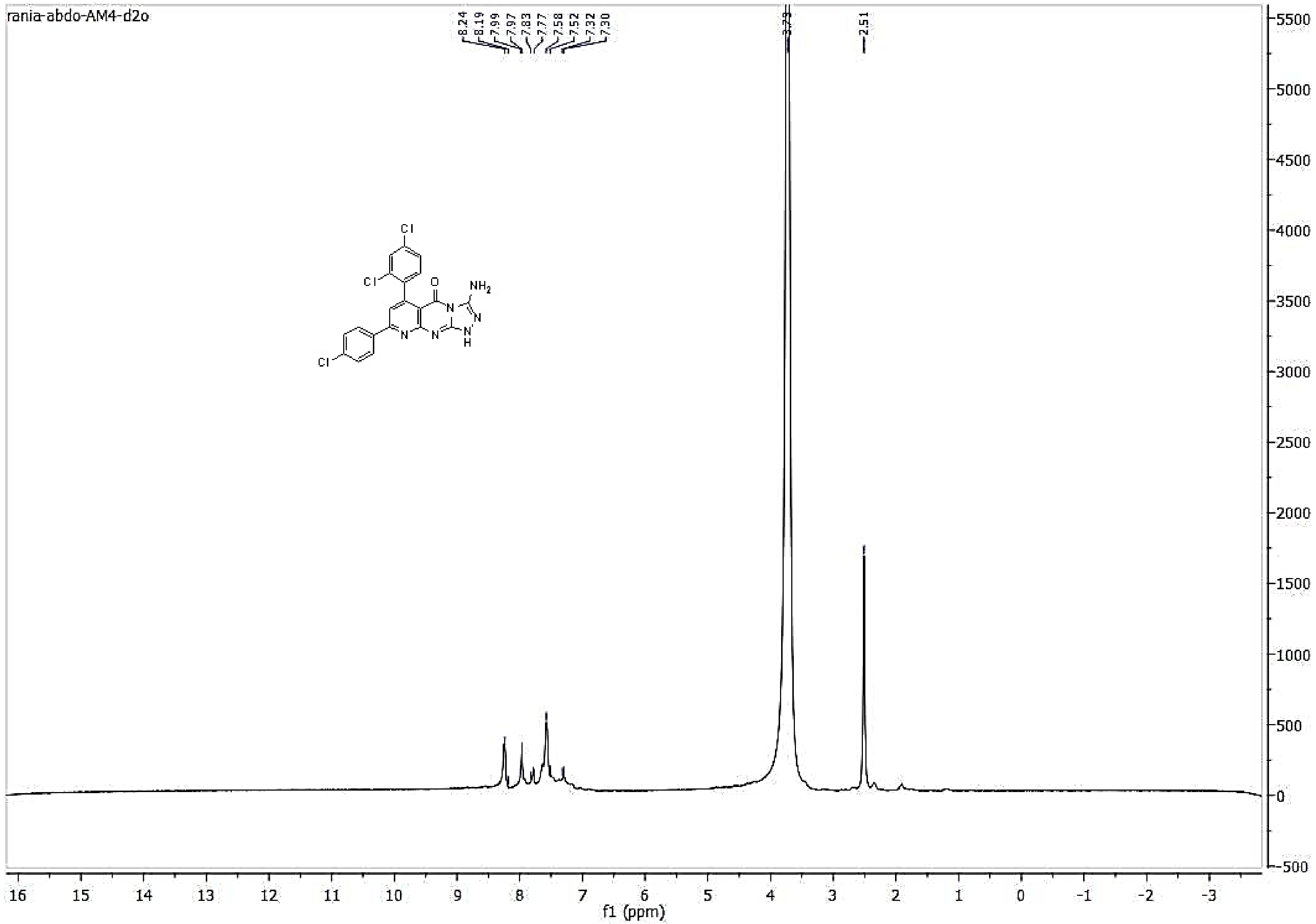

13C NMR of compound 11a

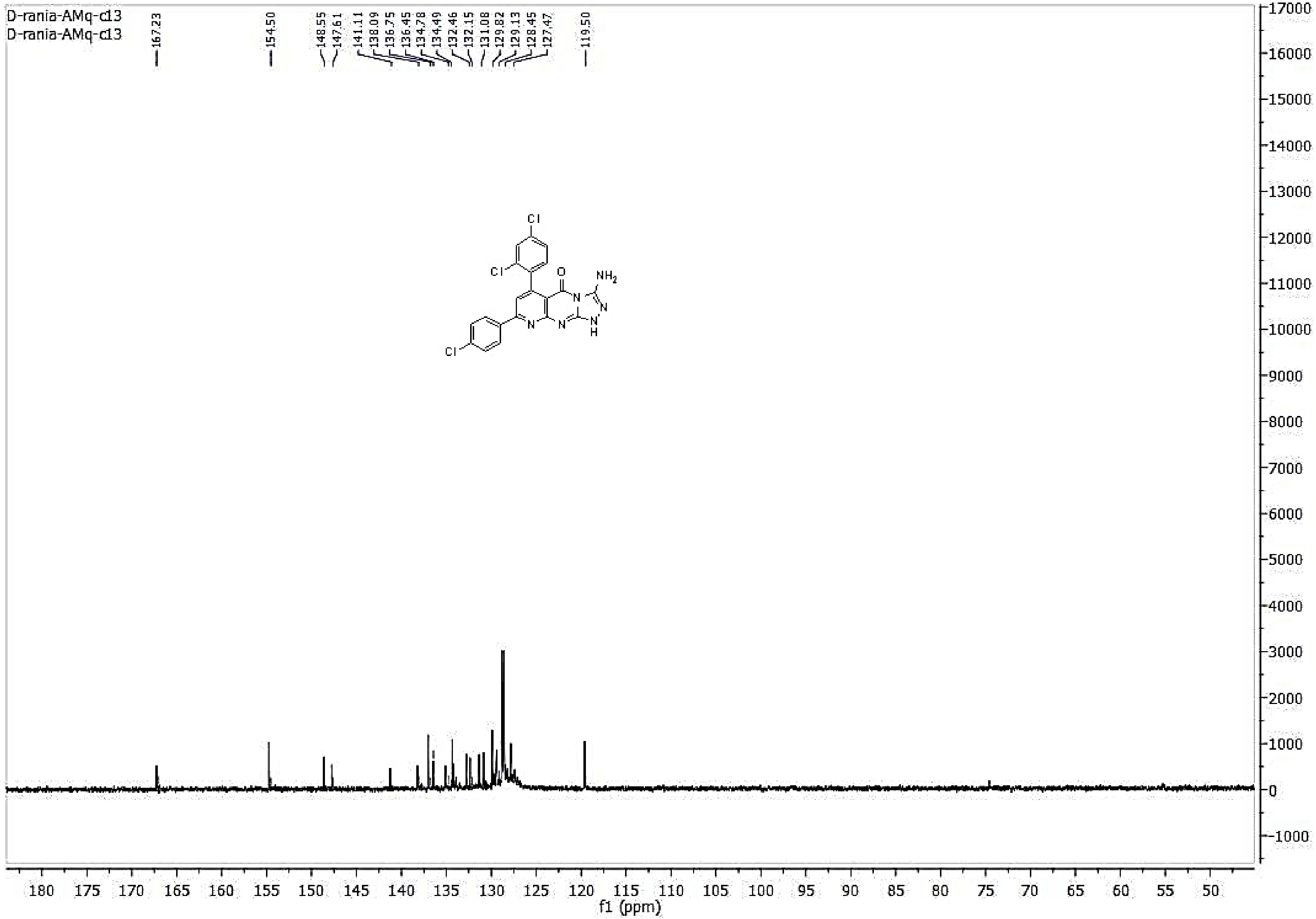

1H NMR of compound 11b

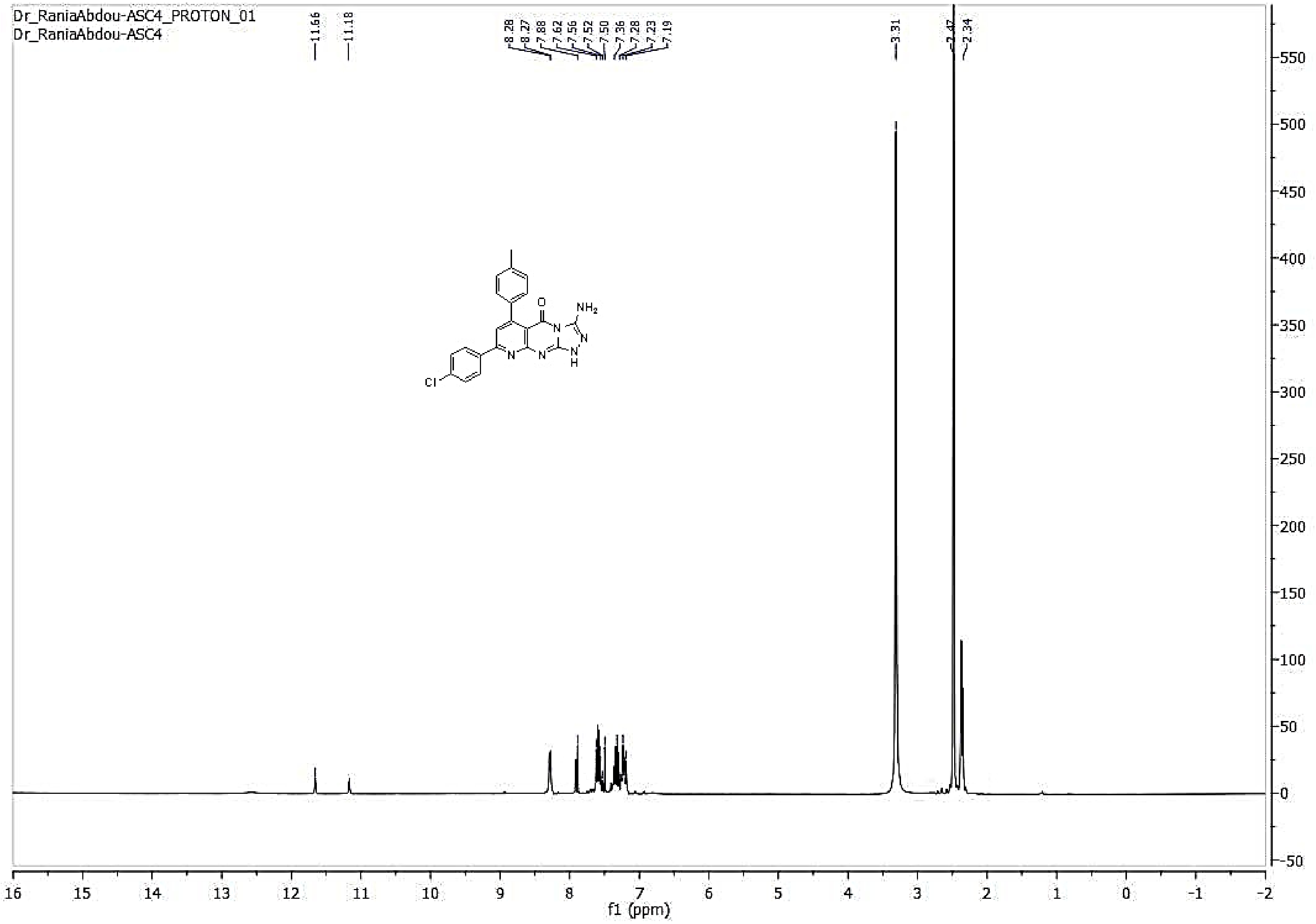

1H NMR of compound 11c

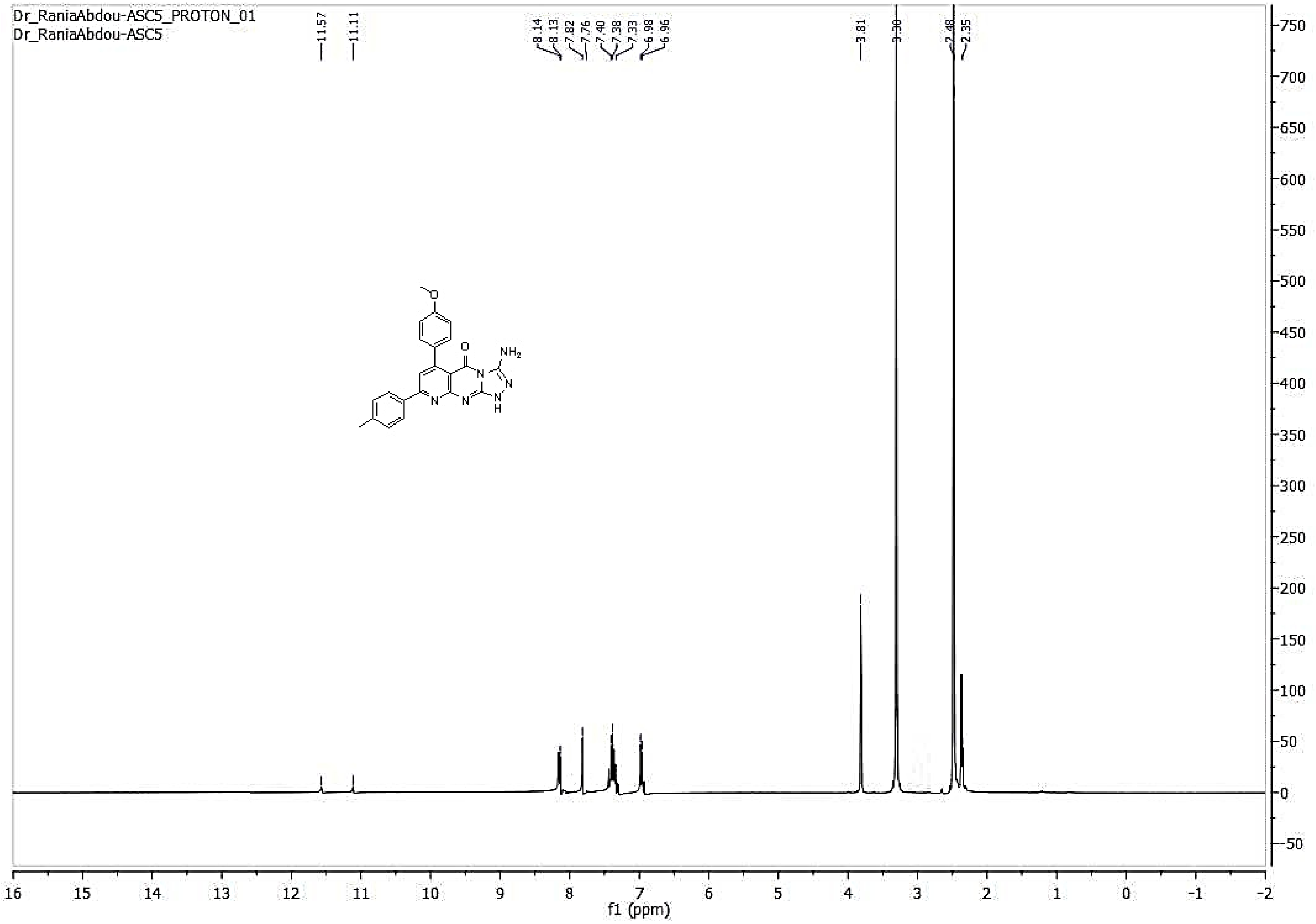

13C NMR of compound 11c

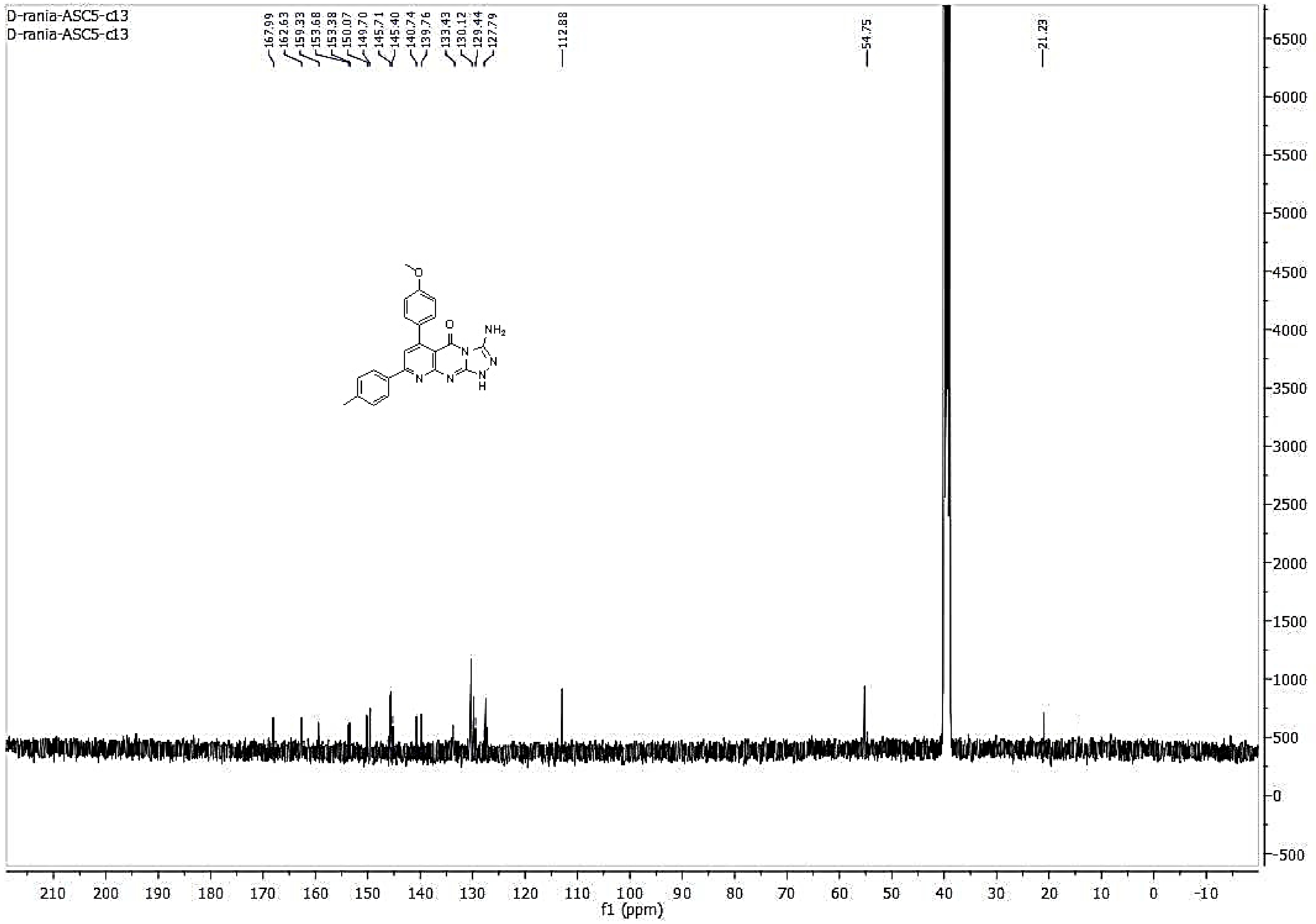

1H NMR of compound 11d

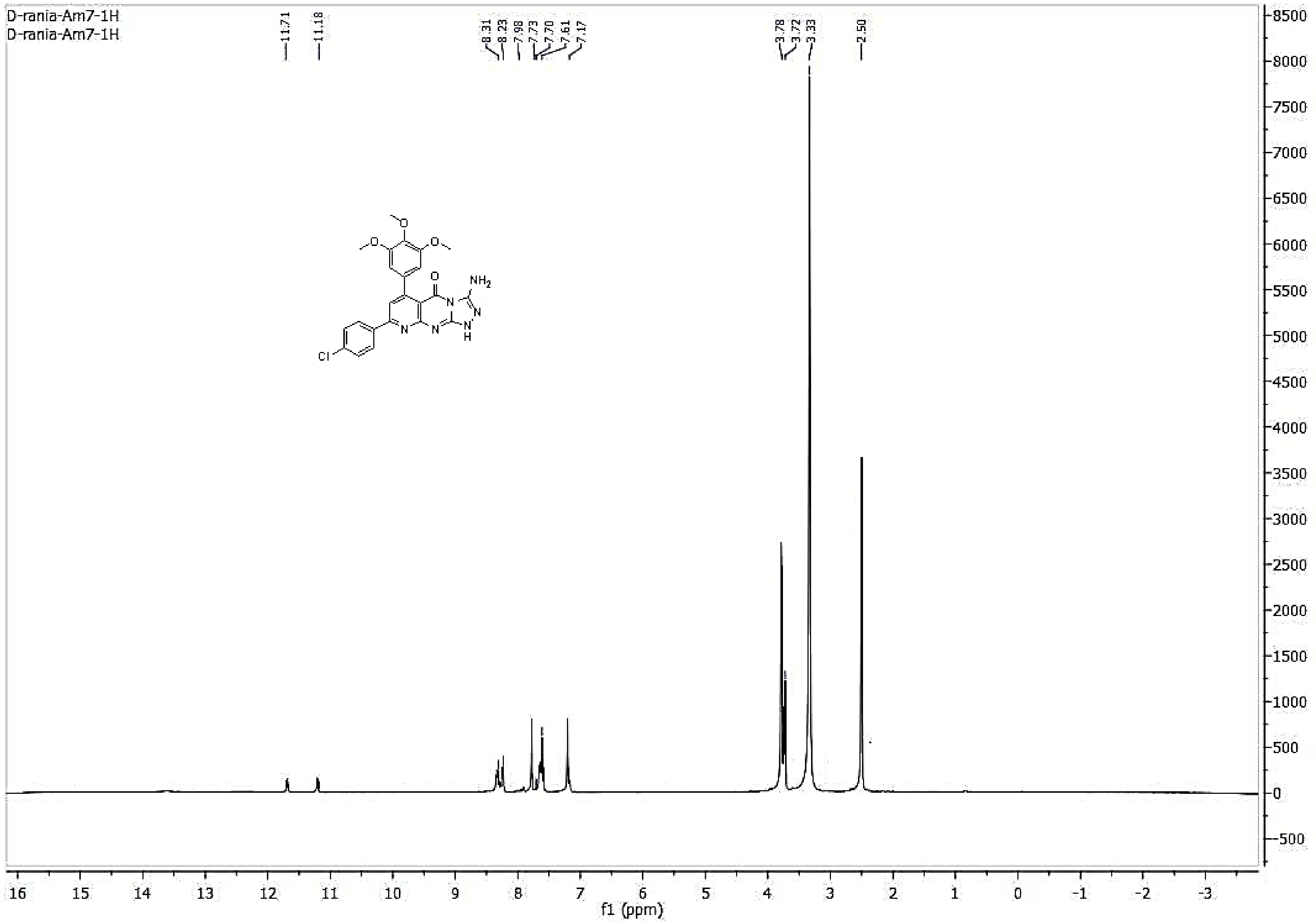

13C NMR of compound 11d

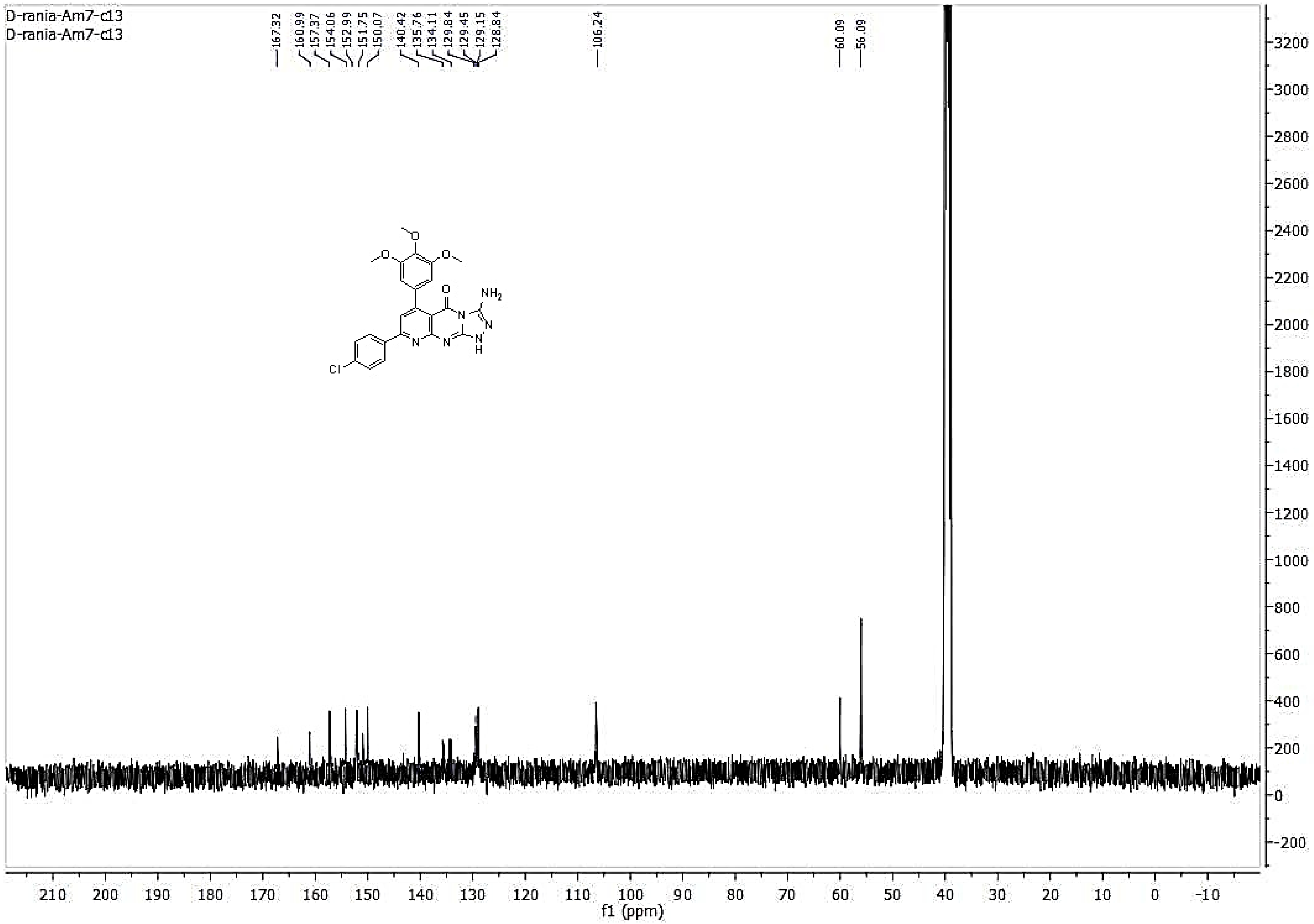

1H NMR of compound 11e

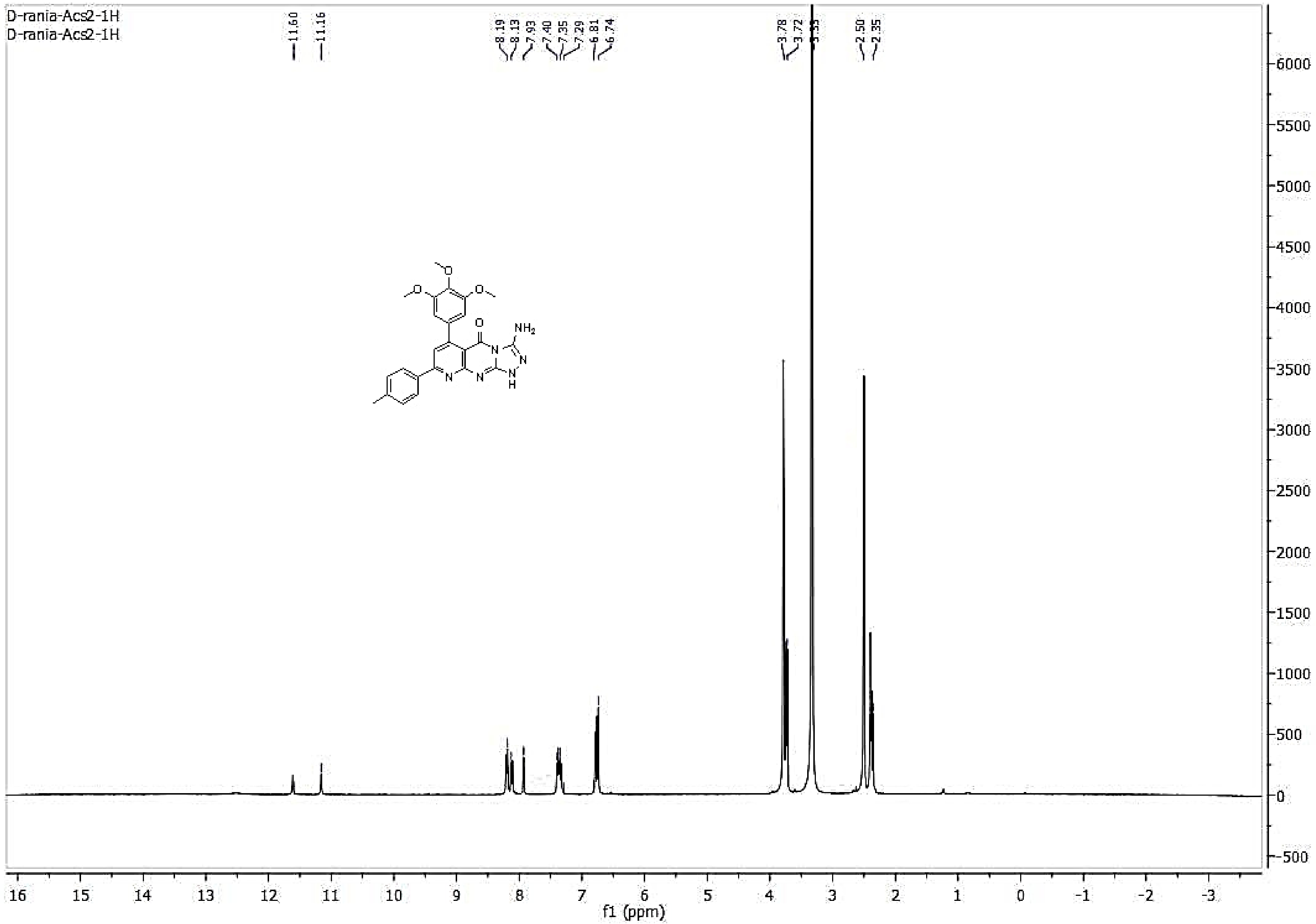

13C NMR of compound 11e

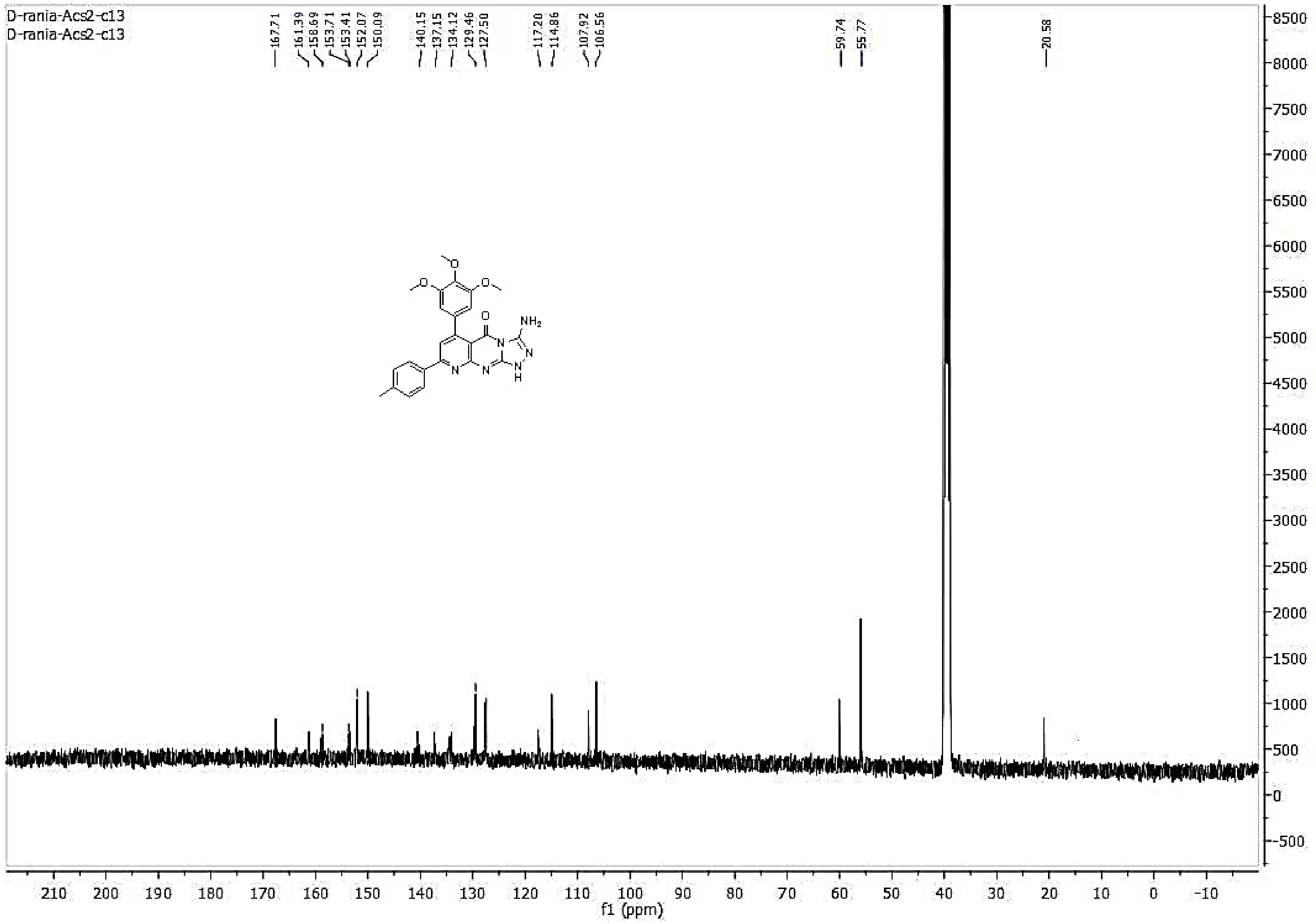

1H NMR of compound 12a

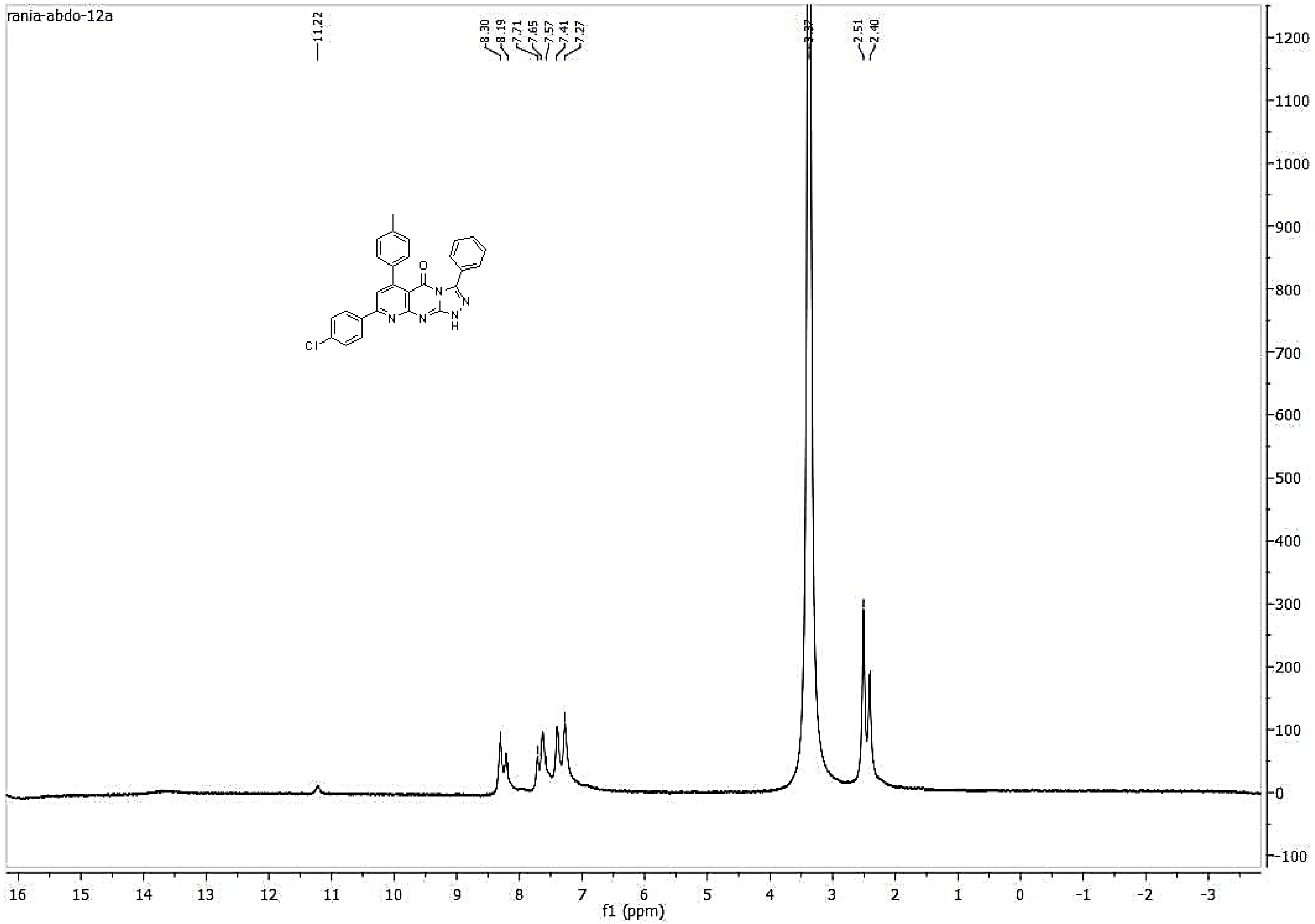

13C NMR of compound 12a

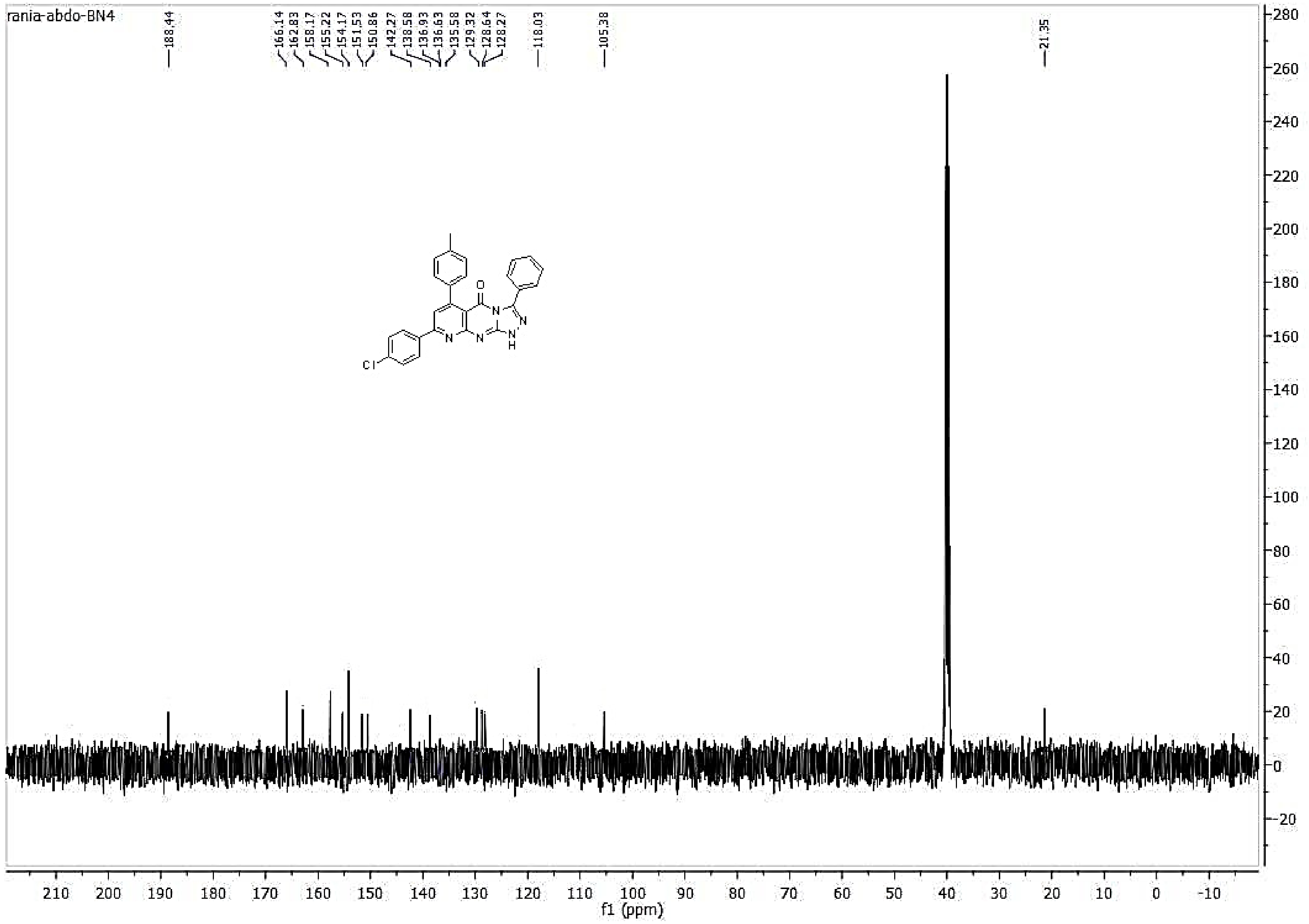

1H NMR of compound 11b

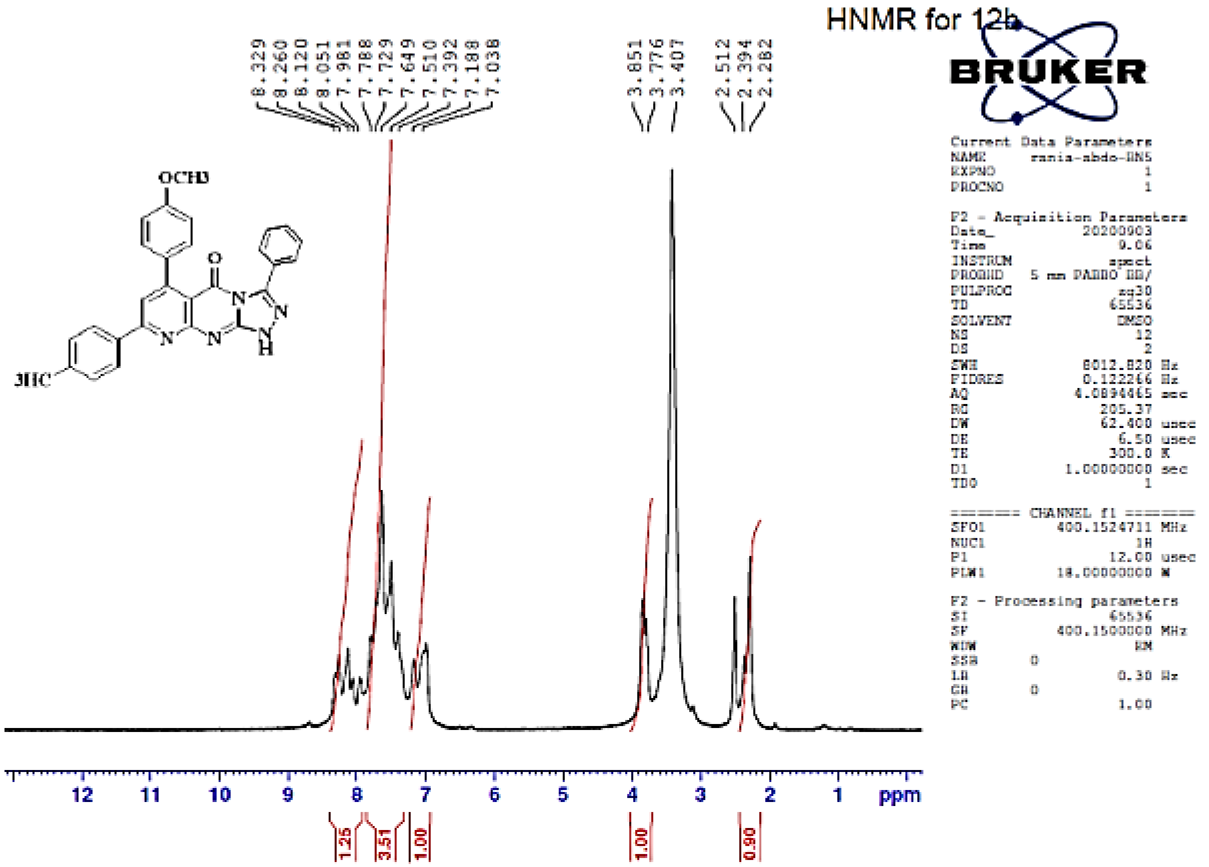

1H NMR of compound 11c

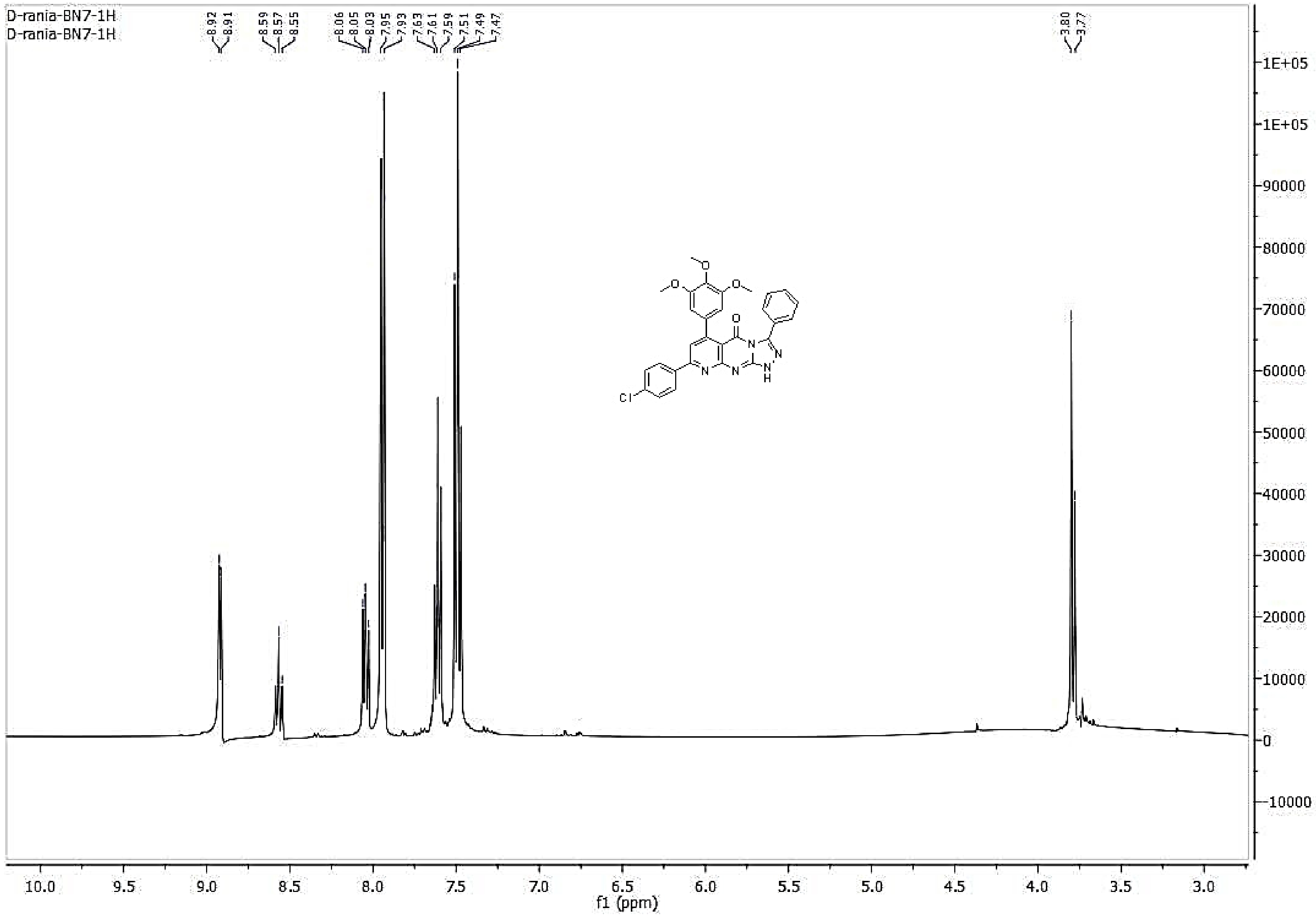

13C NMR of compound 11c

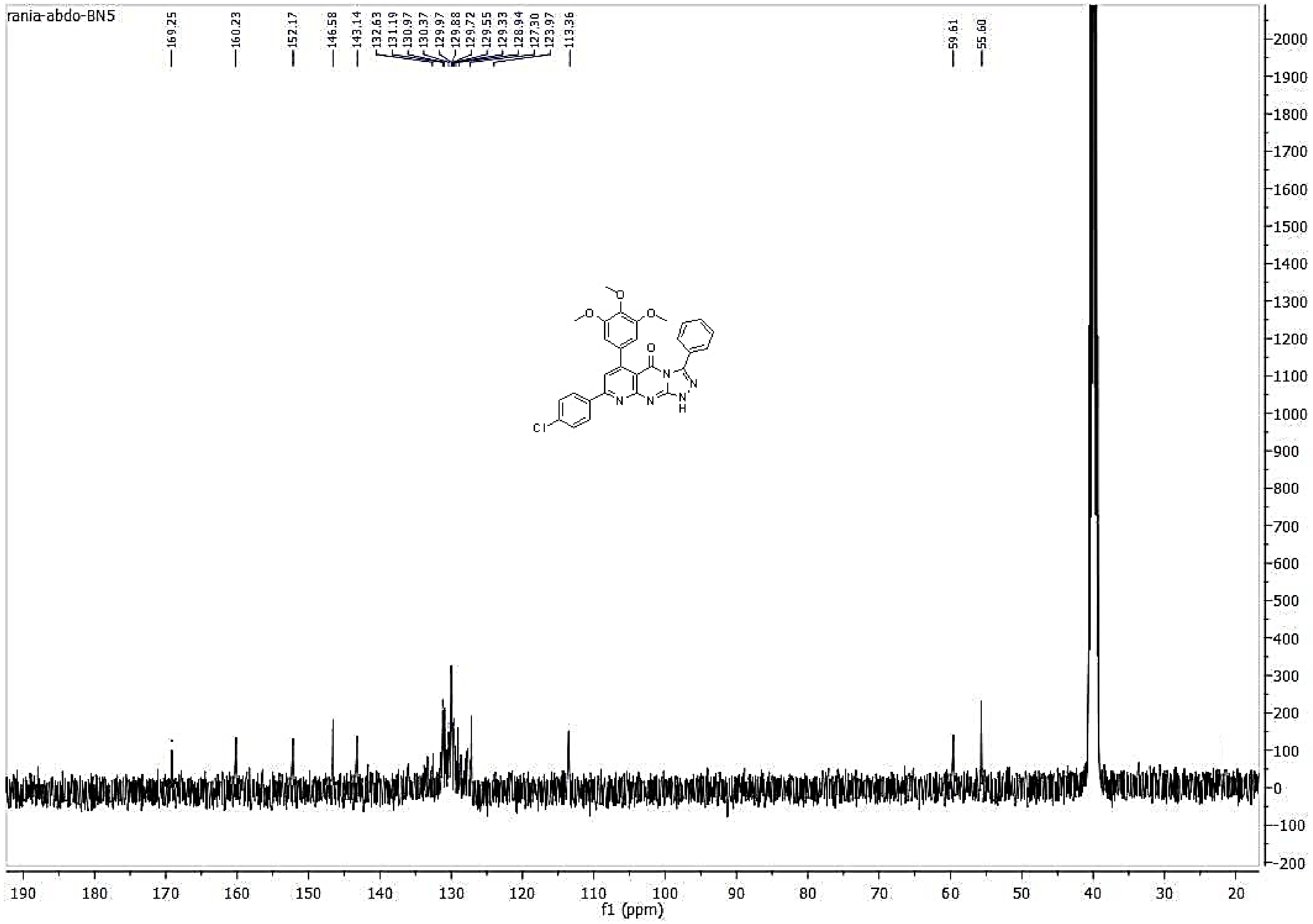

1H NMR of compound 11d

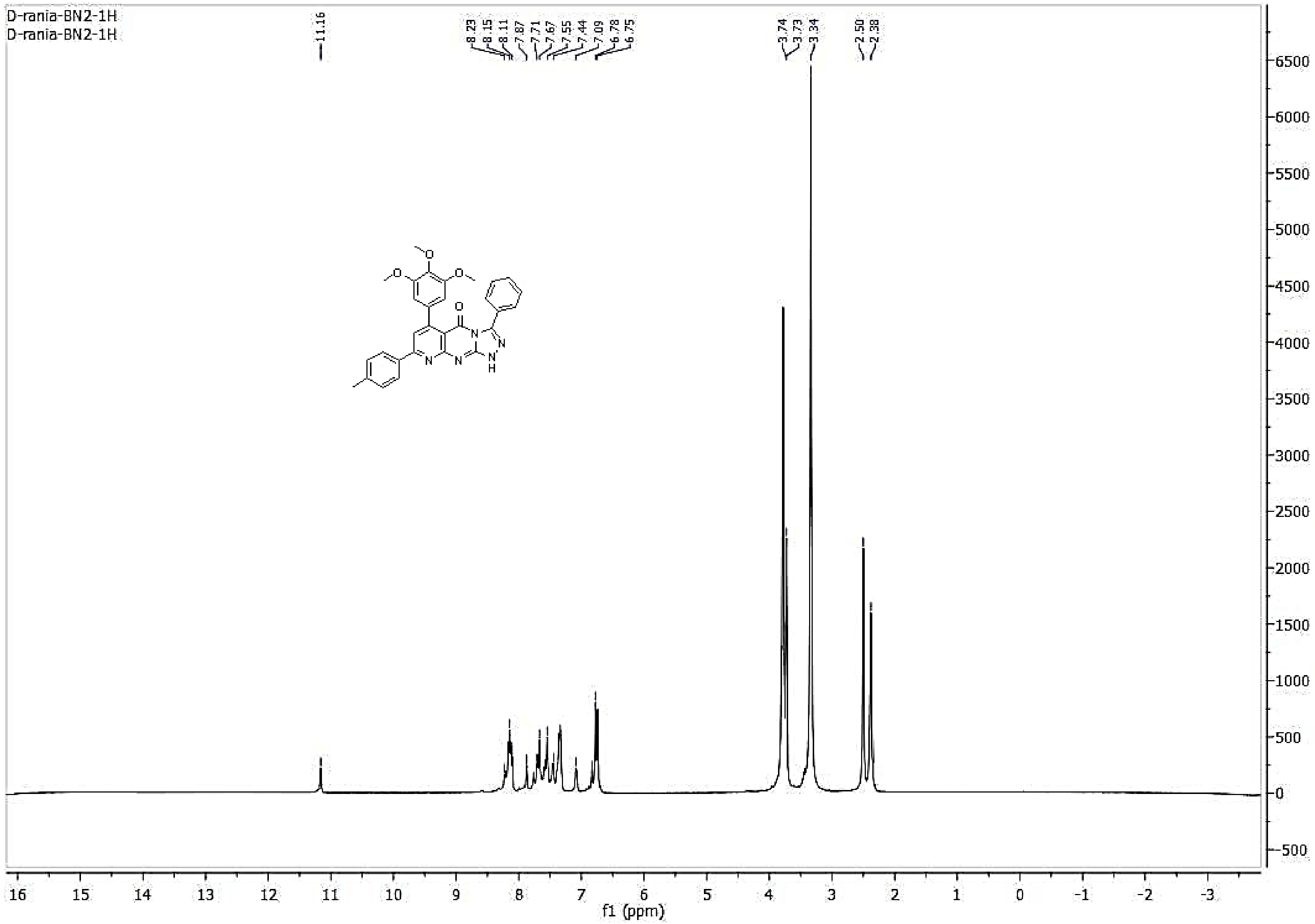

13C NMR of compound 11d

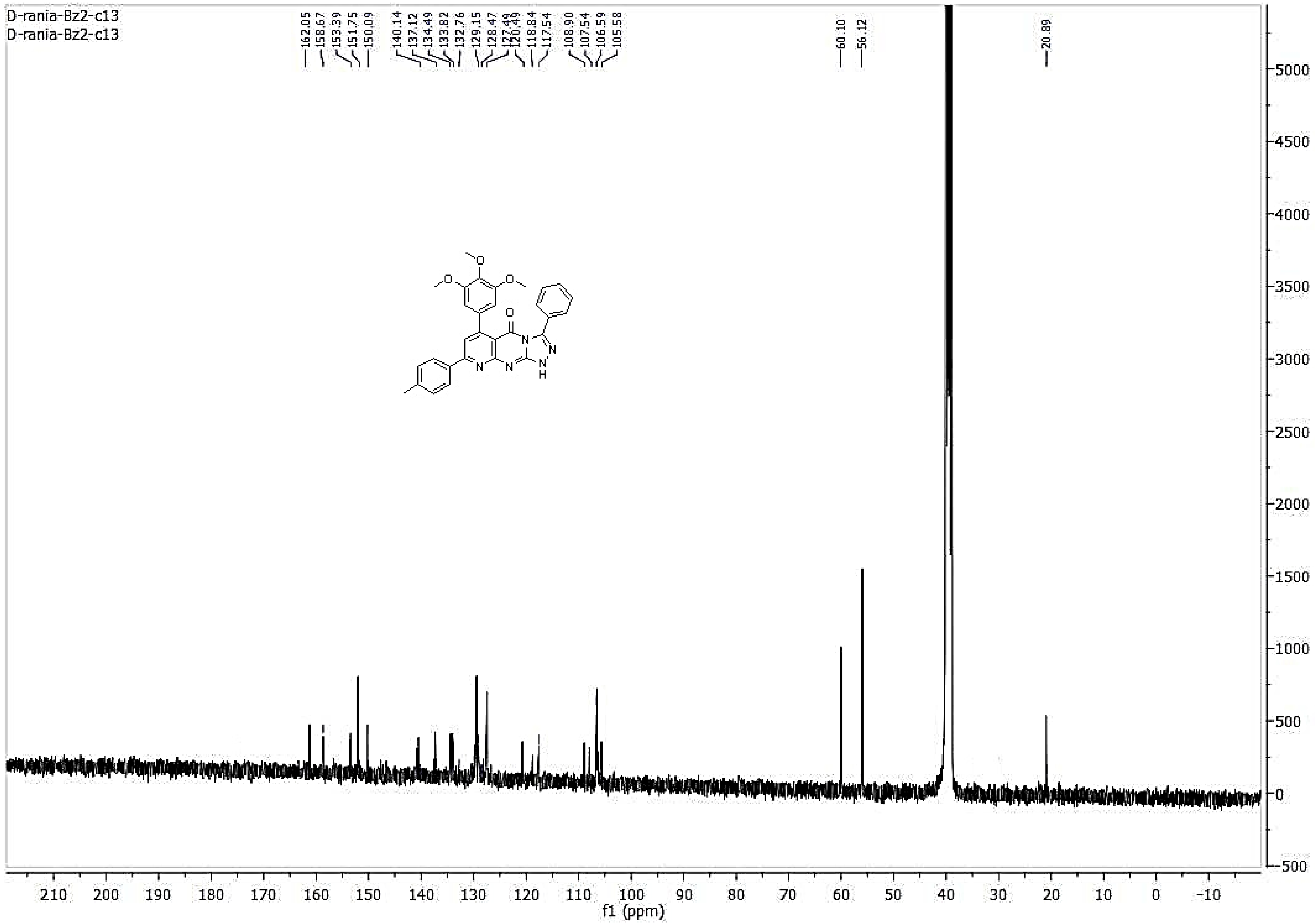

Supplement: Supplemental Material [file IENZ_A_2062752_SM3243.pdf]
